# Patient- and clinician-reported acute radiation-induced diarrhoea in patients with prostate cancer during curative external radiation therapy: A prospective observational cohort study

**DOI:** 10.1186/s41687-025-00957-3

**Published:** 2025-12-24

**Authors:** Mette Overgaard Holm, Ursula Falkmer, Randi Tobberup, Martin Skovmos Nielsen, Bjarke Mortensen, Rasmus Froberg Brøndum, Christine Vestergård Madsen, Henrik Højgaard Rasmussen, Mette Karen Yilmaz, Jimmi Søndergaard, Mette Moe, Laurids Østergaard Poulsen

**Affiliations:** 1 Center for Nutrition and Intestinal Failure, Department of Gastroenterology, Aalborg University Hospital, Aalborg, Denmark; 2 Department of Oncology, Clinical Cancer Research Center, Aalborg University Hospital, Aalborg, Denmark; 3 Department of Clinical Medicine, Aalborg University, Aalborg, Denmark; 4 Danish Nutrition Science Center, Aalborg University Hospital, Aalborg, Denmark; 5 Department for Medical Physics, Aalborg University Hospital, Aalborg, Denmark; 6 Department for Medical Physics, University Hospital of Southern Denmark, Vejle Hospital, Vejle, Denmark; 7 Center for Clinical Data Science, Aalborg University Hospital and Aalborg University, Aalborg, Denmark; 8 Department of Oncology, University Hospital of Southern Denmark, Vejle Hospital, Vejle, Denmark

**Keywords:** Patient-reported outcome measures, Diary, Weight changes, Diagnostic tool

## Abstract

**Background:**

Acute radiation-induced diarrhoea (RID) typically develops from gut mucosa inflammation within 2 weeks of external beam radiation therapy (EBRT) to the pelvis. The Common Terminology Criteria for Adverse Events (CTCAE) commonly assesses RID. In patients with prostate cancer undergoing curative EBRT, acute RID was evaluated using both a clinician-reported outcome (ClinRO)-CTCAE, and a patient-reported (PRO) stool diary.

**Methods:**

During 2022–2024, 40 newly diagnosed patients with prostate cancer receiving curative EBRT were enrolled at the Oncology Departments in two centres. CTCAE was assessed at baseline (week 1), at end of EBRT (week 8 or 9), 2 weeks after end of EBRT (week 10 or 11), and 8 weeks after end of EBRT (week 16 or 17). Simultaneously, patients maintained a daily PRO diary from baseline to week 10, and at week 16. Patients’ registration of stool frequency was converted to grades according to the CTCAE criteria.

**Results:**

The clinicians assessed all 40 patients with RID Grade 0/1 using CTCAE, whereas the PRO stool diary showed 29/40 with Grade 0/1, 6/40 with Grade 2, and 5/40 with Grade 3. Grade 2 appeared in week 2 and Grade 3 in week 4. There was a significant difference (*p* < 0.001) between CTCAE and PRO grades from baseline to week 16 or 17. No correlation was observed between irradiation data and RID Grades 2/3. Significant weight loss differences were noted from baseline to week 8 or 9 between patients with RID Grades 0/1 and those with Grades 2/3.

**Conclusion:**

According to the PRO stool diary, one in four patients with prostate cancer undergoing curative EBRT experienced acute RID Grade 2/3, but none with ClinRO CTCAE. A PRO stool diary effectively monitored the onset, course of acute RID, and body weight changes. The CTCAE does not adequately assess acute RID and needs PRO stool diary supplementation.

**Trial registration:**

Clinicaltrials.gov, NCT05684432. Registered 5 January 2023.

**Supplementary Information:**

The online version contains supplementary material available at 10.1186/s41687-025-00957-3.

## Abstract (plain language summary)

Prostate cancer is the most common cancer in men. Radiation therapy can often cure the patients. Some of them experience diarrhoea with more than 3 watery stools daily. Commonly, the diarrhoea is assessed by the clinician. This study compares patient-reported diarrhoea with the clinician assessment. During treatment and 8 or 9 weeks after, the patients noted the stool frequency and consistency. One in four patients reported acute diarrhoea, which was not captured by the clinician assessment. Therefore, the patients’ reports need to supplement the clinicians’ assessment in diagnosing and preventing acute diarrhoea.

**Supplementary Information:**

The online version contains supplementary material available at 10.1186/s41687-025-00957-3.

## Background

Acute radiation-induced diarrhoea (RID) is caused by an acute inflammatory reaction of the normal gut mucosa and typically occurs 2 weeks following initiation of external beam radiation therapy (EBRT) to carcinomas in the pelvis [1]. According to most definitions, diarrhoea is diagnosed with a frequency of ≥ 3 loose, liquid, or unformed stools daily or more frequent stools than usual [2, 3].

When stool patterns are diagnosed using a validated tool, the Bristol Stool Chart (BSC) is most commonly employed [4, 5]. In the BSC the patient records stool consistency and frequency of bowel movements but does not report other bowel-related symptoms such as urge, incontinence, or other sensations. Oncologists often use the Common Terminology Criteria for Adverse Events (CTCAE) scale, which grades the most common adverse events severity from Grades 1 to 5 [6].

In the clinical settings, diagnosing acute RID of Grade ≥ 2 according to the CTCAE is crucial for timely intervention, including antidiarrheal treatment or other clinical interventions.

A recent systematic review on RID concluded that defecation problems and their measurement tools vary widely, complicating the estimate of the frequency and type of RID, and healthcare professionals find it challenging to diagnose bowel habits of RID [7]. Therefore, better tools are required to diagnose defecation problems.

A systematic review has demonstrated that using PROM enhances focus on patients’ clinical status, leading to improved symptom control and patient satisfaction, as well as more supportive care in oncology clinics [8]. A recent study regarding patient follow-up following colorectal cancer surgery has shown that PROM data can effectively identify bowel problems, such as flatulence, diarrhoea, and stool frequency [9], early in the postoperative course. In another study comparing self-reported BSC data to faecal consistency measured by stool water content in patients with irritable bowel syndrome, modest conformity was reported [10]. Thus, PROM data could assess stool frequency and type.

Existing tools, such as St. Mark’s incontinence score (Vaizey score) [11] and the Wexner score [12], are primarily developed for patients with chronic bowel-related symptoms. However, assessing acute RID in patients undergoing curative EBRT for prostate cancer requires a low-burden, patient-reported stool diary to capture daily stools and bowel-related symptoms. This study aimed to compare the assessments of acute RID in these patients using both ClinRO-CTCAE and a PRO stool diary.

## Methods

### Study design

An observational multicentre study was conducted at the Department of Oncology, Aalborg University Hospital and University Hospital of Southern Denmark, Vejle Hospital. The inclusion period was from June 2022 to March 2024.

### Sample size calculation

A sample size calculation for this explorative study is uncertain, and therefore it was not done. Comparing the results obtained by the well-known tool (CTCAE) and the newly developed patient-reported tool (stool diary) is explorative. In the study protocol registered in Clinicaltrials.gov, NCT05684432 the number of patients with prostate cancer was estimated to at least 50. In a recent comparable study of 88 Danish patients post-EBRT diarrhoea was 19% [13]. After enrolling 20 patients with prostate cancer, it became apparent that the study’s endpoint, namely the frequency and grade of acute RID, could be identified using the patient-reported stool diary. In these 20 patients the frequency of acute RID Grade 2/3 was about 25%. Also, the next 20 patients showed the same rate of frequency.

### Patients

**Fig. 1:**
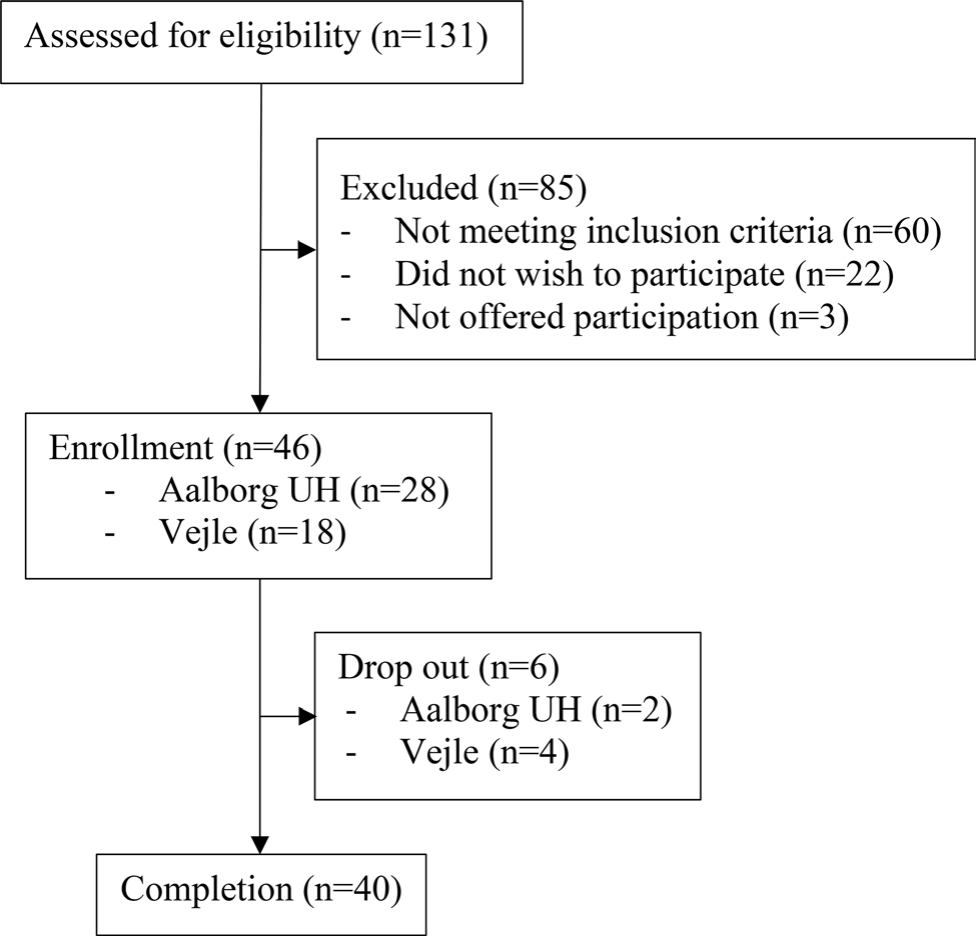
Flow chart of patient inclusion

Forty patients with newly histopathologically diagnosed prostate adenocarcinoma, who were referred to the Department of Oncology in Aalborg or in Vejle for curative EBRT, were included (Fig. 1).

All patients received androgen deprivation therapy (ADT) 3 months before the start of curative EBRT and were scheduled to continue for 36 months. The inclusion criteria included histopathologically verified prostate adenocarcinoma, an Eastern Cooperative Oncology Group performance status of 0–1, age ≥ 18 years, and the ability to understand and provide informed consent in Danish. Exclusion criteria included prior treatment with EBRT, other forms of radiation to the pelvis, or a history of diagnosed inflammatory bowel disease. If patients agreed to participate, they were informed orally and in written form and provided with informed consent.

### EBRT

Curative EBRT was administered according to the Danish Prostate Cancer group’s guidelines [14]. All patients were treated in a supine position, including immobilisation of the heel and knee, with a comfortable filled bladder and, if possible, an empty rectum. Prior to the planning scans, fiducial markers were placed in the prostate for daily image guidance. Magnetic resonance imaging (MRI) and/or computed tomography (CT) scans for calculating attenuation in the treatment position were acquired and imported into the treatment planning system (TPS). The TPS used at Aalborg were ARIA^®^ (Varian Medical Systems, Palo Alto, CA), and at Vejle RayStation^®^ TPS (RaySearch Medical Laboratories AB, Sweden). The prostate was delineated on CT and/or MRI scans with a site-specific planning target volume (PTV1) margin of 5–7 mm. The elective lymph node area was delineated according to the 2009 RTOG guidelines with a site-specific PTV2 margin of 5–7 mm [15].

In total, 40 patients received treatment with a total dose of 78 Gy, administered in daily fractions of 2 Gy from Monday to Friday, with breaks on weekends and public holidays. Thus, the entire EBRT period lasted 8–9 weeks.

## Assessments

### Clinician-reported RID by CTCAE

The frequency of acute RID was assessed according to the CTCAE, vers. 5 [6]. Assessments were made at baseline (1 month before the start of EBRT) and at end of EBRT (week 8 or 9), 2 weeks after end of EBRT (week 10 or 11), and 8 weeks after end of EBRT (week 16 or 17) (Table 1). Frequency at baseline was graded based on the patient’s habitual frequency of stools. An amendment was made for an extra measure point at week 10 but could only be made in 31 patients.

Study data were collected and managed using the Research Electronic Data Capture (REDCap) hosted at Aalborg University Hospital [16, 17].

### Patient-reported RID

The PRO stool diary (Supplement 1) was developed to report daily patient-reported acute RID as frequency and stool type according to the BSC, which is a clinical assessment tool designed to classify faeces into seven groups, among others to assess diarrhoea [4] (Table 1). The patient was encouraged to complete the PRO stool diary immediately after visiting the toilet to avoid bias. The stool frequency was reported independently of the Bristol Stool type [18], and the patients’ responses were converted to grades according to the CTCAE criteria [6].

Baseline registration was made the 1st day of EBRT, and the grading was based on the first week of EBRT. An increase of ≥ 4 stools per day over baseline was considered a critical threshold requiring treatment. For patients with no stool or with missing data on first day of EBRT, the baseline was set to the frequency of the first day of EBRT with stool frequency reported.

The PRO stool diary was distributed in paper form during the patient’s first visit to the radiation centre and was returned 8 or 9 weeks after the completion of EBRT to the study’s lead investigator. The data were entered in REDCap.

### Twenty-four-hour registration by PRO stool diary

Assessments were made at baseline (first day of EBRT), daily during EBRT (weeks 1–9), weeks 10 or 11, and week 16 or 17 (Table 1). The frequency of stools was reported according to daytime (06.00–21.59 h) and nighttime (22.00–05.59 h). A stool frequency > 2 at nighttime was considered a burden for the patient. The threshold was chosen based on the patients’ experiences.

### Urgency and faecal incontinence

Urgency and faecal incontinence were assessed according to questions 35, 41, and 44 in the EORTC QLQ-PRT20 [19]. Question 35 is asking: Have you had any unintentional release (leakage) of liquid stools?, 41: Have you been unable to wait 15 min to open your bowels?, and 44: Have you had difficulty going out of the house, because you needed to be close to a toilet, because of bowel problems? The patient indicated the extent of experienced symptoms or problems of urgency and faecal incontinence during the past week The symptoms were categorised: (1) not at all, (2) a little, (3) quite a bit, and (4) very much. Assessments were made at baseline (1 month before the start of EBRT), at end of EBRT ( (week 8 or 9), and 8 weeks after end of EBRT (week 16 or 17) (Table 1).

### Body weight and antidiarrheal medication

The patient also reported body weight and the use of antidiarrheal medication (Table 1). Body weight was reported in the PRO stool diary by the patient to the nearest 0.1 kg in the morning with light clothing, after toilet visits, and before eating and drinking.

A clinically significant weight loss is generally defined as at least 5% reduction in weight from baseline [20], but patients with cancer with a weight loss of 2.5% have shown statistically lower survival time, regardless of body mass index (BMI) [21].

**Table 1:** Study procedures

Examinations/tools		Weeks																
	Baseline*	1	2	3	4	5	6	7	8	9	10	11	12	13	14	15	16	17
EBRT**		x	x	x	x	x	x	x	x	x								
Clinical examination/CTCAE	x								x	x	x	x					x	x
PRO stool diary, incl. Bristol Stool Chart		x	x	x	x	x	x	x	x	x	x	x					x	x
Urgency measured by EORTC QLQ-PRT20	x								x	x							x	x
Body weight, antidiarrheal medication		x	x	x	x	x	x	x	x	x	x	x					x	x
Dietary intake (3-day food records)***	x								x									
Nutritional screening (PG-SGA SF)***	x								x		x						x	x
Physical activity level questionnaire (IPAQ SF)***	x								x								x	x
EORTC QLQ-C30***	x								x								x	x

*Baseline registration was made approximately 1 month before the start of EBRT with CTCAE, EORTC QLQ-PRT20, dietary intake, PG-SGA-SF, IPAQ SF, EORTC QLQ-C30, and the first day of EBRT with PRO stool diary

**A total dose of 78 Gy was administered in daily fractions of 2 Gy from Monday to Friday, with breaks on weekends and public holidays. Thus, the entire EBRT period lasted 8–9 weeks

***Dietary intake, PG-SGA SF, IPAQ SF, and EORTC QLQ-C30 data will be published separately

Abbreviations: CTCAE, Common Terminology Criteria for Adverse Events; PRO, patient-reported outcome; EORTC QLQ-PRT20, European Organisation for Research and Treatment of Cancer Quality of Life Questionnaire – Proctitis Module (PRT20); PG-SGA SF, Patient-Generated Subjective Global Assessment - Short Form; IPAQ SF, International Physical Activity Questionnaire - Short Form; EORTC QLQ-C30, European Organisation for Research and Treatment of Cancer Quality of Life Questionnaire - Core 30

There is missing data at week 10 for the first nine included patients

## Grading

### Grading by the CTCAE scale

Grade 0: No increase in stools compared to baseline during the week before registration. Grade 1: Increase of < 4 stools per day over baseline on one or more days during the week before registration. Grade 2: Increase of 4–6 stools per day over baseline on one or more days during the week before registration. Grade 3: Increase of ≥ 7 stools per day over baseline on one or more days during the week before registration [6]. The grade at baseline was assessed according to frequency during the last week.

### Grading by the PRO stool diary

The responsible investigator reviewed the PRO stool diary with the patient, either in person or by phone. The responsible investigator graded the stool frequency in the PRO stool diary according to the CTCAE criteria, as described above, based on the day with the highest frequency during the preceding week.

### Radiation therapy dose-volume data

Extracted dosimetric data from the TPS was the dose constrains according to doses-volume points matching the planning goals. For the rectum the data was *V*_50Gy_, *V*_60Gy_, *V*_70Gy_, and BowelBag *V*_45Gy_ were extracted from ARIA^®^ and RayStation^®^.

### Data analysis and statistics

Data was exported from REDCap to R version 4.4.0 [22].

Comparison of CTCAE and PRO stool diary data, including 24-h data, were visualised for descriptive analysis using *ggplot2* [23].

Fisher’s exact test was used to analyse differences in the distribution of RID grade (CTCAE vs. PRO stool diary), and the association between urgency and faecal incontinence and PRO RID grade.

The correlation between BowelBag *V*_45Gy_, *V*_50Gy_, *V*_60Gy_, *V*_70Gy_, and the patients’ RID Grade (0/1 vs. 2/3) was assessed using a Wilcoxon test. Relative changes in body weight were illustrated using waterfall plots and boxplots. Differences in body weight change across grades of acute RID were assessed pairwise (0 vs. 1; 0 vs. 2/3, 1 vs. 2/3) using a Wilcoxon test. Descriptive data are presented as count and percentage for categorical variables, and median (range), mean, and standard deviation for continuous variables. For all statistical tests, a p-value < 0.05 was considered significant.

### Data protection

All data was handled according to the rules of Good Clinical Research Practice [24]. The research database was stored at the Clinical Research Unit, Dept. of Oncology, Aalborg University Hospital and owned by the Northern Region of Denmark, where the study was registered (2021 − 229). The password was established solely for the principal investigator. The project group members were handling the data in accordance with Danish laws on personal data protection. The patient’s personal identification (CPR number) was removed and replaced with a code number (pseudonymisation).

## Results

Table 2 shows patients’ characteristics and EBRT data.

**Table 2:** Patient characteristics and EBRT data

**Patients (** ***n*** **)**	40
**Age**,** median in years (range)**	71 (48–80)
**Body weight**,** median in kg (range)**	84 (70–130)
**BMI**,** median in kg/m**^**2**^ **(range)**	27 (23–43)
<18.5	0
18.5–24.9	11
25.0-29.9	19
≥30.0	10
**Smoking**	
Current smoker	5
Former smoker	19
Never smoker	16
**Charlson Comorbidity Index**,** median (range)**	3 (0–6)
**ECOG Performance Status**	
0	38
1	2
Tumour size* (n)	
T1	6
T2	5
T3a	17
T3b	11
T4	1
**Risk Groups (D’Amico)**	
Intermediate	3
High	37
EBRT Data**	
Total dose (Gy)/Fr	
78 Gy/39 Fr, Prostate cancer	40
56 Gy/39 Fr, Elective volume	39
**Median**,** range**	
Overlap (PTV and BowelBag) (cc)	161 (0–282)
Overlap (PTV and AnoRectum) (cc)	4 (0–18)
PTV1 (cc)	135 (71–310)
PTV2 (cc)	758 (0–1227)
Rectum (cc)	88 (38–151)
Rectum *V*50 (%)	22.4 (4.8–49.0)
Rectum *V*60 (%)	13.8 (2.7–34.6)
Rectum *V*70 (%)	8.7 (1.3–19.4)
BowelBag *V*45 (cc)	310.7 (0–519.0)

*UICC (Union for International Cancer Control) TNM classification of malignant tumours, 8th edition

**Intended to treat data

Abbreviations: BMI, body mass index; ECOG, eastern cooperative oncology group; EBRT, external beam radiation therapy; Gy, Gray (unit of radiation dose); Fr: fractions (in radiation therapy); cc, cubic centimetre; PTV, planning target volume; PTV1, primary planning target volume; PTV2: secondary planning target volume; Rectum *V*50, volume of rectum receiving 50% of the radiation dose; Rectum *V*60, volume of rectum receiving 60% of the radiation dose; Rectum *V*70: volume of rectum receiving 70% of the radiation dose; TNM, tumour, nodes, metastasis

### Patient-reported acute RID by PRO stool diary

**Fig. 2:**
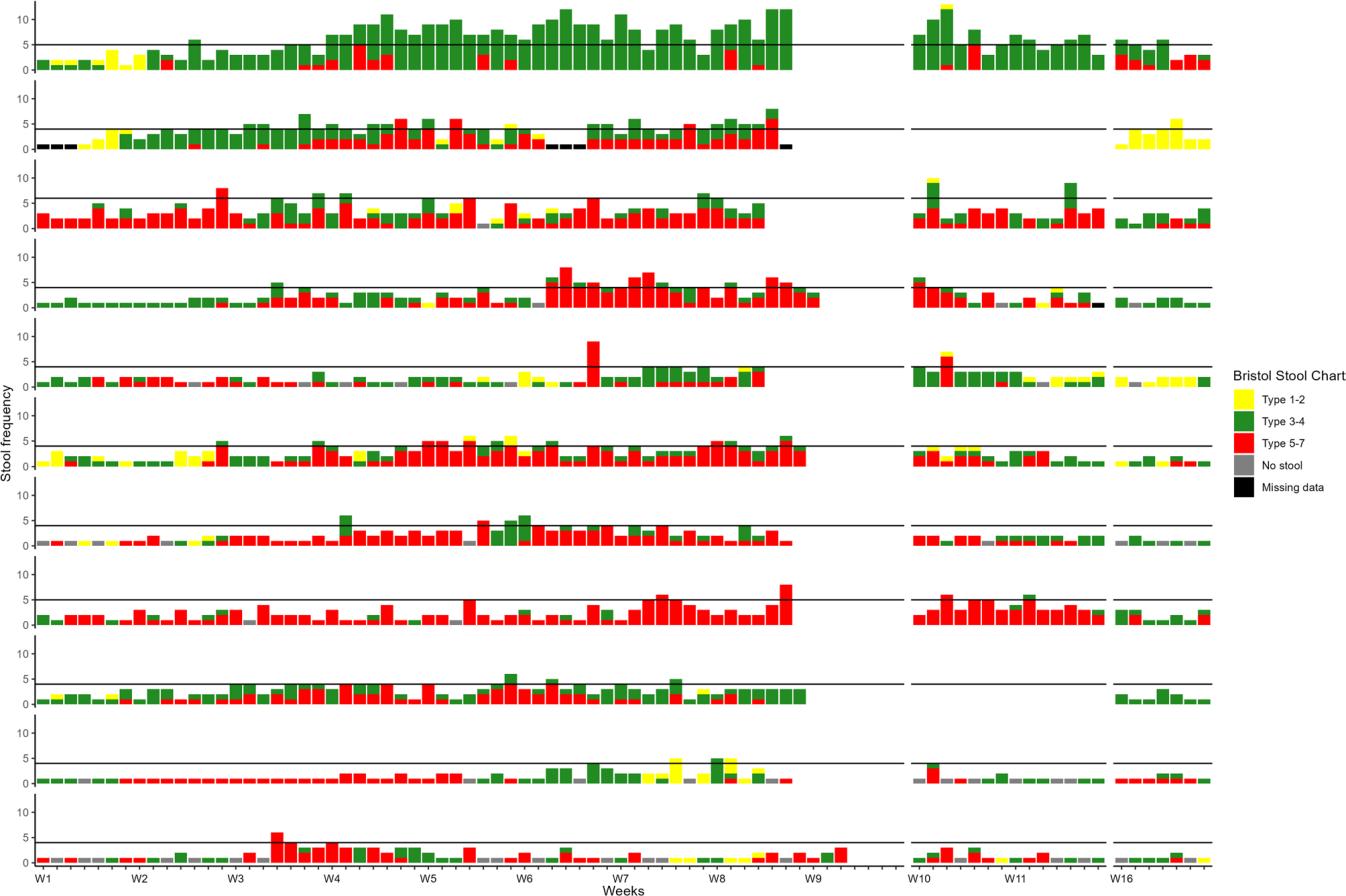
Frequency of RID for the 11 patients with Grade 2/3 according to Bristol Stool Chart. Bristol Stool Chart, Type 1–2: Yellow; Type 3–4: Green; Type 5–7: Red; No stool: Grey, Missing data: Black. The figure is synchronised on the 1st day of EBRT. The length of EBRT varies, as a total dose of 78 Gy was administered in daily fractions of 2 Gy from Monday to Friday, with breaks on weekends and public holidays. Thus, the entire EBRT period lasted 8–9 weeks. Time points for PROM data: Daily during EBRT, during two weeks after end of EBRT (week 8 or 9) and during one week 8 weeks after end of EBRT (week 16 or 17. The horizontal line symbolizes a frequency of stools ≥ 4 over baseline. The patients are sorted top-down: Patients with acute RID Grade 2/3 according to time of onset and duration. Abbreviation: W, Week

Figure 2 displays the frequency of acute RID for the 11 patients classified with Grade 2/3, as reported using the BSC. This figure also shows the few instances of missing data.

Ten out of eleven patients reported both Bristol stool types 3–4 and 5–7. Patient 1 exhibited an unusual pattern, primarily of stool types 3–4. This patient had two stools at baseline, and 6–12 stools at weeks 4–8, 10, and 16, corresponding to RID Grade 2/3.

The BSC for the 29 patients with Grade 0/1 is shown in Supplement 2.

### Clinician-reported acute RID by CTCAE scale

**Fig. 3:**
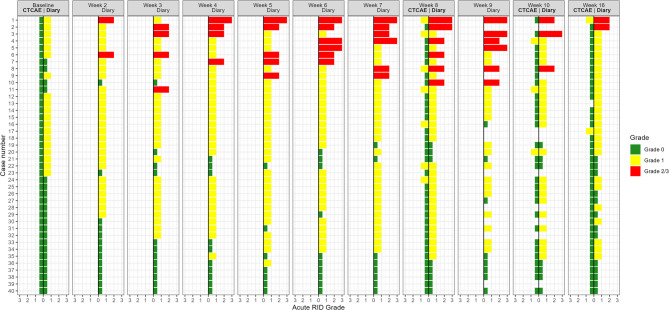
Comparison of acute RID by CTCAE and PROM diary data for all 40 patients. The time points were at baseline (week 1), at end of EBRT (week 8 or 9), 2 weeks after end of EBRT (week 10 or 11), and 8 weeks after end of EBRT (week 16 or 17). The results of all 40 patients are shown sorted top down by the grades from the PROM diary. There is missing data at week 10 for the first nine included patients. Assessments at baseline were made approximately 1 month before the start of EBRT with CTCAE and the first day of EBRT with PROM diary. Grade 0: Green; Grade 1: Yellow; Grade 2 or 3: Red. The patients are sorted top-down: Patients with Grade 3 according to duration and onset (case number 1–5), patients with Grade 2 (case number 6–11), patients with Grade 1 (case 12–36), and patients with Grade 0 (case number 37–40). Acute RID Grade 0: Green; Grade 1: Yellow; Grade 2/3: Red

At baseline, all 40 patients were assessed without diarrhoea. None of the patients progressed to Grade 2/3 at end of EBRT (week 8 or 9), 2 weeks after and of EBRT (week 10 or 11), or 8 weeks after end of EBRT (week 16 or 17) (Fig. 3). Apart from one patient hospitalised in week 16, there were no missing data.

### Comparison of acute RID by CTCAE and PRO-stool diary

Acute RID grading of all 40 patients is illustrated in Fig. 3. At baseline, the CTCAE assessed all patients as not having diarrhoea, while PRO data showed 19 patients with Grade 1. At end of EBRT (week 8 or 9), the CTCAE and PRO stool diary assessments were 33 vs. 9 for Grade 0, 7 vs. 25 for Grade 1, 0 vs. 4 for Grade 2, and 0 vs. 2 for Grade 3, respectively. Two weeks after end of EBRT (week 10 or 11), the clinician and the PRO stool diary assessed 29 vs. 7 for Grade 0, 3 vs. 21 for Grade 1, 0 vs. 2 for Grade 2, and 0 vs. 1 for Grade 3, respectively. Eight weeks after end of EBRT (week 16 or 17), the clinician and PRO stool diary assessments were 37 vs. 13 for Grade 0, 2 vs. 25 for Grade 1, 0 vs. 2 for Grade 2, and 0 vs. 0 for Grade 3, respectively. All patients graded 1 by CTCAE were graded ≥ 1 by the PRO stool diary.

### Twenty-four-hour registration of stool frequency by PRO stool diary

This registration involved all patients. Among patients with RID Grade 2/3, 7 of 11 experienced at least seven nights with ≥ 2 stools during and post-EBRT (Supplement 3).

24-hour registration of stool frequency by PRO stool diary for the 29 patients with acute RID Grade 0/1 is shown in Supplement 4.

### Urgency and faecal incontinence

At end of EBRT 10 out of 11 patients with RID Grade 2/3 indicated the categories 2 (a little) and 3 (quite a bit) of urgency and faecal incontinence (question 44). These symptoms were normalised 8 weeks after end of EBRT. (Supplement 5).

### Changes in body weight from baseline to weeks 8 and 16

**Fig. 4:**
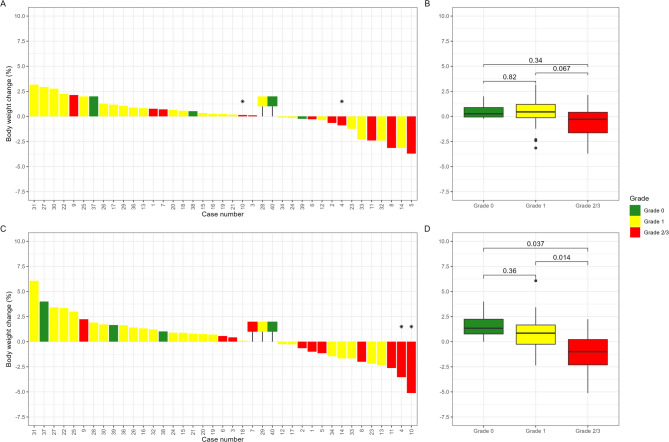
Changes in body weight from baseline (week 1) to end of EBRT (weeks 8 or 9) and 8 weeks after end of EBRT (week 16 or 17). *The two patients with weight loss > 2.5% in week 16 or 17, had both a weight loss < 2.5% in week 8 or 9. 39/40 patients are shown in the figure, one patient is omitted from the presentation due to a doctor-prescribed weight loss. RID Grade 0: Green; Grade 1: Yellow; Grade 2 or 3: Red. The coloured boxes upon the baseline are patients with stable weight

The body weight changes of 39 of 40 patients from baseline to end of EBRT (week 8 or 9) are shown in Fig. 4A and B, and from baseline to 8 weeks after end of EBRT (week 16 or 17) in Fig. 4C, and Fig. 4D. One patient was omitted owing to a doctor-prescribed weight loss. At end of EBRT (week 8 or 9), 14 of 39 patients experienced weight loss, 2 had stable weight, and 23 gained weight. Three patients experienced weight loss > 2.5%, while none lost weight > 5%. At 8 weeks after end of EBRT (week 16 or 17), 14 of 39 patients experienced weight loss, 3 had stable weight, and 22 gained weight. The two patients* with weight loss > 2.5% and 5% at 8 weeks after end of EBRT (week 16 or 17), respectively had both experienced a weight loss < 2.5% at week 8. One patient experienced weight loss of 5%. Significant differences in weight loss between the patients with RID Grade 1 vs. Grade 2/3 (*p* = 0.067) from baseline to end of EBRT (week 8 or 9), and the patients with RID Grade 0 vs. Grade 2/3 (*p* = 0.037), and Grade 1 vs. Grade 2/3 (*p* = 0.014) from baseline to 8 weeks after end of EBRT (week 16 or 17) were observed.

### Medications

Overall, 12 of 40 patients used an antidiarrheal medication and/or laxatives during EBRT. Several patients only reported that they took antidiarrheal medication and/or laxatives, not the dose and duration. Thus, these data could not be calculated.

### The impact of EBRT data on RID

There was no statistical correlation between the irradiation data (*V*50, *V*60, *V*70, and BowelBag *V*45) and the 11 patients with RID Grade 2/3 vs. the 29 patients with RID Grade 0/1, respectively (Supplement 6).

## Discussion

ClinRO-CTCAE and PRO data were completed for and by the 40 patients, justifying the use of a PRO stool diary that includes the Bristol Stool Chart despite the patient burden of filling in data.

### Comparison of CTCAE and PRO stool diary data

This study demonstrates the necessity of a PRO stool diary during and after EBRT to gather information about acute RID. Regardless of the Bristol stool type, the frequency of stools per day appears to be the most crucial factor for RID, which is in line with earlier results claiming that Bristol stool type 2, 3, 5, and 6 needs improving validity and reliability [18].

Initially, half of the patients reported Grade 1 diarrhoea, potentially caused by three months of ADT [25].

Throughout the study, acute RID Grade 2/3 was documented in 11 out of 40 patients via PRO stool diary but not with ClinRO-CTCAE. According to the results of this study all patients should have been clinically monitored for acute RID starting from week 2. Most patients reported acute RID at the end of EBRT, persisting for the following 2 weeks and improving 8 weeks after end of EBRT as previously reported [26]. Two recent studies in patients with prostate cancer corroborate our findings: one study found that RID increased by 19% at the end of EBRT compared to baseline [13]. Another study, involving patients with prostate cancer treated with proton beam therapy, compared the incidence of acute RID using investigator-reported (IR) CTCAE and PRO-CTCAE. IR-CTCAE underestimated RID compared to PRO-CTCAE at the end of treatment, showing 28% underestimation of toxicity Grade ≥ 1 and 11% for Grade ≥ 2 [27]. The PRO-CTCAE tool appears more effective than classical CTCAE in reporting acute RID. However, ClinRO-CTCAE assess the adverse events as weekly averages, and the RID frequency is reported as Grade 1–4, whereas the PRO stool diary report the daily RID frequency and lead to a more appropriate diagnosis of acute RID.

### Acute RID at nighttime

Higher frequencies of acute RID during the day often correlate with increased nighttime episodes, which can significantly disrupt patient rest if occurring more than twice per night. Reporting the time of day for these episodes could help clinicians identify patients who experience particularly troublesome nocturnal symptoms, enabling more targeted interventions.

### Changes in body weight

The patients with acute RID Grade 2/3 showed significant body weight loss compared with patients with RID Grade 1 at week 8 (end of treatment) (>2.5%). Based on these results, it is recommended that all patients with prostate cancer undergoing curative EBRT with RID monitor their weight weekly. In contrast, those who showed weight gain at end of EBRT tended to gain even more at 8 weeks after end of EBRT, likely owing to the effects of ADT [28].

### The impact of EBRT data on RID

As previously reported, radiation exposure to the small bowel (BowelBag) is associated with an increased risk of acute RID. Modern radiation therapy techniques (3DCRT or IMRT) compared to conventional RT significantly reduce the incidence of acute RID [29]. In our study, we utilised modern IMRT, and the BowelBag data did not correlate with the PRO stool diary findings of acute RID Grade ≥ 2. This discrepancy may be attributed to the method used to delineate the small intestine volume within the irradiated field, which warrants further investigation.

### Medication

The intake of antidiarrheal medication and laxatives may influence the grade of RID. As shown by Lawrie et al., the effect of pharmacologic interventions on acute RID is unclear [29]. The information on the intake of any medication in the PRO stool diary was uncertain. Therefore, the PRO stool diary should be improved with registration of the daily dose, duration, and patient adherence.

### Strengths and limitations

The strength of this study is the collection of data from a homogeneous patient cohort with only one diagnosis and curative tumour stage. All the patients were treated with standard Danish EBRT. Patient compliance was high. Overall, there were few missing data, except on data for concomitant medication. A major strength of this study is its prospective design and the use of validated, internationally recognised patient-reported tools as BSC and CTCAE. Nevertheless, several methodological limitations should be acknowledged. The relatively small sample size increases the risk of both type I and type II errors. Taken together, the study offers valuable insights into the diagnostic challenges of acute RID with the ClinRO-CTCAE and the PRO stool diary.

## Conclusions

One in four patients with prostate cancer undergoing curative EBRT reported acute RID Grade 2/3 via PRO, but none with ClinRO-CTCAE. Using the PRO stool diary, the onset and course of acute RID, as well as body weight changes, can be easily diagnosed and monitored. The exclusive use of CTCAE at few measure points during EBRT is insufficient and should be supplemented with a daily PRO stool diary.

## Supplementary Information

Below is the link to the electronic supplementary material.

Supplementary Material 1

Supplementary Material 2

Supplementary Material 3

Supplementary Material 4

Supplementary Material 5

Supplementary Material 6

## Data Availability

Danish law makes the datasets generated and/or analysed during the current study publicly available. All research data are stored in REDCap, Aalborg University Hospital and can be made available by the corresponding author upon reasonable request at any time.

## Abbreviations

ADT: Androgen deprivation therapy
BMI: Body mass index
ClinRO: Clinician-reported outcome
CT: Computed tomography
CTCAE: Common Terminology Criteria for Adverse Events
EBRT: External beam radiation therapy
ECOG: Eastern cooperative oncology group
EORTC QLQ-C30: European Organisation for Research and Treatment of Cancer Quality of Questionnaire - Core 30
EORTC QLQ-PRT20: European Organisation for Research and Treatment of Cancer Quality of Life Questionnaire – Proctitis Module (PRT20)
IPAQ SF: International Physical Activity Questionnaire - Short Form
MRI: Magnetic resonance imaging
PG-SGA SF: Patient-Generated Subjective Global Assessment - Short Form; IPAQ SF
PRO: Patient-Reported Outcome
PROM: Patient-Reported Outcome Measure
PTV: Planning target volume
REDCap: Research Electronic Data Capture
RID: Acute radiation-induced diarrhoea
TPS: Treatment planning system
