# Supplementary material for: Patient- and clinician-reported acute radiation-induced diarrhoea in patients with prostate cancer during curative external radiation therapy: A prospective observational cohort study
Source: J Patient Rep Outcomes. 2025 Dec 24;10:15. doi: 10.1186/s41687-025-00957-3 (PMC12847486; doi:10.1186/s41687-025-00957-3)
Supplement: Supplementary file 1 — Supplementary Material 1 [file 41687_2025_957_MOESM1_ESM.docx]

**Supplement 1**

PROM diary

**PROM diary**

First and last name: ___________________________________

Personal identification number: ____________________


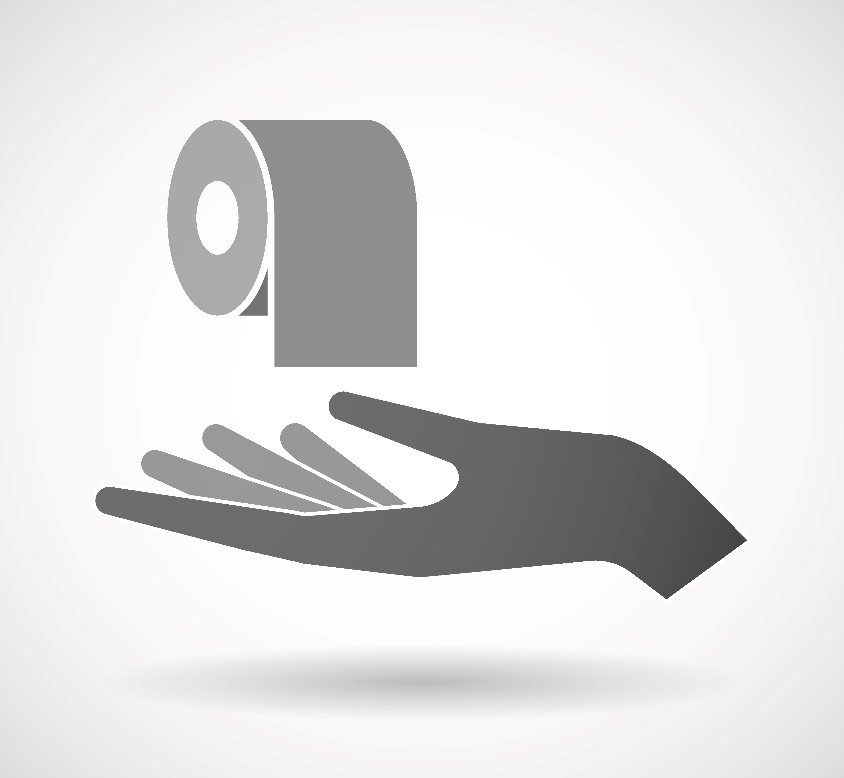


**Stool diary**

Please keep a diary of when and how many stools you pass daily, from

the first day of the radiotherapy until 2 weeks after the end of

radiotherapy. Repeat the stool diary daily for 7 days 8 weeks after

the end of radiotherapy. Categorise the type of each stool using the

the Bristol scale (type 1-7). Days without stools are marked with %.

Other observations can be noted in the remark field; i.e. between food and drink and the type of your stool, what you did, urgency, use of incontinence products, description of the food you ate before the stool, abnormal colour, bloody, foamy, floating on top of the toilet water or other you remarks.

In the remark field, please record your body weight weekly from day 1 of your radiotherapy. Weigh yourself in the morning, using light clothing, after toilet visit and prior to food and drinks. Please round up to the nearest 0.1 kg.

Please record any use (and dose) of antidiarrheics or laxatives such as

Imodium, HUSK, Movicol, and magnesia.

The completed stool diary is returned to the project contact person responsible using the self-addressed stamped envelope.

**Example**

| **Diary Bristol scale for stool types** | | | | | | | | **Comments** |
| --- | --- | --- | --- | --- | --- | --- | --- | --- |
| **Date** | **5/1-22** | **6/1-22** | **7/1-22** | **8/1-22** | **9/1-22** | **10/1-22** | **11/1-22** |  |
| **Body weight** | **76,8** **kg** |  |  |  |  |  |  |  |
| **Day of the week** | **Wednesday** | **Thursday** | **Friday** | **Saturday** | **Sunday** | **Monday** | **Tuesday** |  |
| 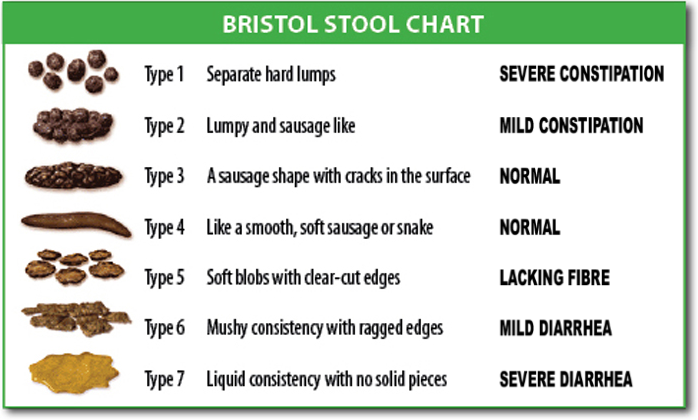  Type 1 Hard lumps | **10.30**  **21.00** |  |  |  | **%** |  |  | **Wednesday: Before bedtime Magnesia 2 x 500 mg** |
| 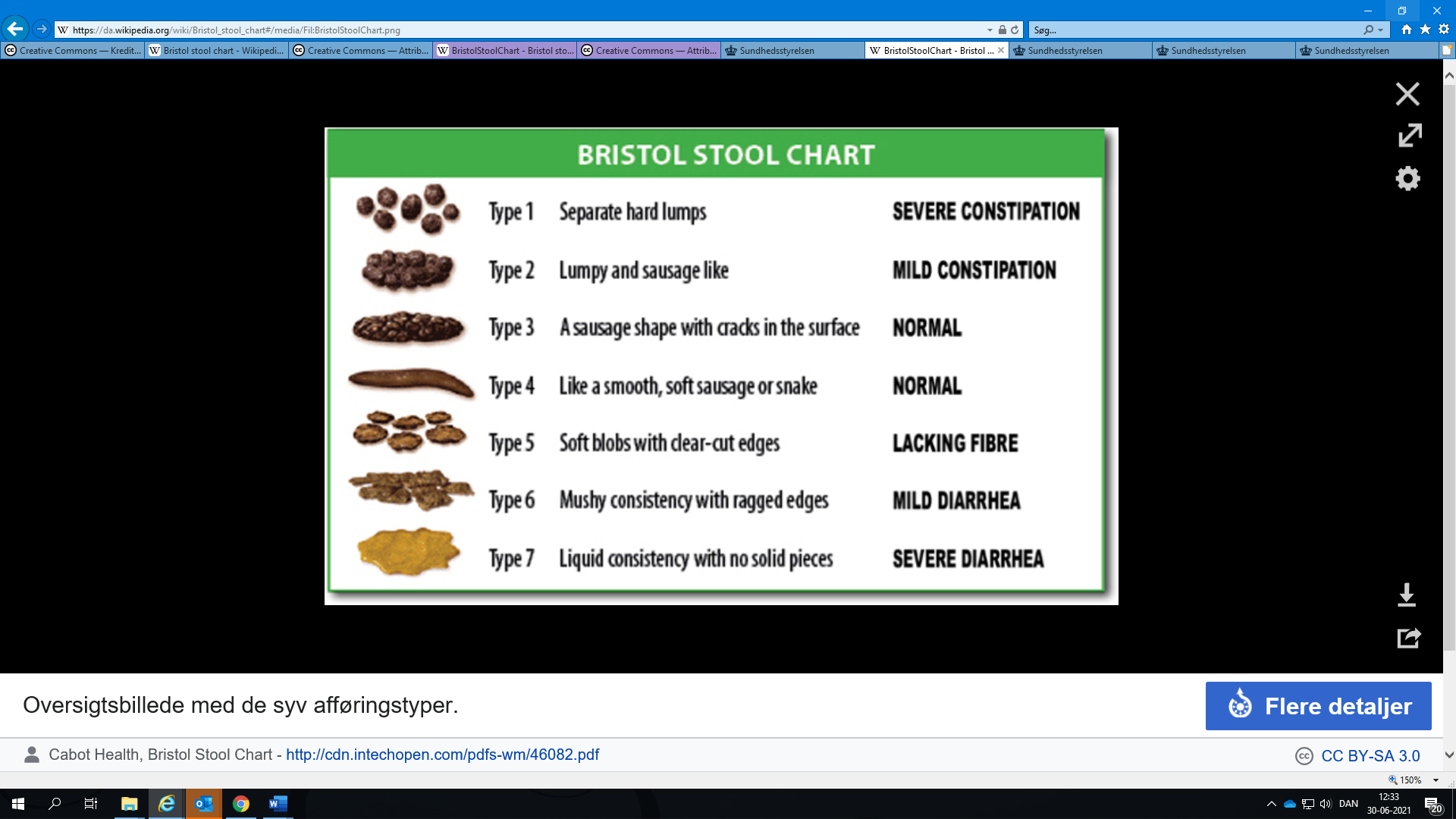  Type 2 Sausage-shaped  (lumpy surface) |  |  |  |  | **%** |  |  |  |
| 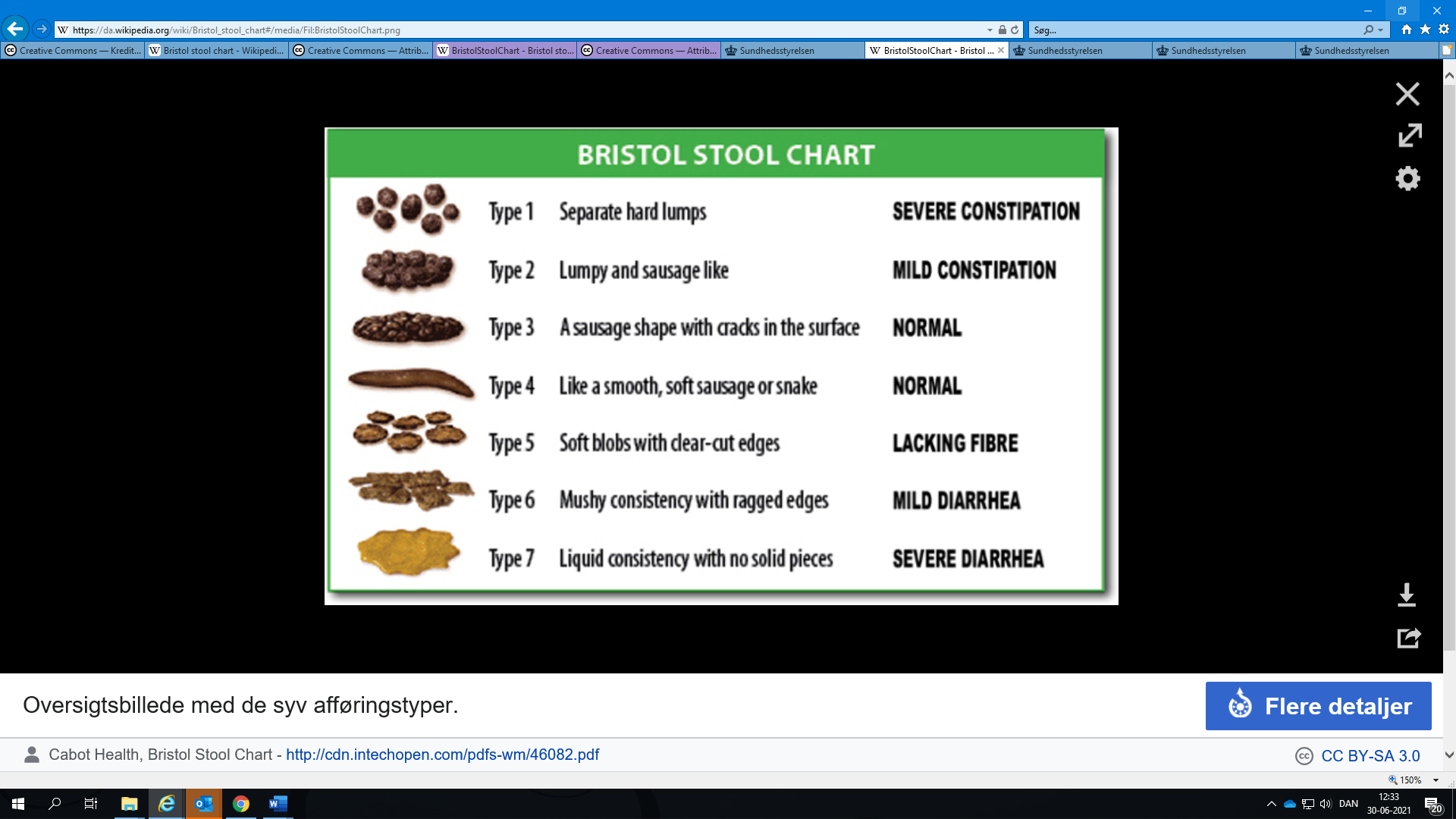  Type 3 Sausage-shaped  (cracked) |  |  | **12.30** |  | **%** |  |  |  |
| 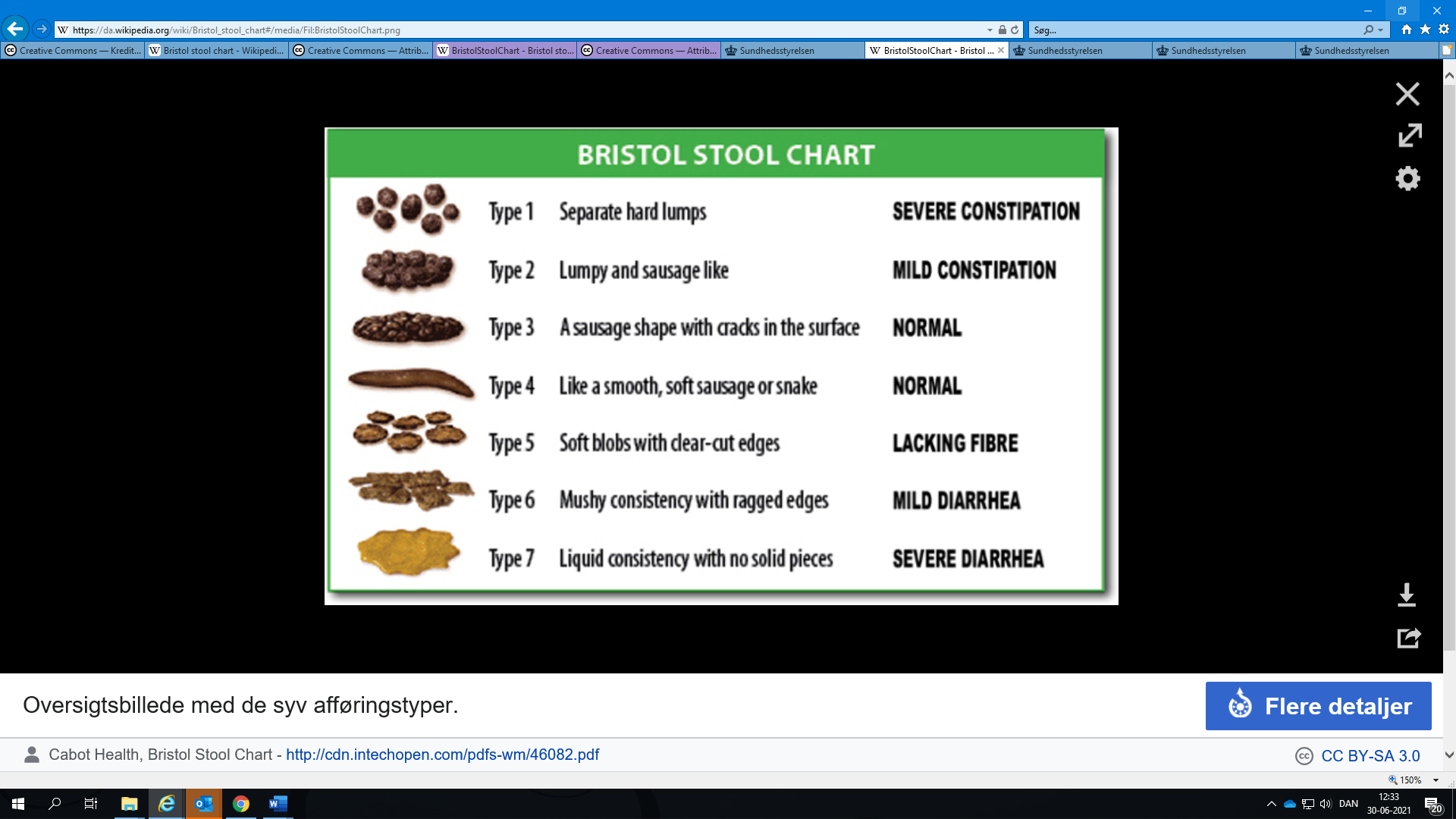  Type 4 Sausage or snake-shaped  (smooth and soft) |  | **09.00**  **14.00** |  | **8.30**  **14.00**  **21.45** | **%** | **08.00**  **10.30** | **07.00** |  |
| 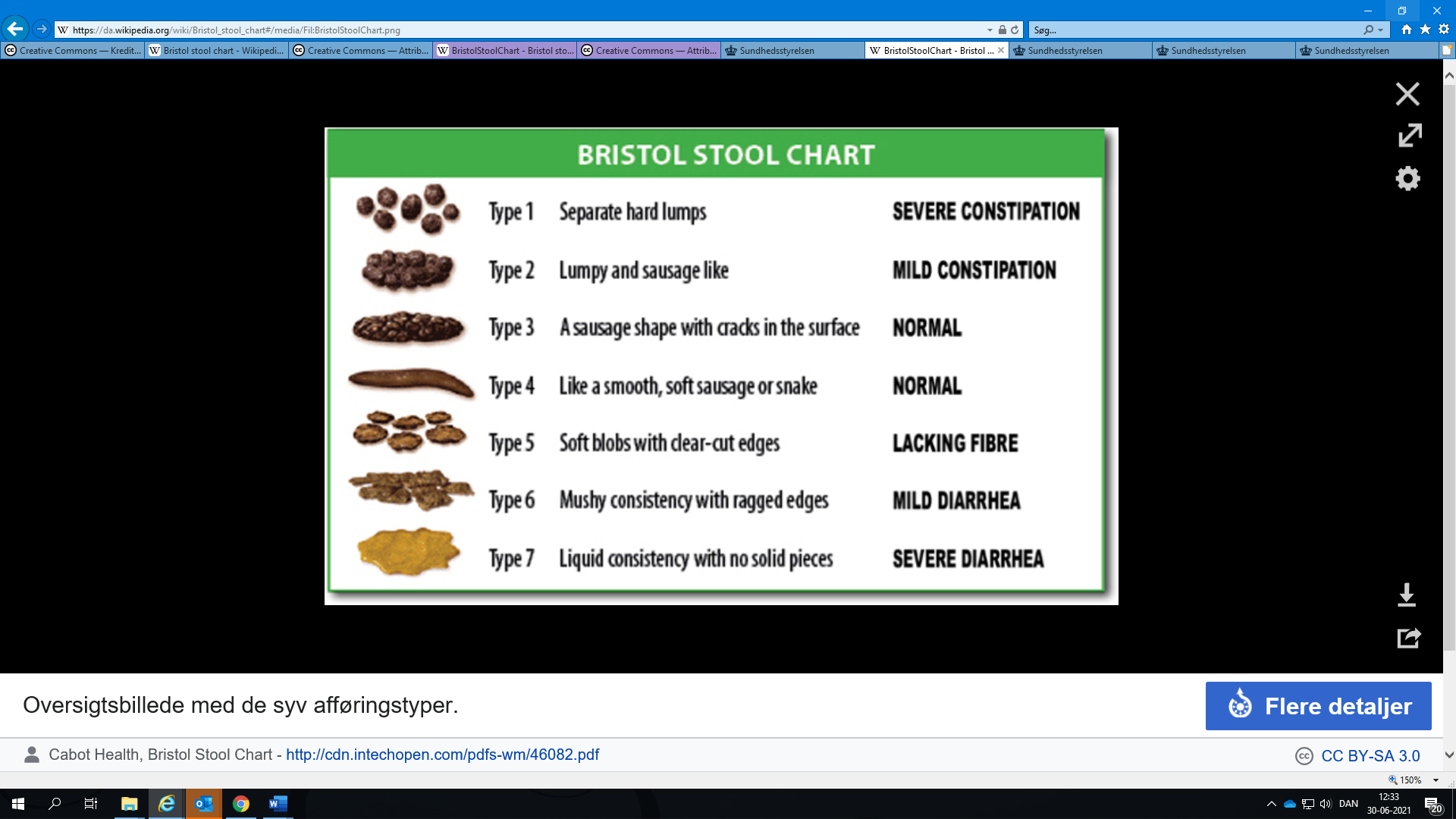  Type 5 Soft blobs  (clear cut edges) |  | **10.00**  **11.15**  **12.00** | **07.00** | **19.30**  **20.15** | **%** | **20.30** | **02.15**  **10.15** | **Thursday: 1½ hours after yoghurt for breakfast+2 measuring spoons HUSK, after the 3^rd^ loose stool Imodium tab x 2**  **Saturday: Dinner takeaway meal: 2 fish fillets and fries** |
| 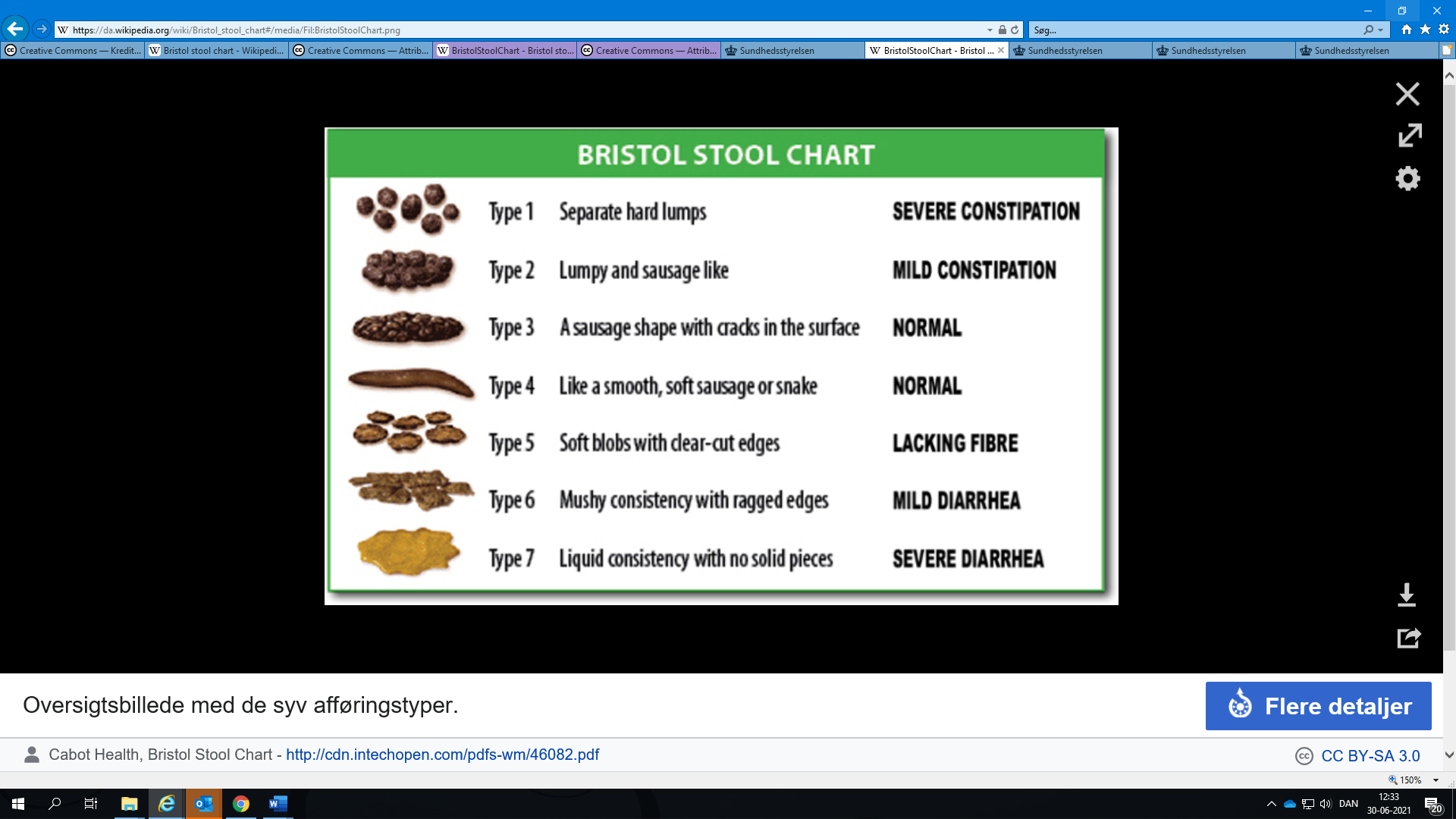 Mushy stool  Type 6 (fluffy small pieces,  ragged edged) | **16.00** | **10.45** |  |  | **%** | **21.15** | **01.30** | **Thursday: Imodium tab x 1**  **Monday: Imodium tab x 1**  **Tuesday: Difficult to flush stools, yellowish/foamy, Imodium tab x 1** |
| 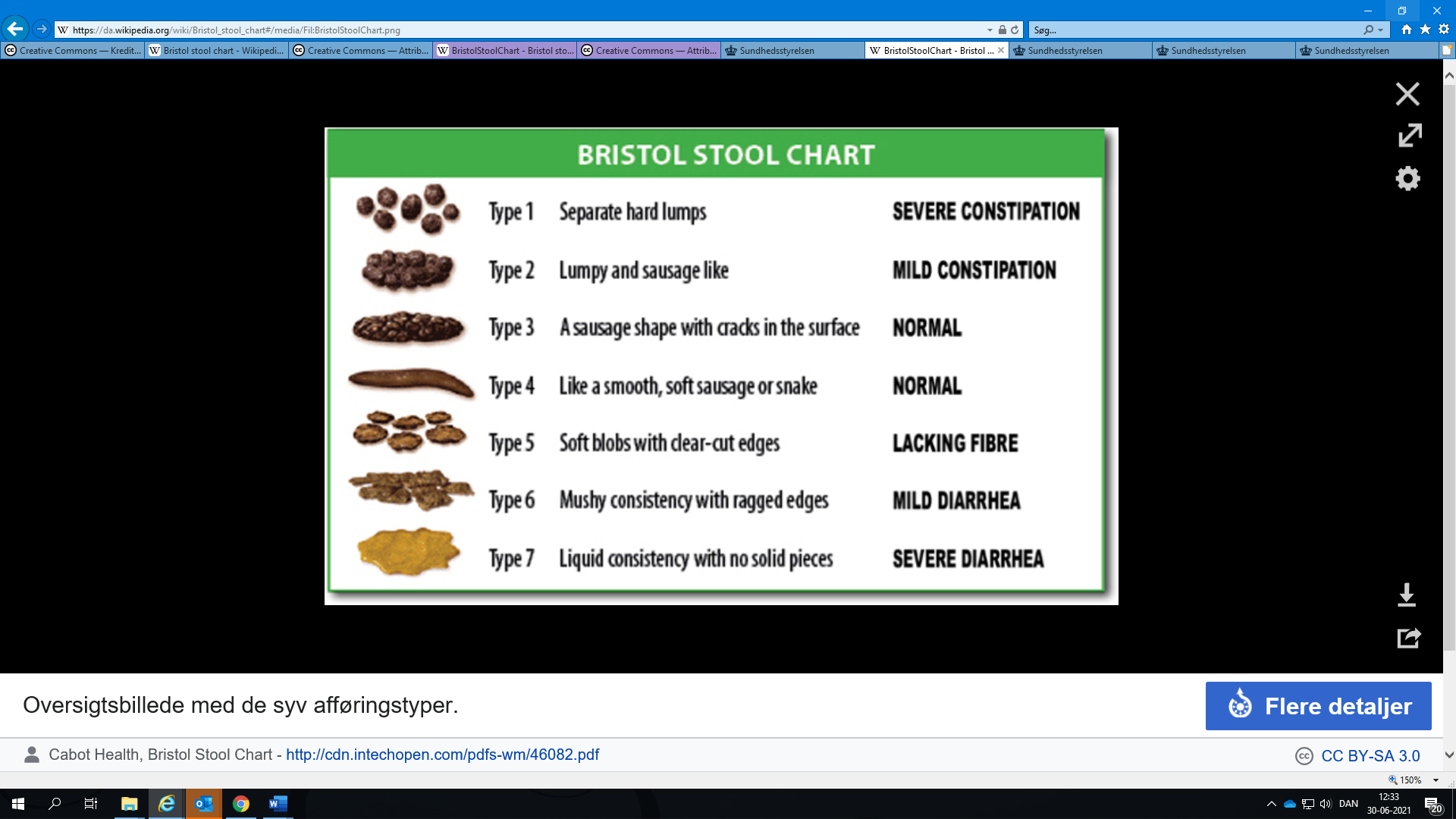  Type 7 Watery  (no solid pieces) | **13.30** |  |  |  | **%** | **22.30** | **00.15** | **Monday: Imodium tab x 1, 2 measuring spoons HUSK, diaper at bedtime**  **Tuesday: Imodium tab x 2, 2 measuring spoons HUSK, diaper at bedtime** |

*Modified from wikipedia, license: CC BY-SA 3.0*

**Week 1**

| **Diary Bristol scale for stool types** | | | | | | | | **Comments** |
| --- | --- | --- | --- | --- | --- | --- | --- | --- |
| **Date** |  |  |  |  |  |  |  |  |
| **Body weight** | **kg** |  |  |  |  |  |  |  |
| **Day of the week** | **day** | **day** | **day** | **day** | **day** | **day** | **day** |  |
| 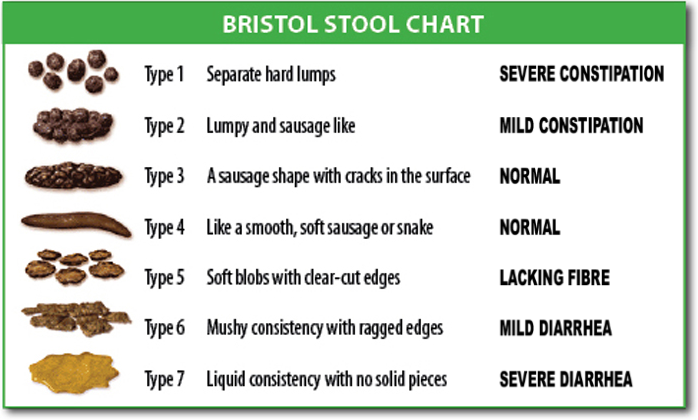  Type 1 Hard lumps |  |  |  |  |  |  |  |  |
| 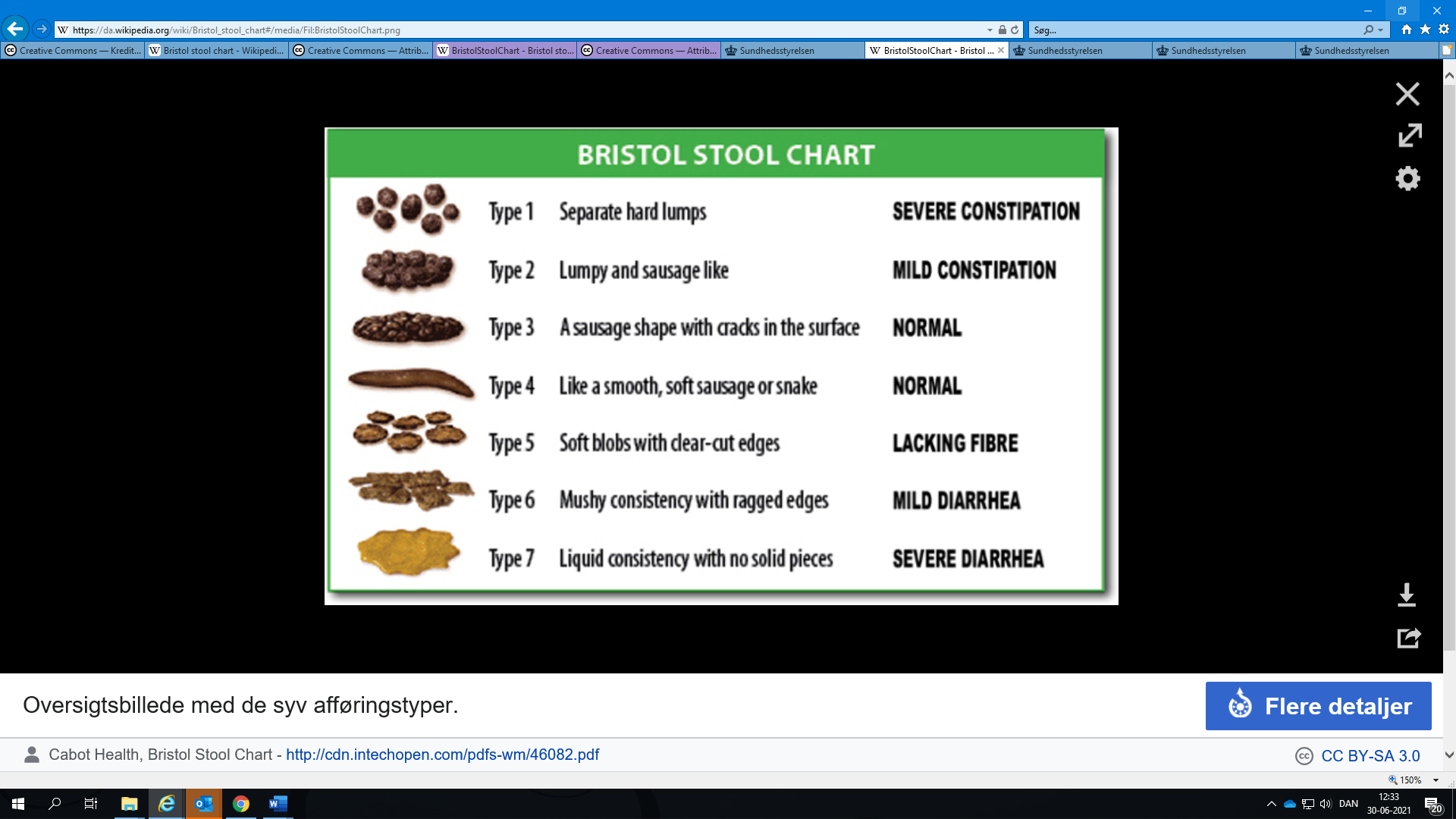  Type 2 Sausage-shaped  (lumpy surface) |  |  |  |  |  |  |  |  |
| 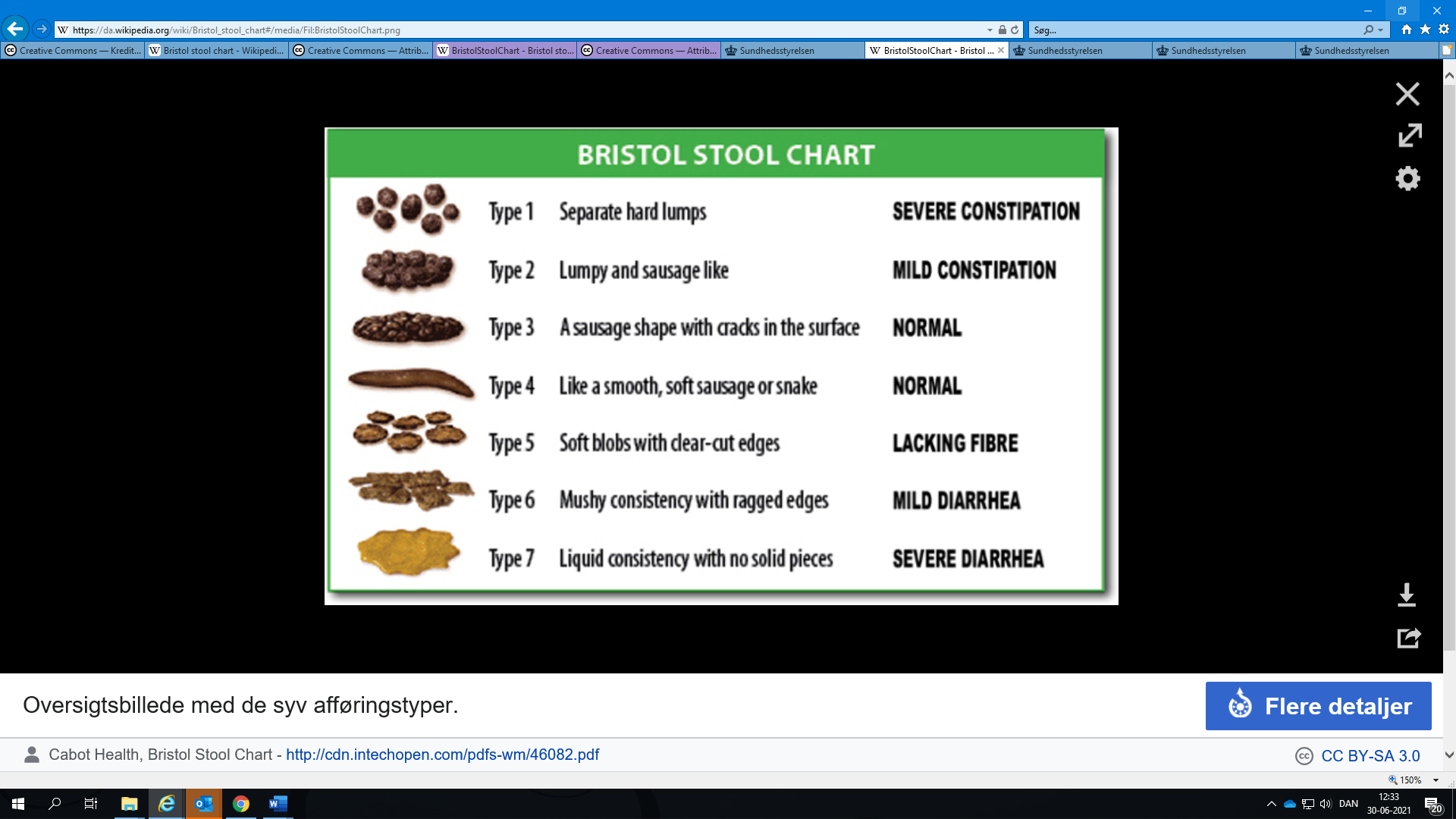  Type 3 Sausage-shaped  (cracked) |  |  |  |  |  |  |  |  |
| 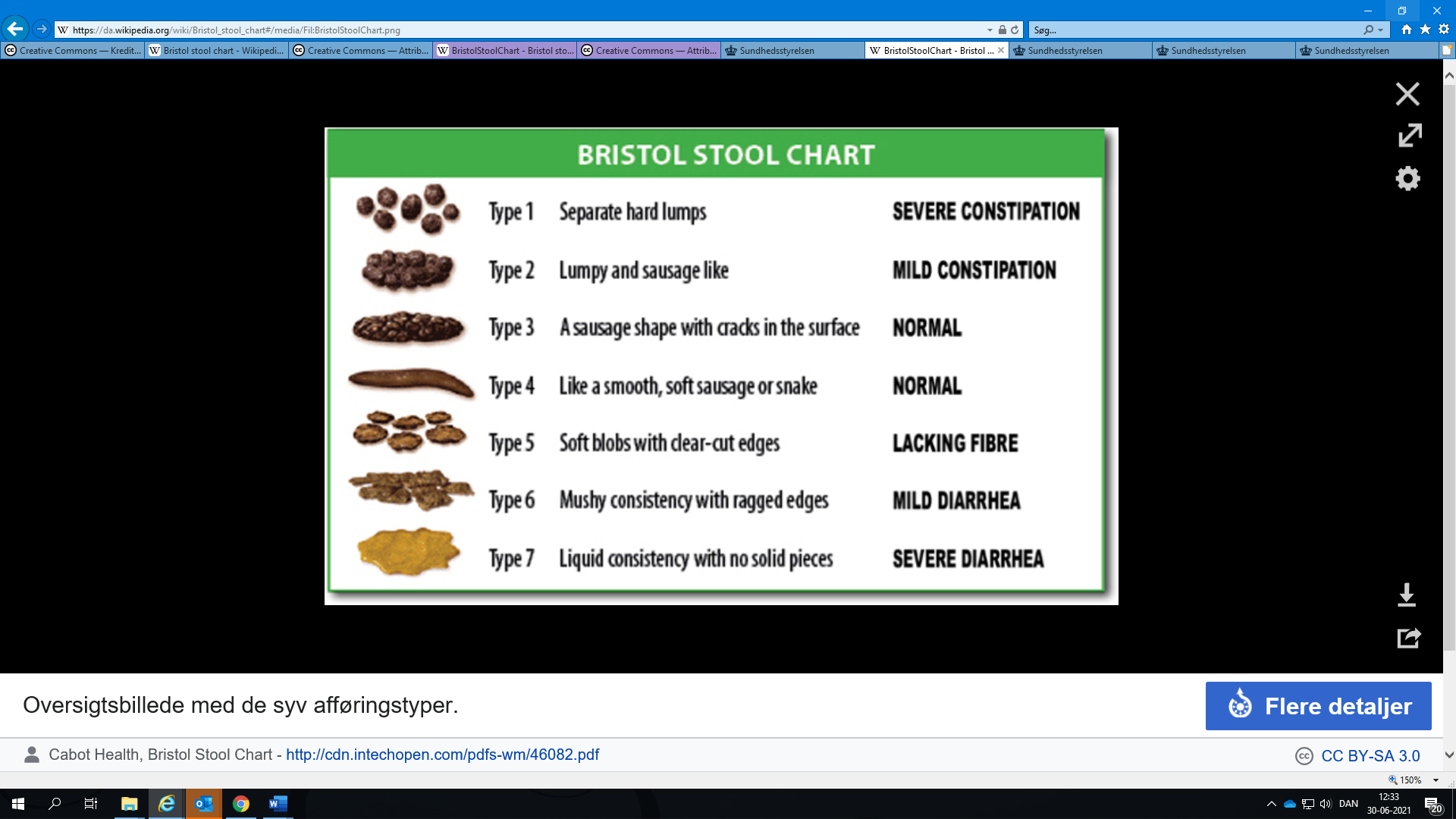  Type 4 Sausage or snake-shaped  (smooth and soft) |  |  |  |  |  |  |  |  |
| 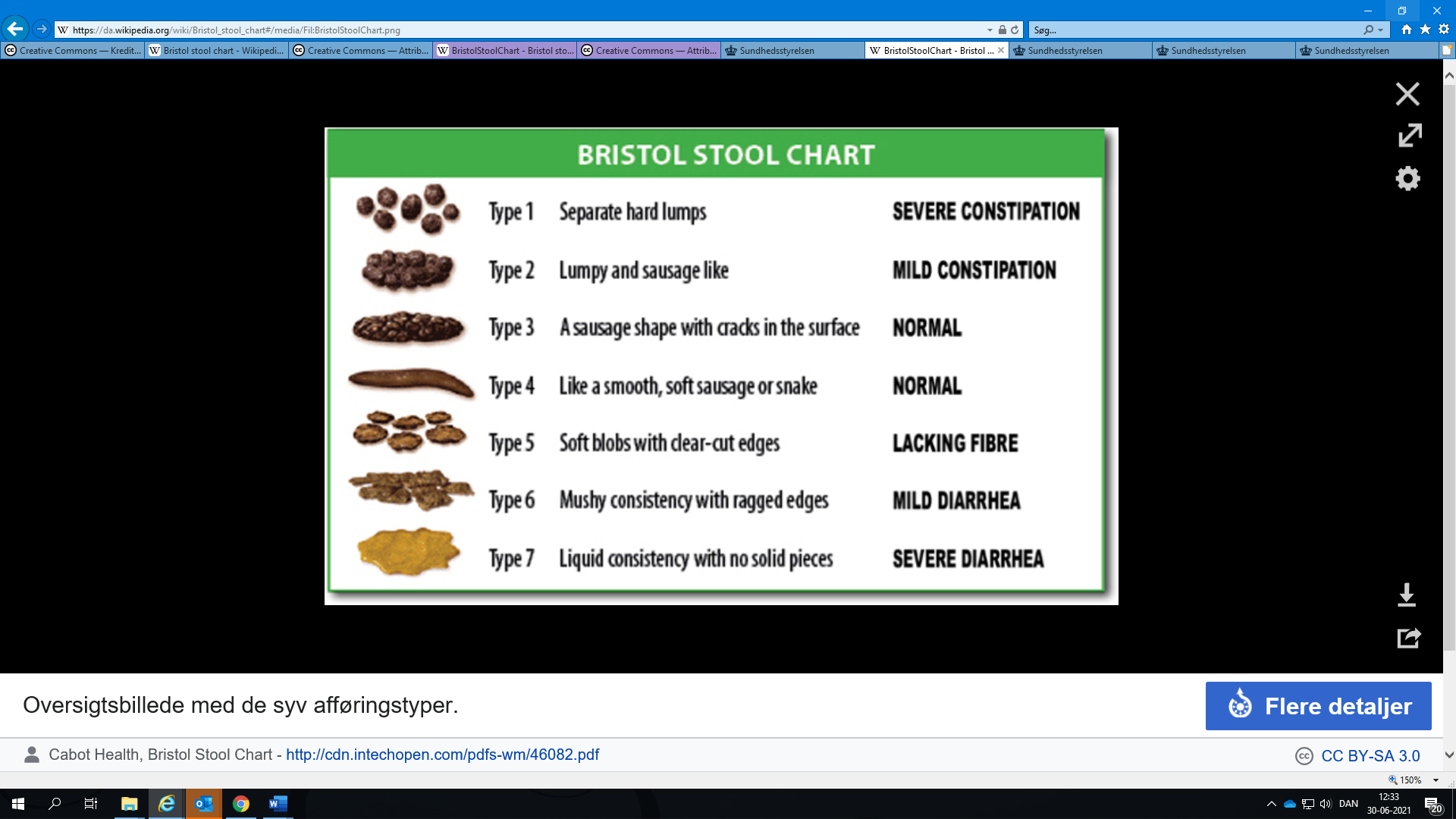  Type 5 Soft blobs  (clear cut edges) |  |  |  |  |  |  |  |  |
| 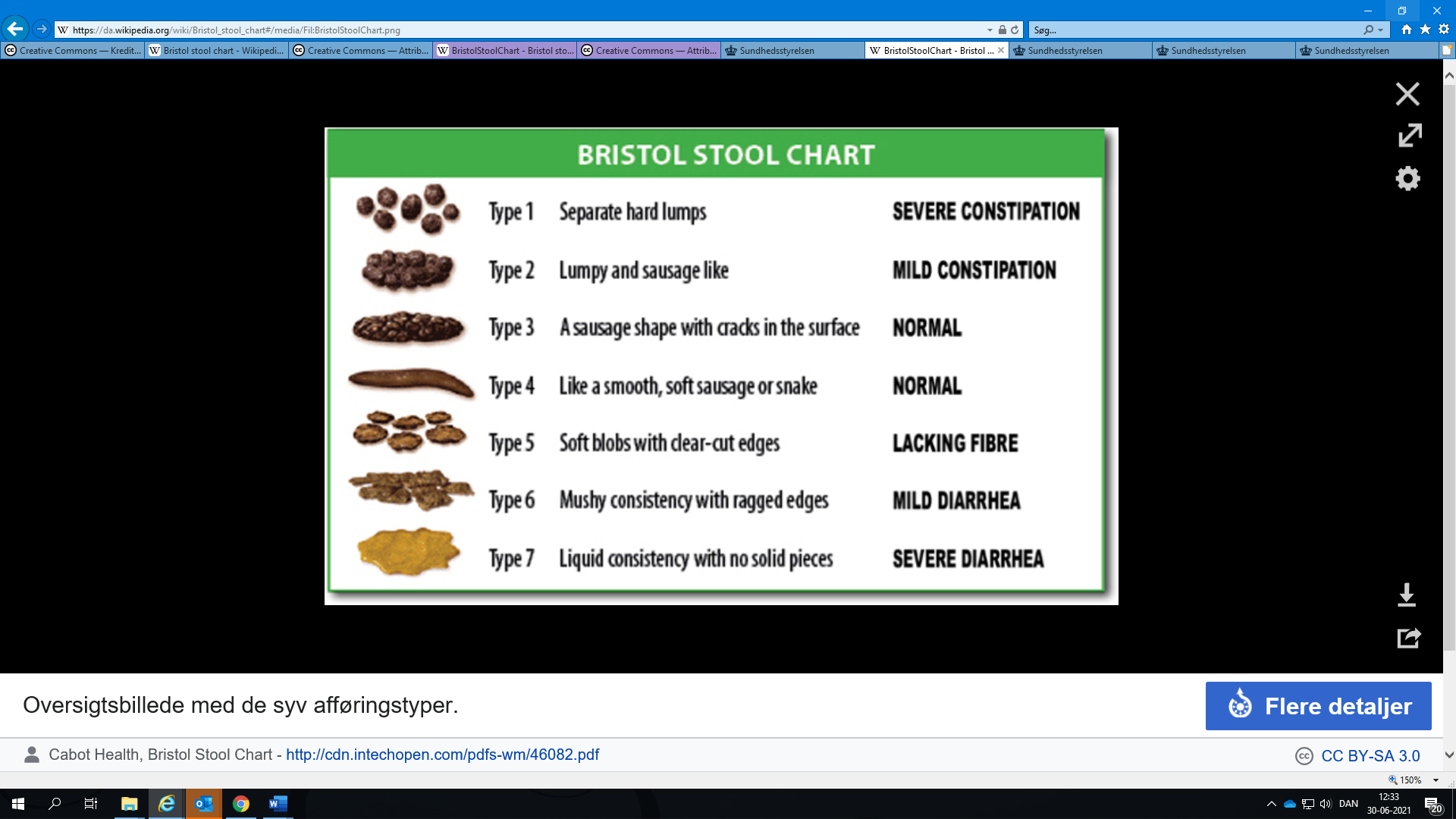 Mushy stool  Type 6 (fluffy small pieces,  ragged edged) |  |  |  |  |  |  |  |  |
| 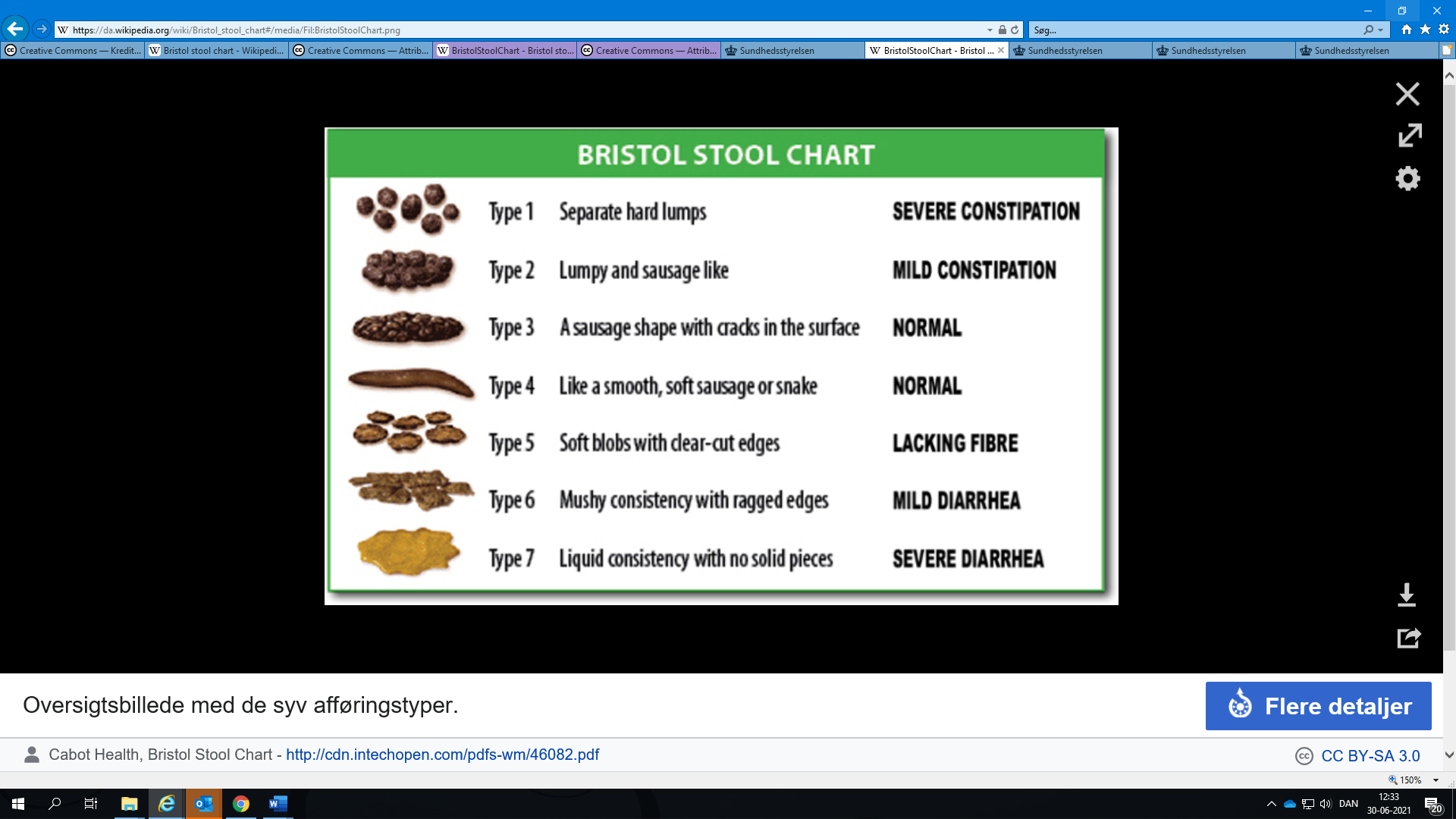  Type 7 Watery  (no solid pieces) |  |  |  |  |  |  |  |  |

**Week 2**

| **Diary Bristol scale for stool types** | | | | | | | | **Comments** |
| --- | --- | --- | --- | --- | --- | --- | --- | --- |
| **Date** |  |  |  |  |  |  |  |  |
| **Body weight** | **kg** |  |  |  |  |  |  |  |
| **Day of the week** | **day** | **day** | **day** | **day** | **day** | **day** | **day** |  |
| 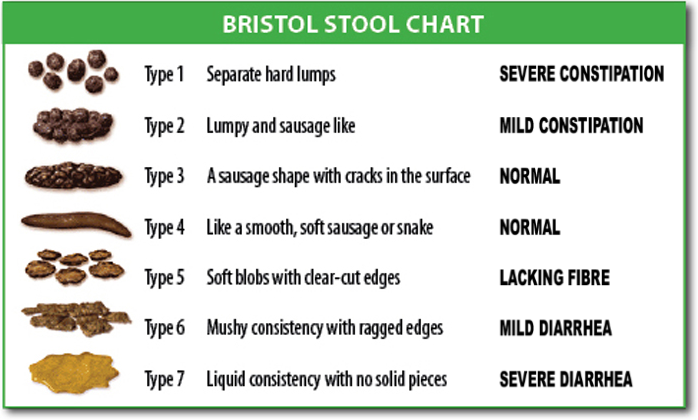  Type 1 Hard lumps |  |  |  |  |  |  |  |  |
| 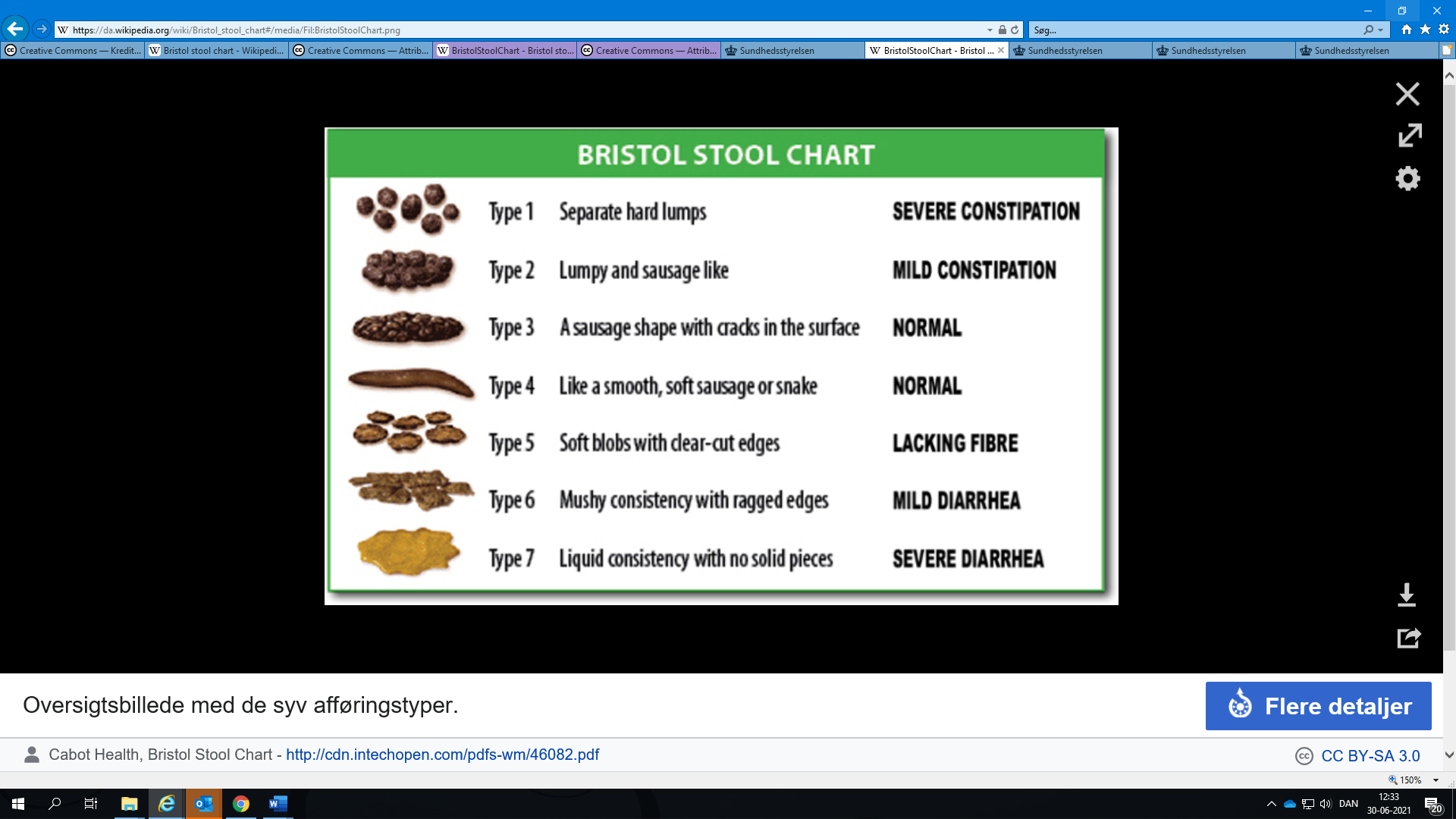  Type 2 Sausage-shaped  (lumpy surface) |  |  |  |  |  |  |  |  |
| 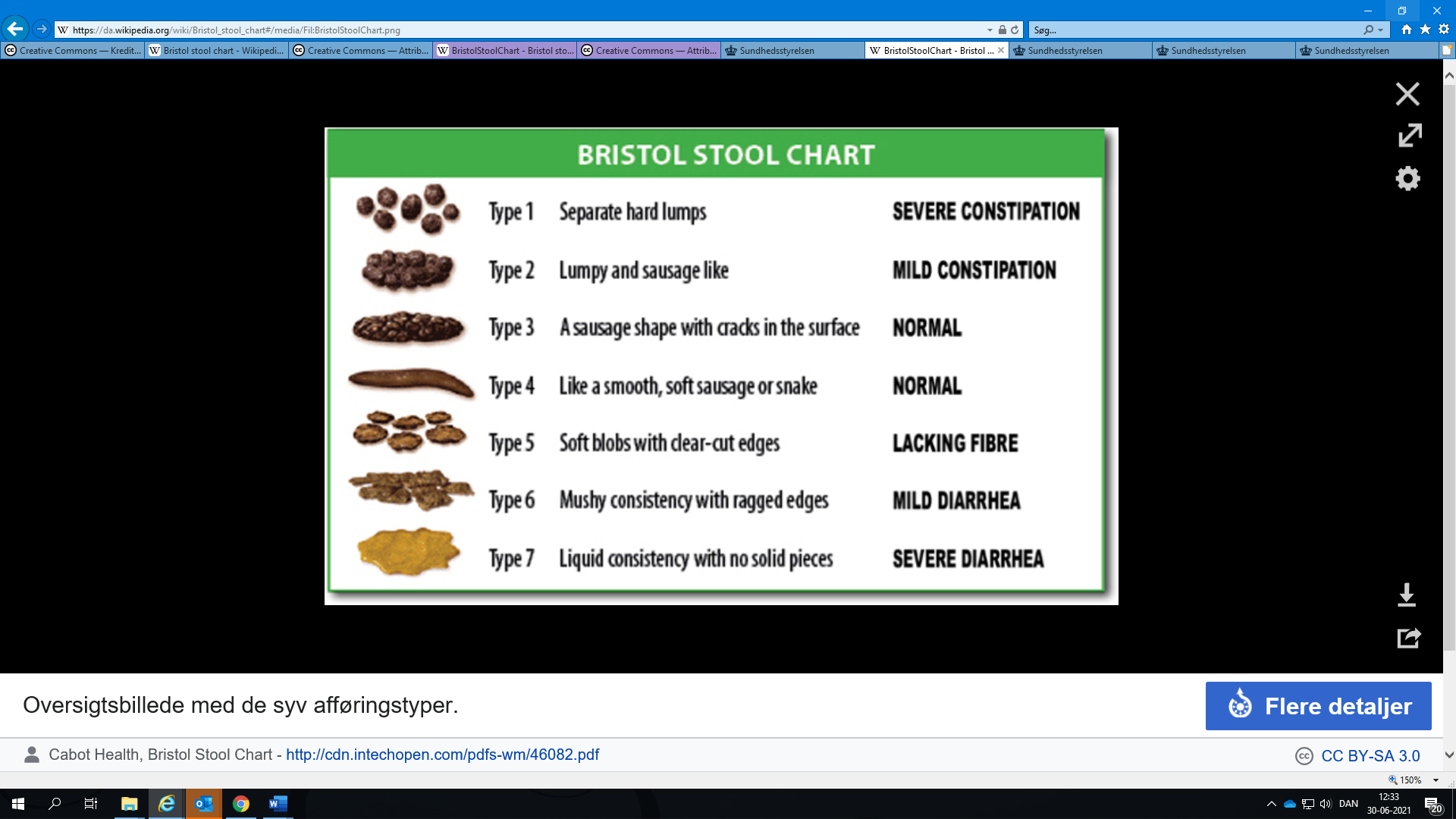  Type 3 Sausage-shaped  (cracked) |  |  |  |  |  |  |  |  |
| 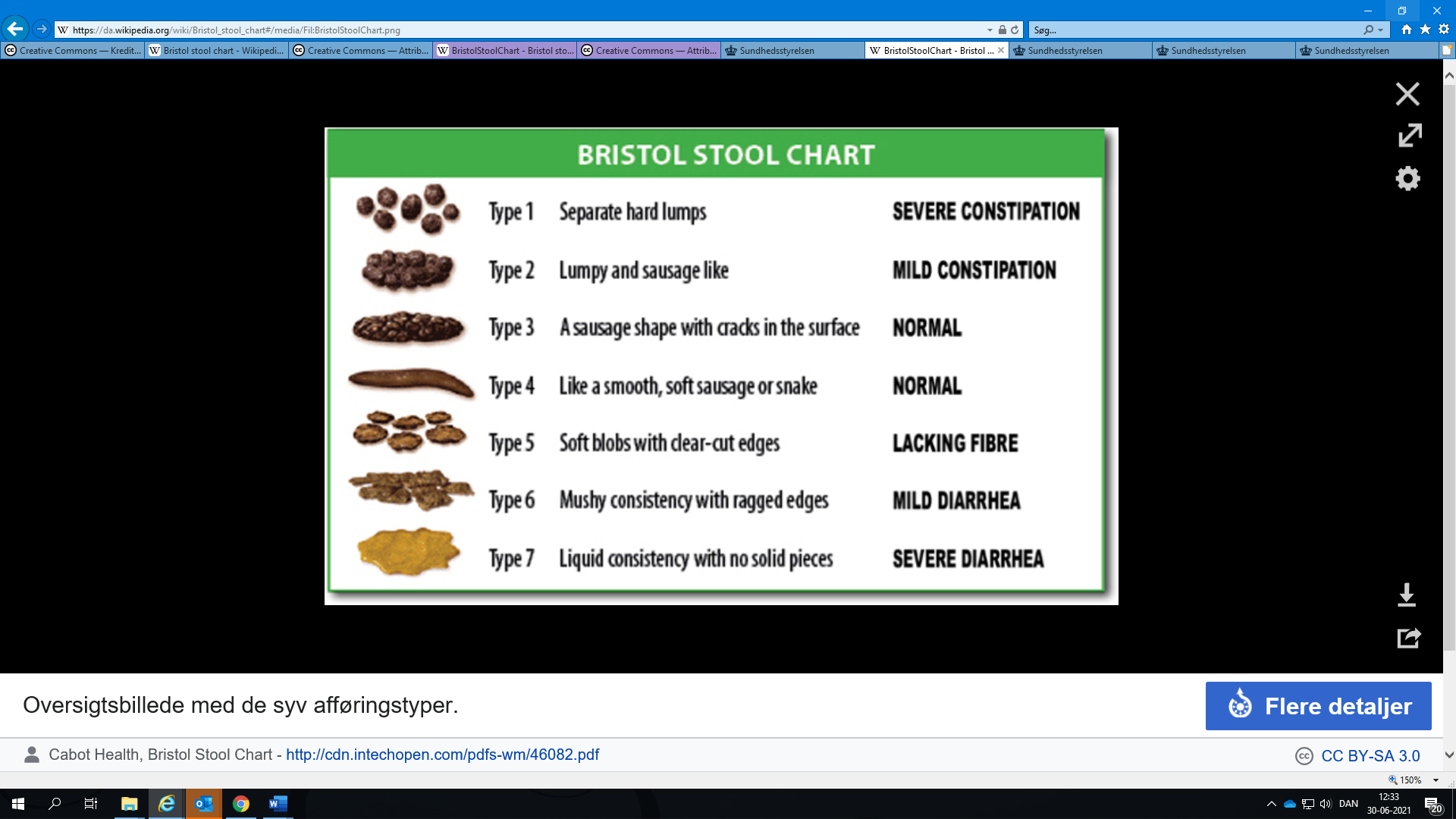  Type 4 Sausage or snake-shaped  (smooth and soft) |  |  |  |  |  |  |  |  |
| 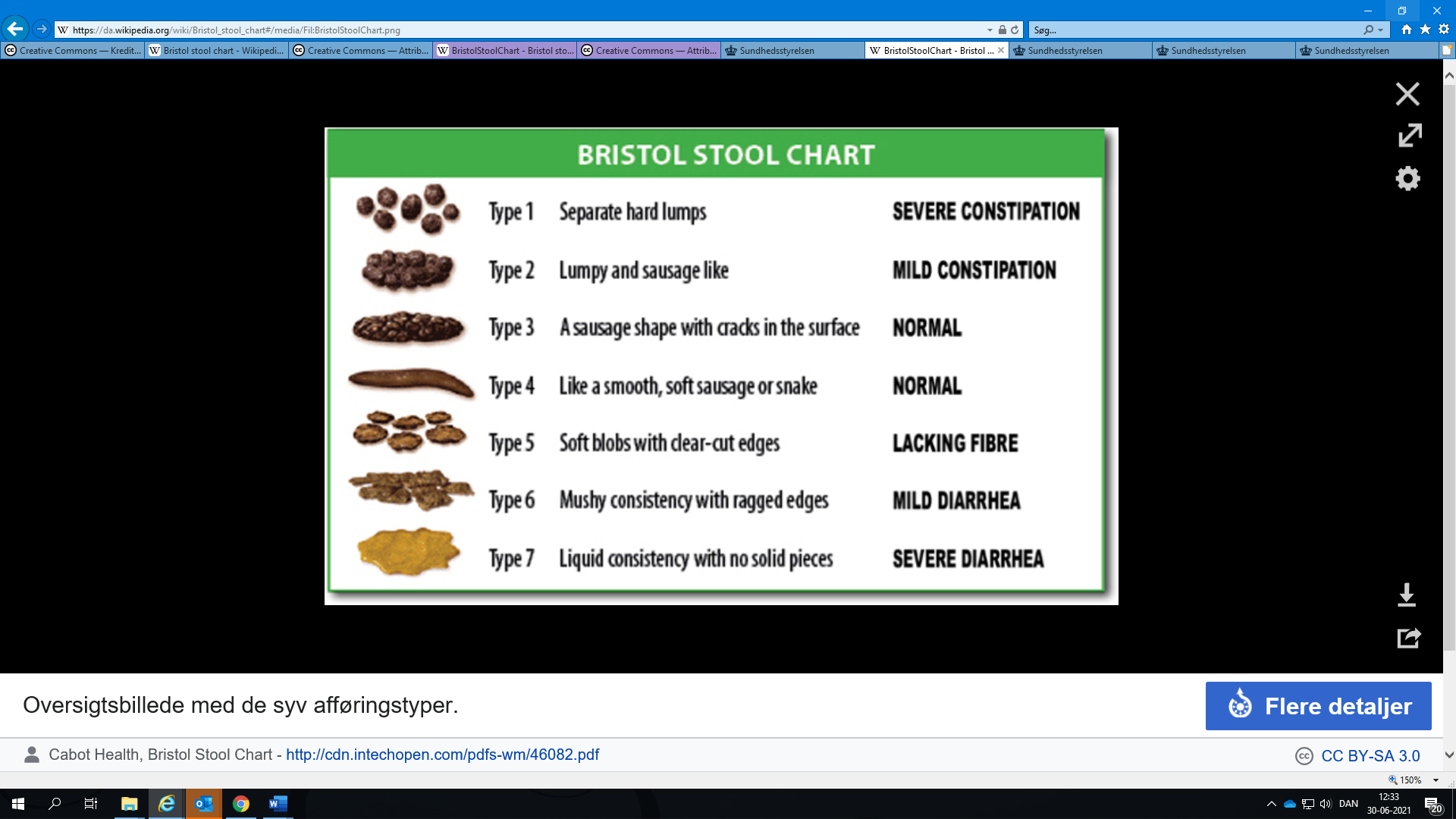  Type 5 Soft blobs  (clear cut edges) |  |  |  |  |  |  |  |  |
| 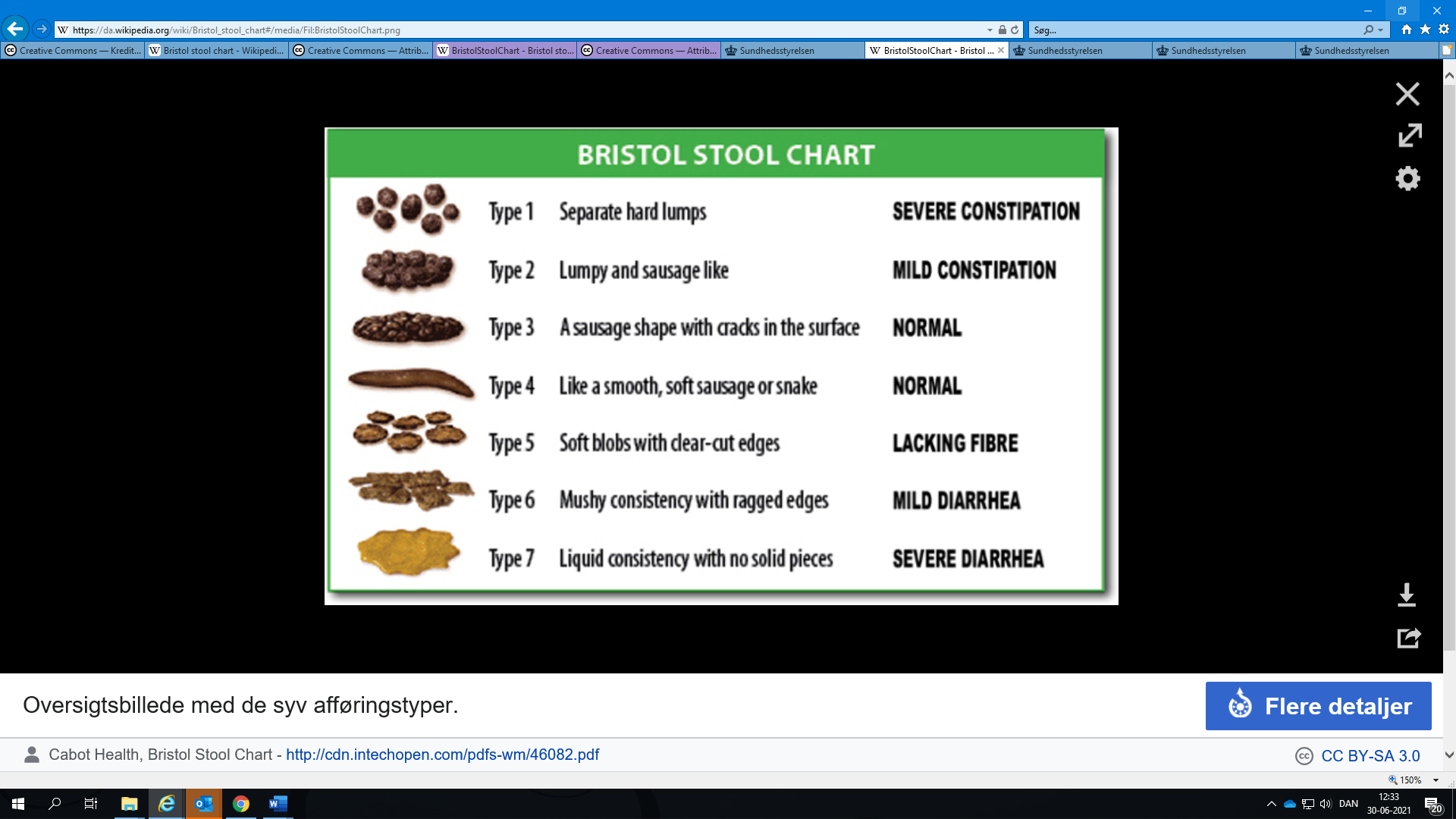 Mushy stool  Type 6 (fluffy small pieces,  ragged edged) |  |  |  |  |  |  |  |  |
| 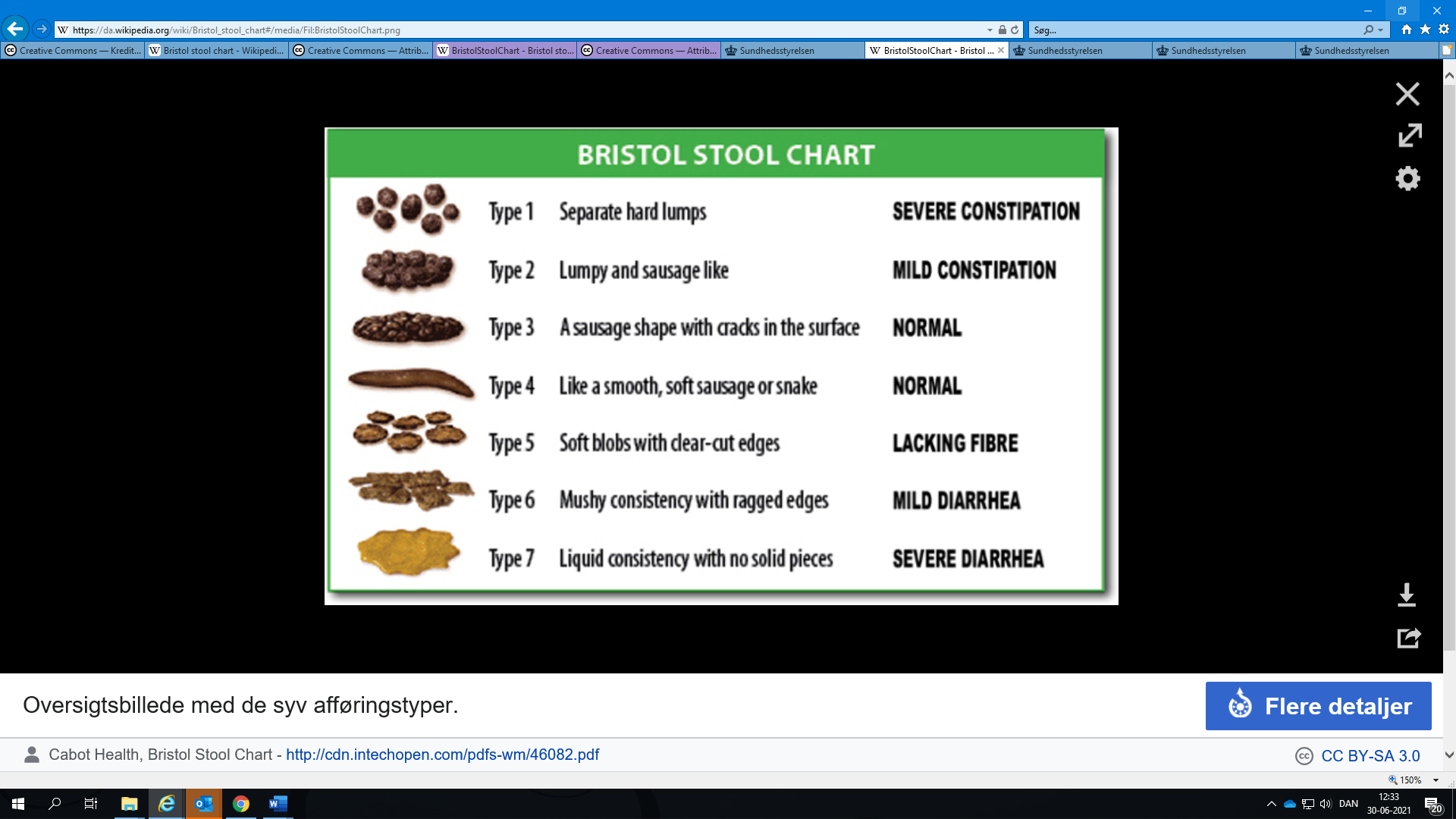  Type 7 Watery  (no solid pieces) |  |  |  |  |  |  |  |  |

**Week 3**

| **Diary Bristol scale for stool types** | | | | | | | | **Comments** |
| --- | --- | --- | --- | --- | --- | --- | --- | --- |
| **Date** |  |  |  |  |  |  |  |  |
| **Body weight** | **kg** |  |  |  |  |  |  |  |
| **Day of the week** | **day** | **day** | **day** | **day** | **day** | **day** | **day** |  |
| 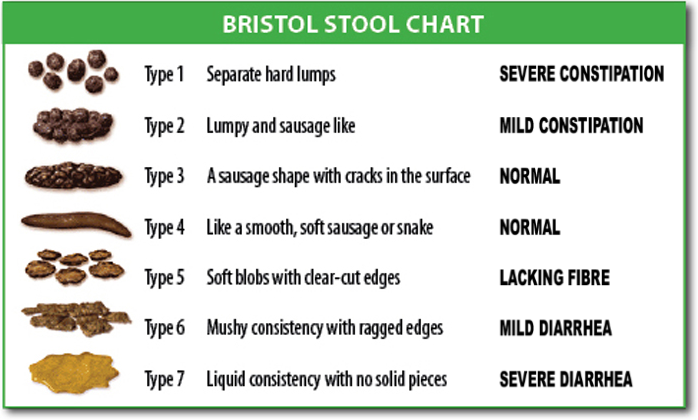  Type 1 Hard lumps |  |  |  |  |  |  |  |  |
| 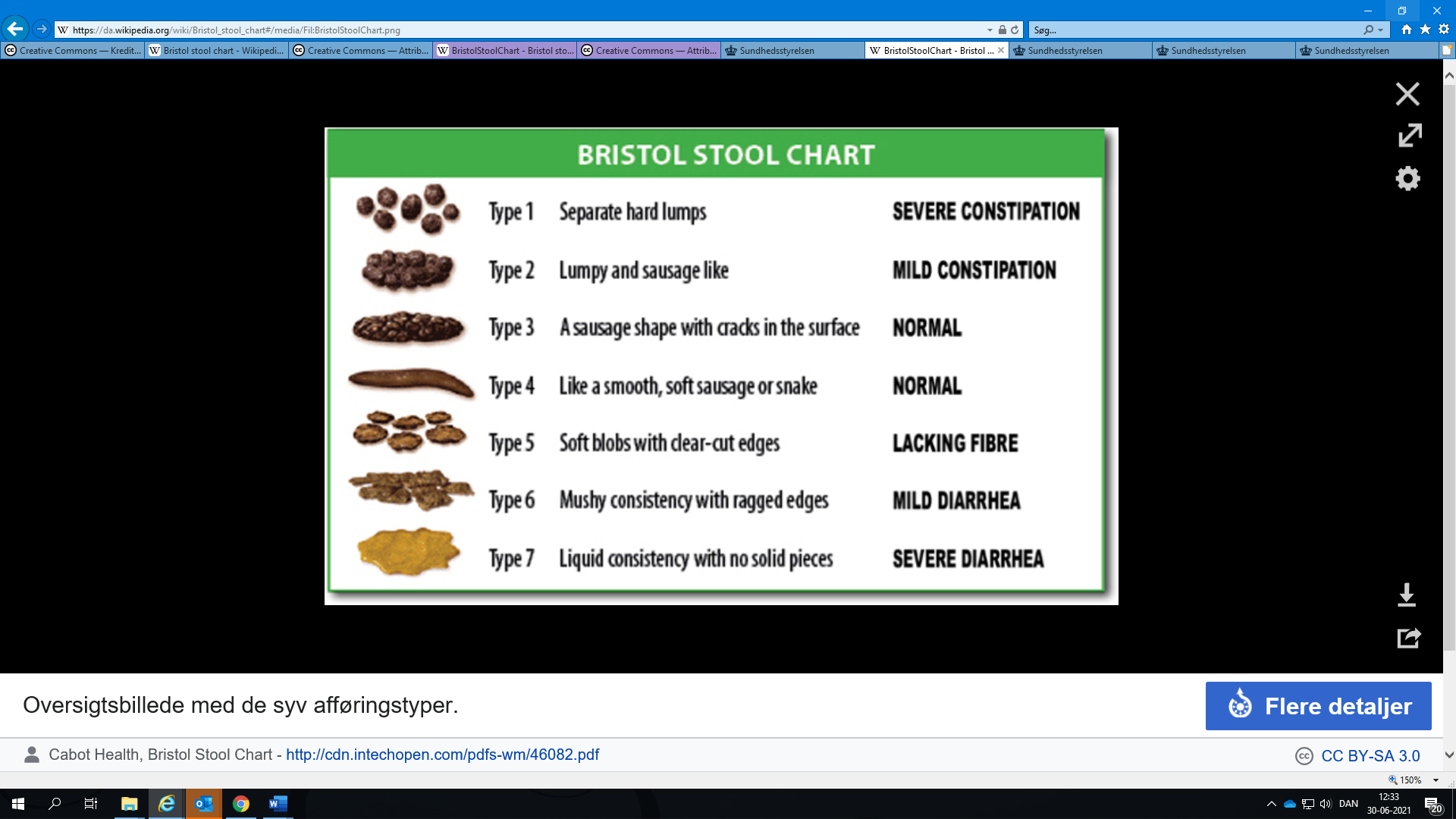  Type 2 Sausage-shaped  (lumpy surface) |  |  |  |  |  |  |  |  |
| 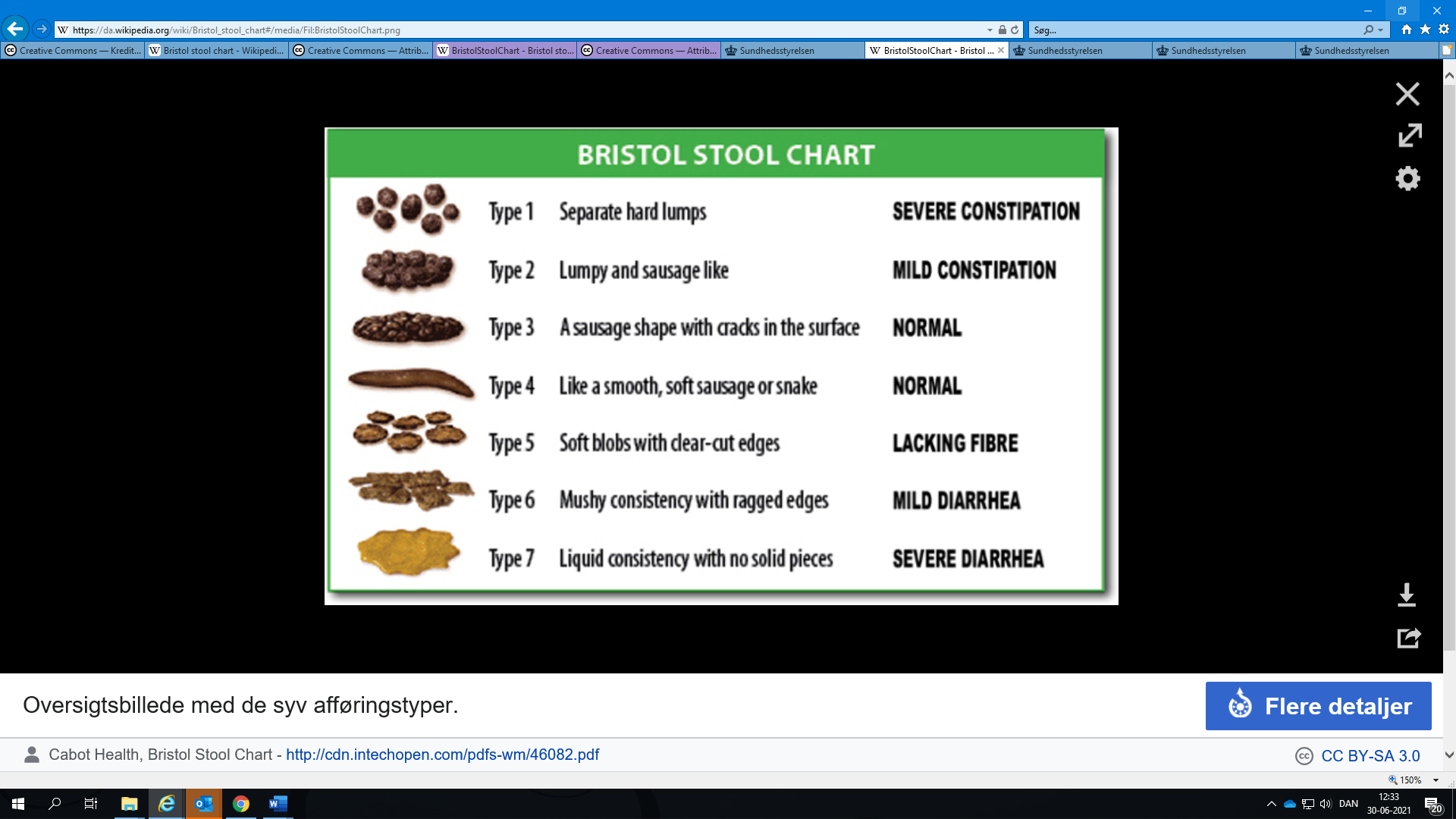  Type 3 Sausage-shaped  (cracked) |  |  |  |  |  |  |  |  |
| 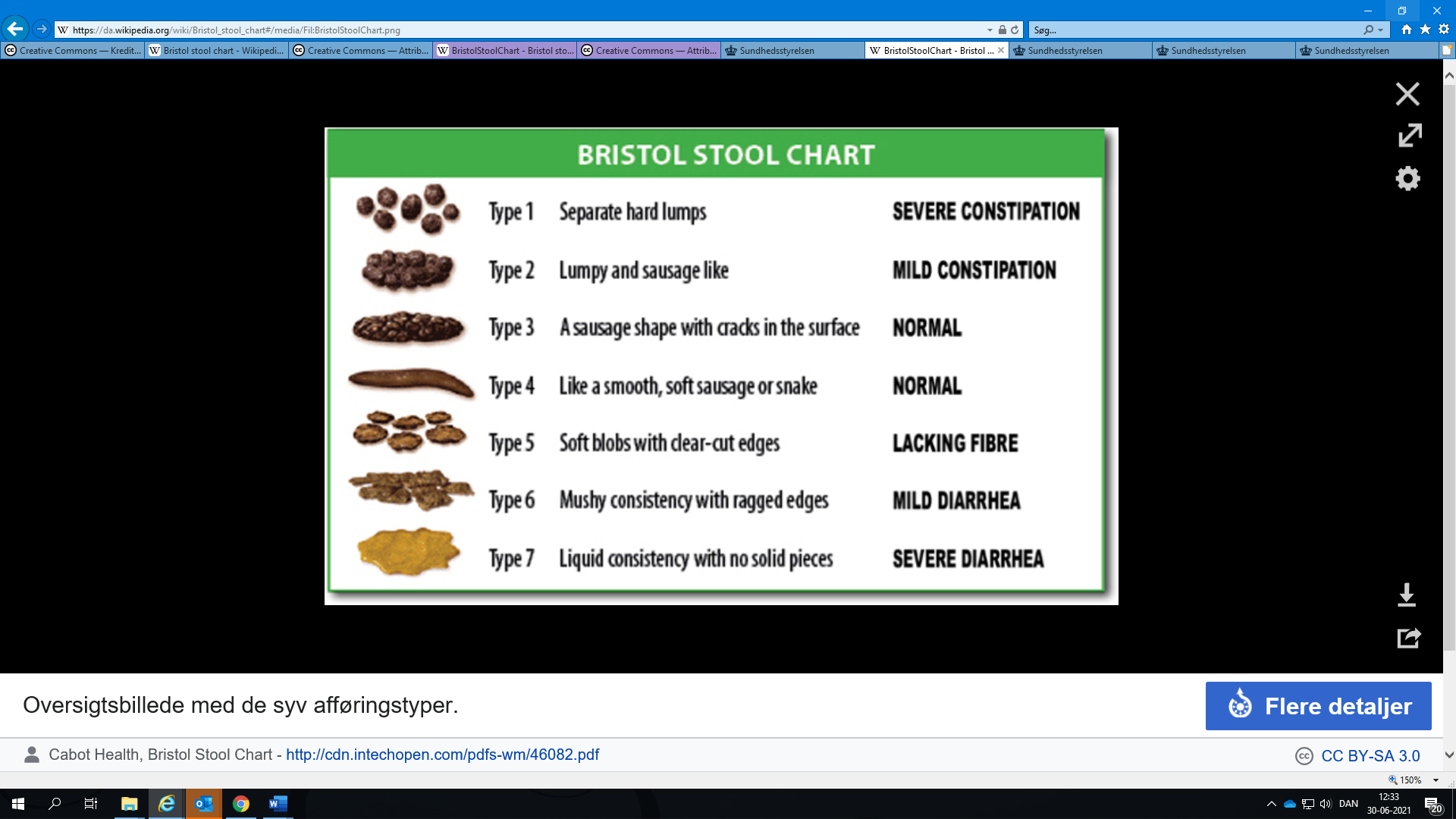  Type 4 Sausage or snake-shaped  (smooth and soft) |  |  |  |  |  |  |  |  |
| 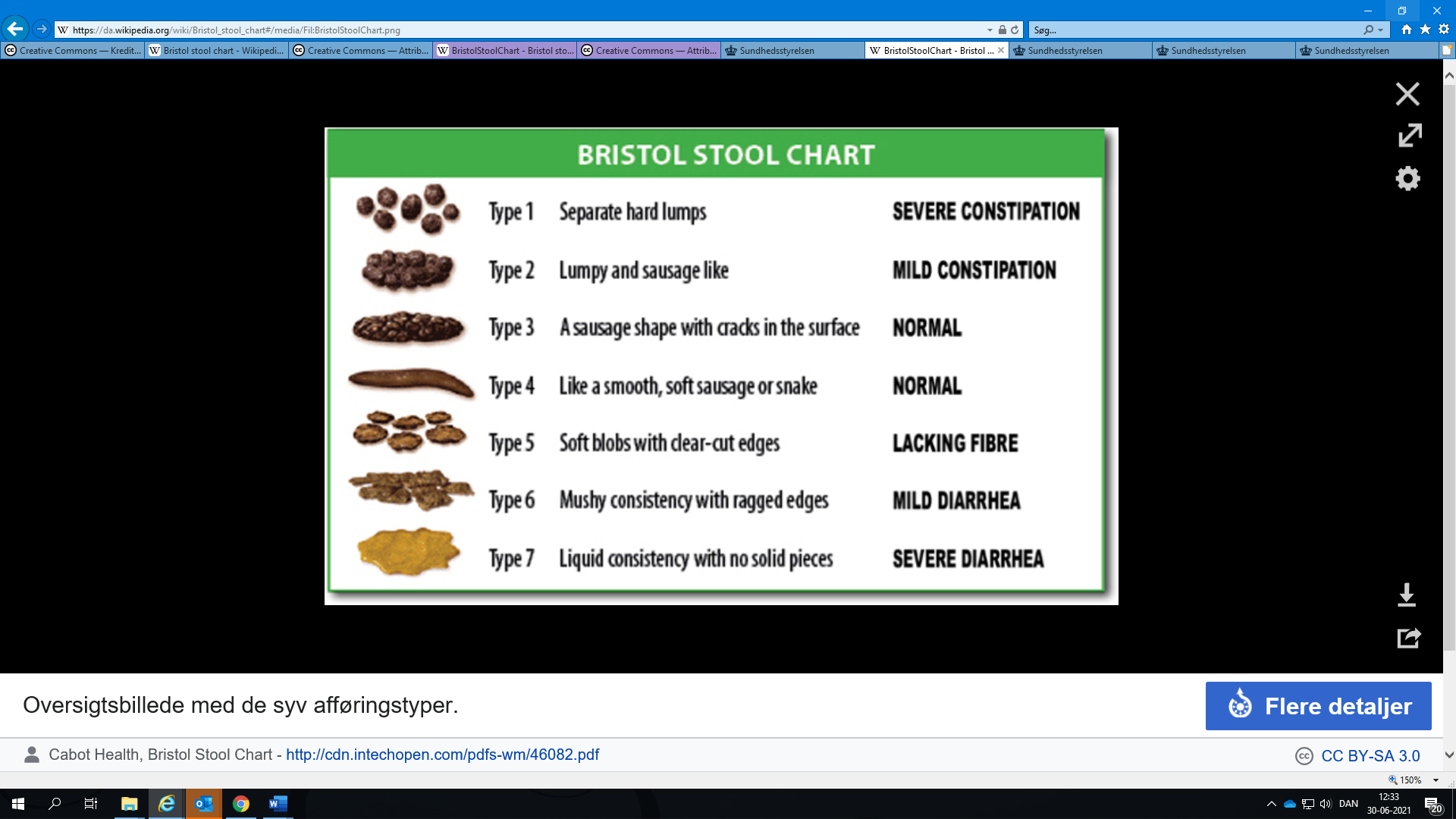  Type 5 Soft blobs  (clear cut edges) |  |  |  |  |  |  |  |  |
| 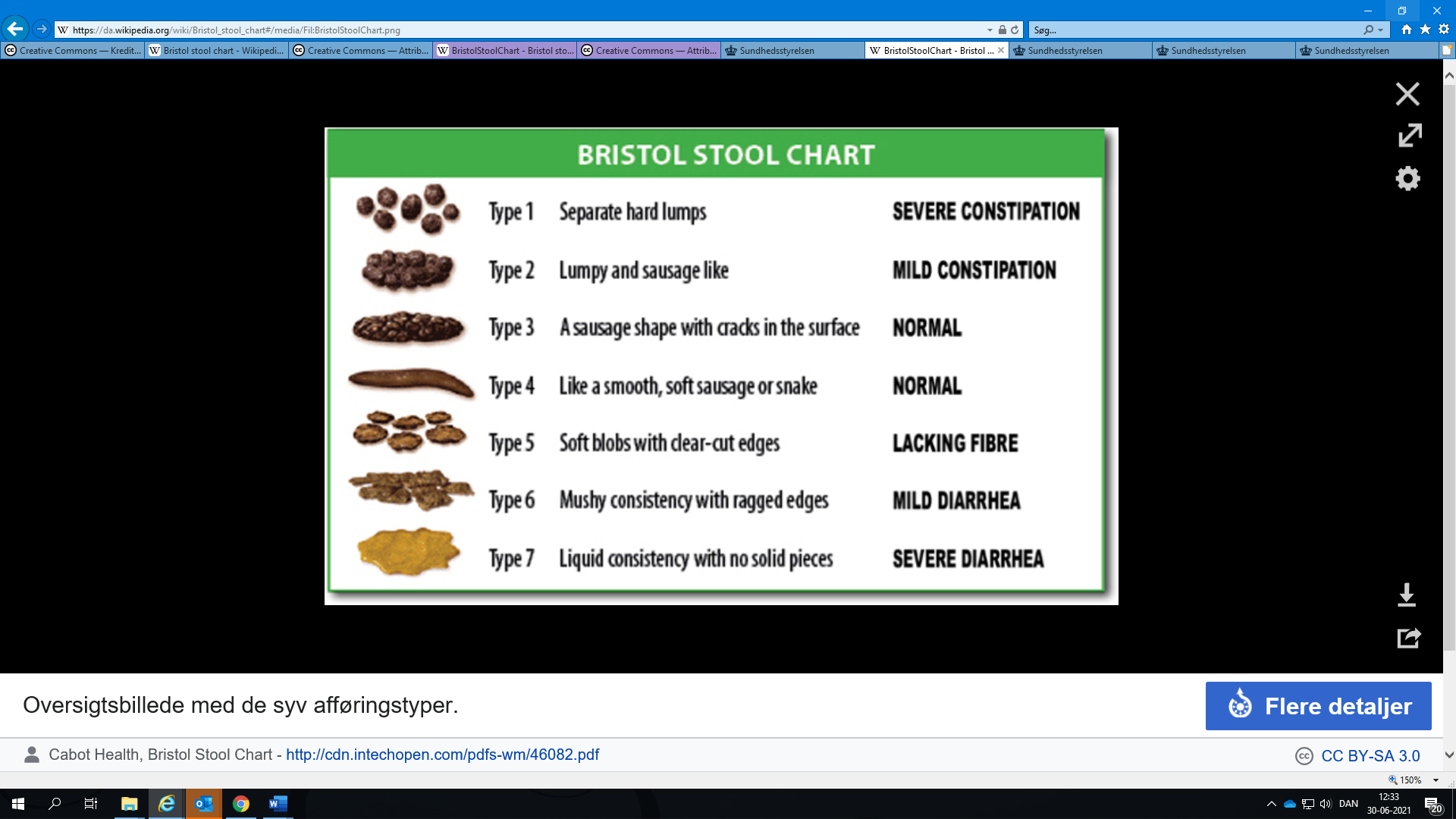 Mushy stool  Type 6 (fluffy small pieces,  ragged edged) |  |  |  |  |  |  |  |  |
| 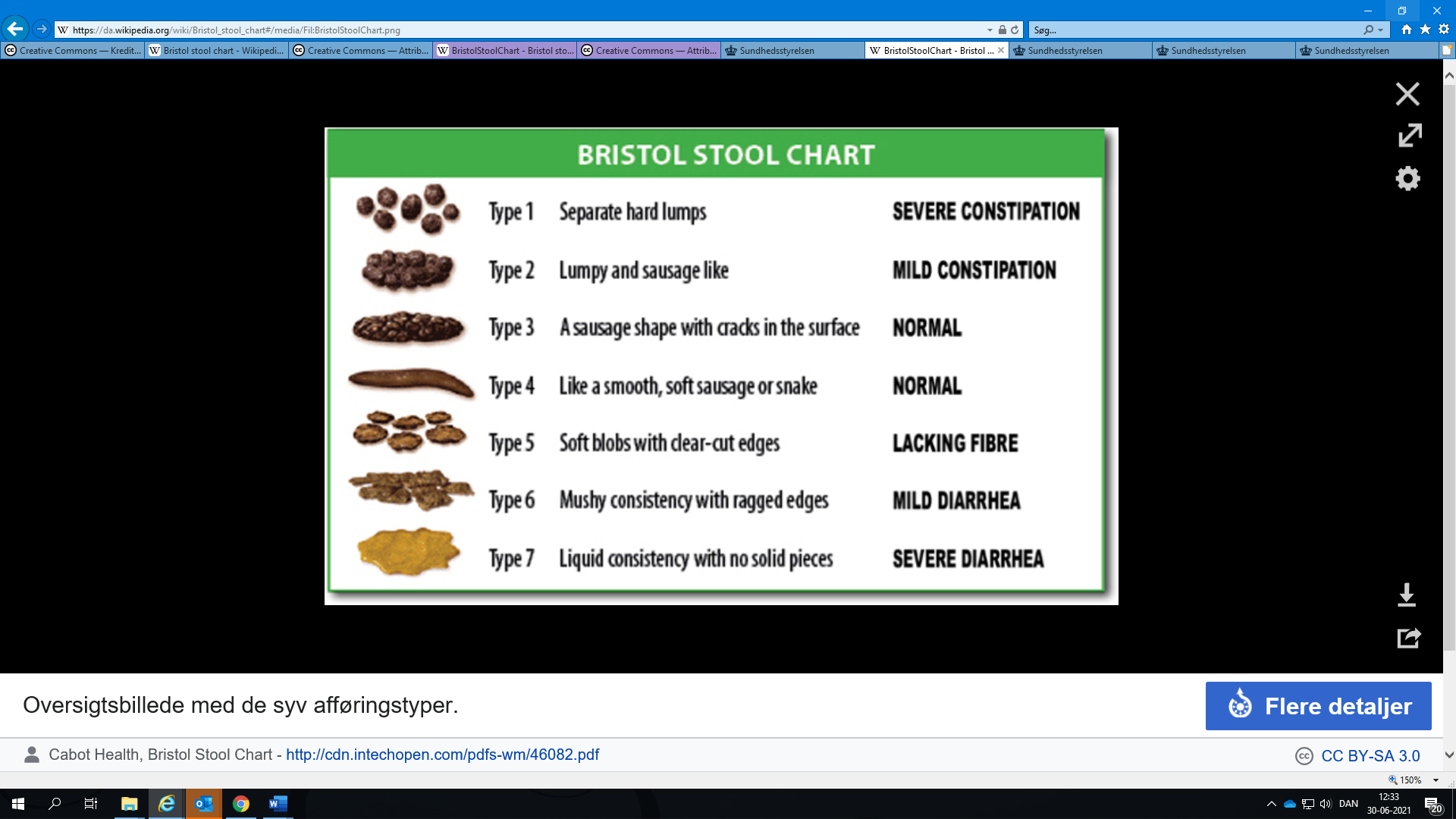  Type 7 Watery  (no solid pieces) |  |  |  |  |  |  |  |  |

**Week 4**

| **Diary Bristol scale for stool types** | | | | | | | | **Comments** |
| --- | --- | --- | --- | --- | --- | --- | --- | --- |
| **Date** |  |  |  |  |  |  |  |  |
| **Body weight** | **kg** |  |  |  |  |  |  |  |
| **Day of the week** | **day** | **day** | **day** | **day** | **day** | **day** | **day** |  |
| 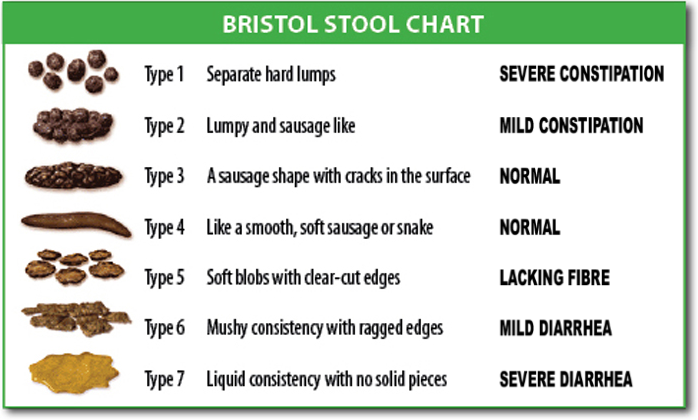  Type 1 Hard lumps |  |  |  |  |  |  |  |  |
| 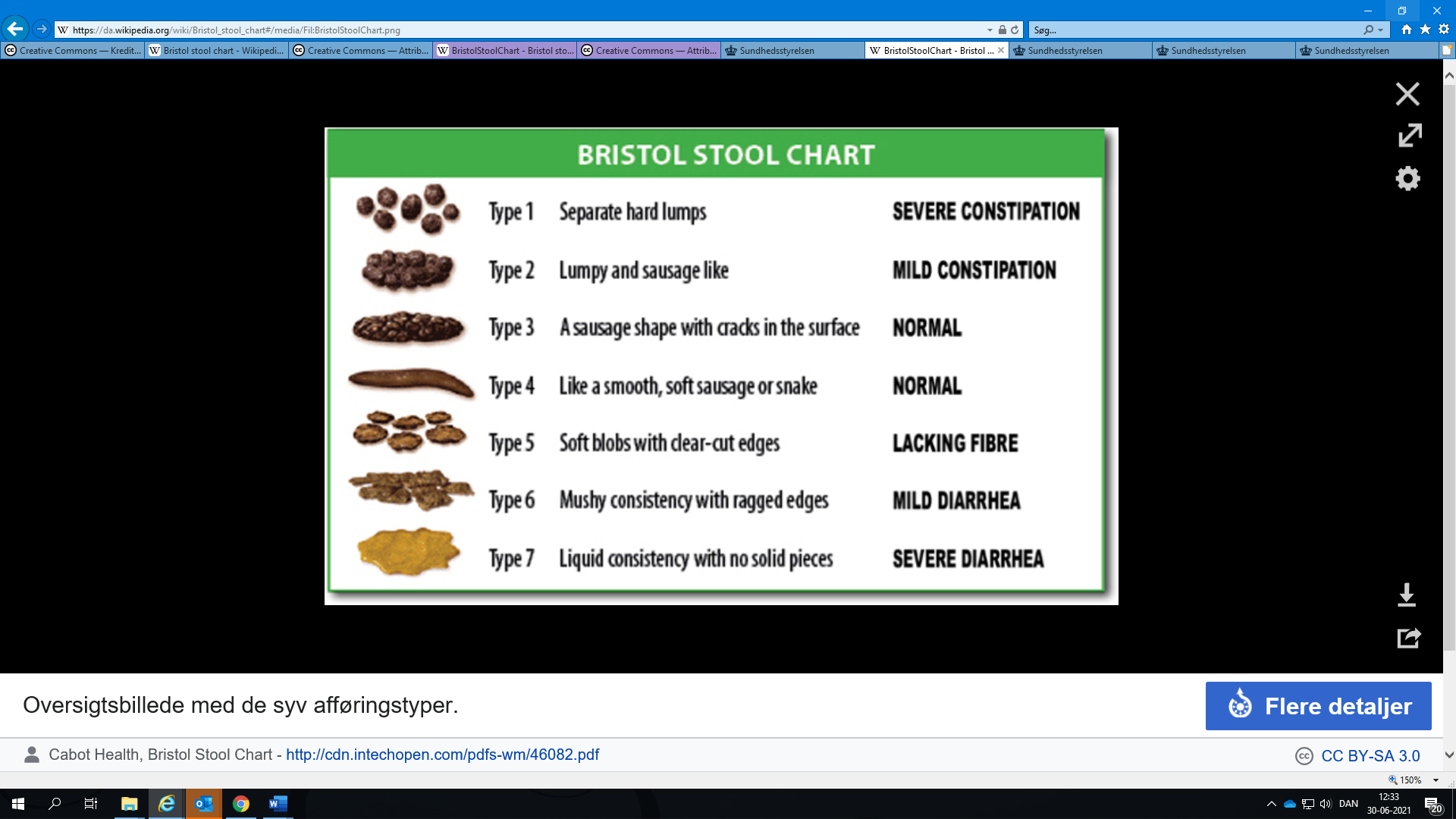  Type 2 Sausage-shaped  (lumpy surface) |  |  |  |  |  |  |  |  |
| 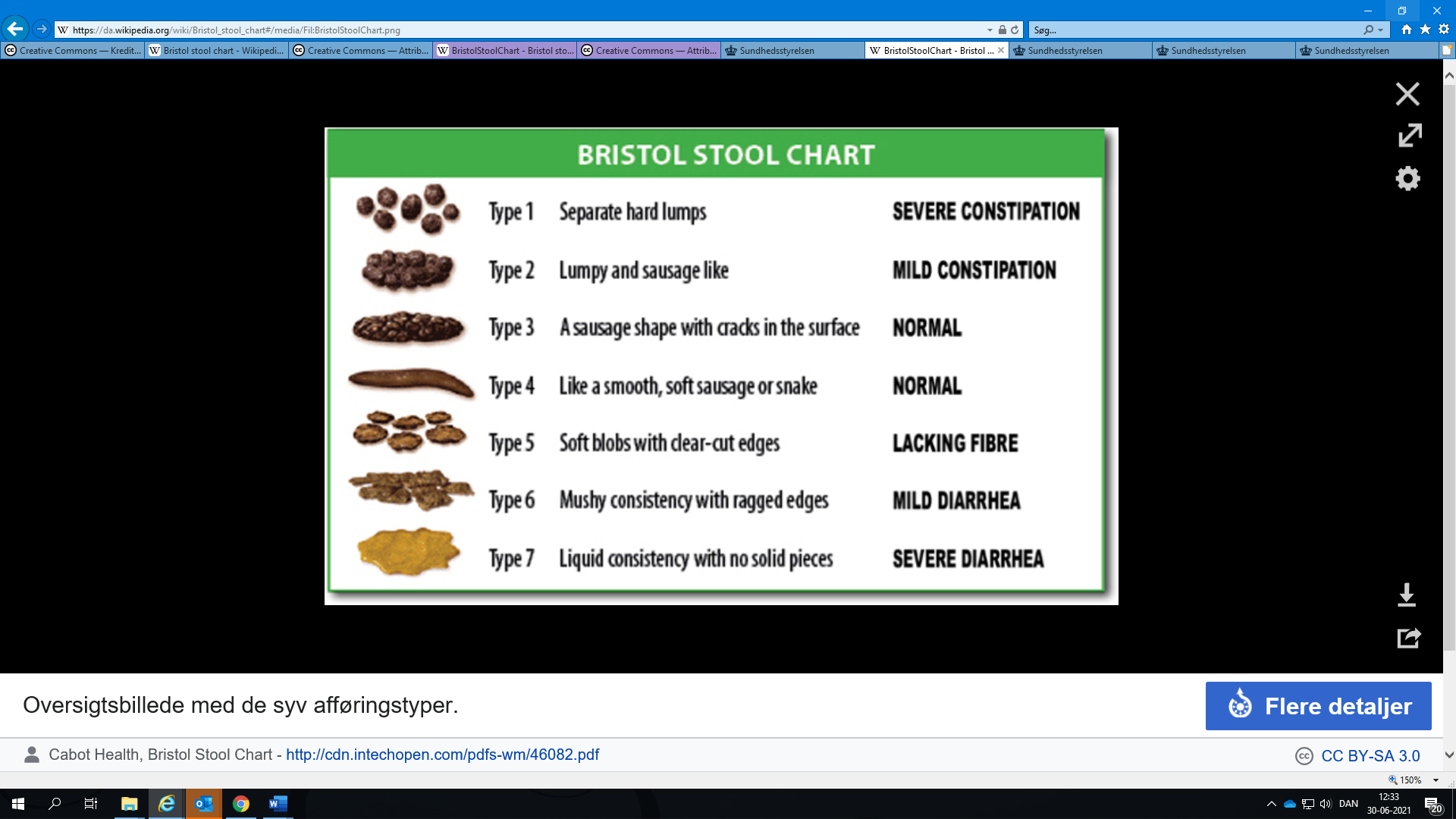  Type 3 Sausage-shaped  (cracked) |  |  |  |  |  |  |  |  |
| 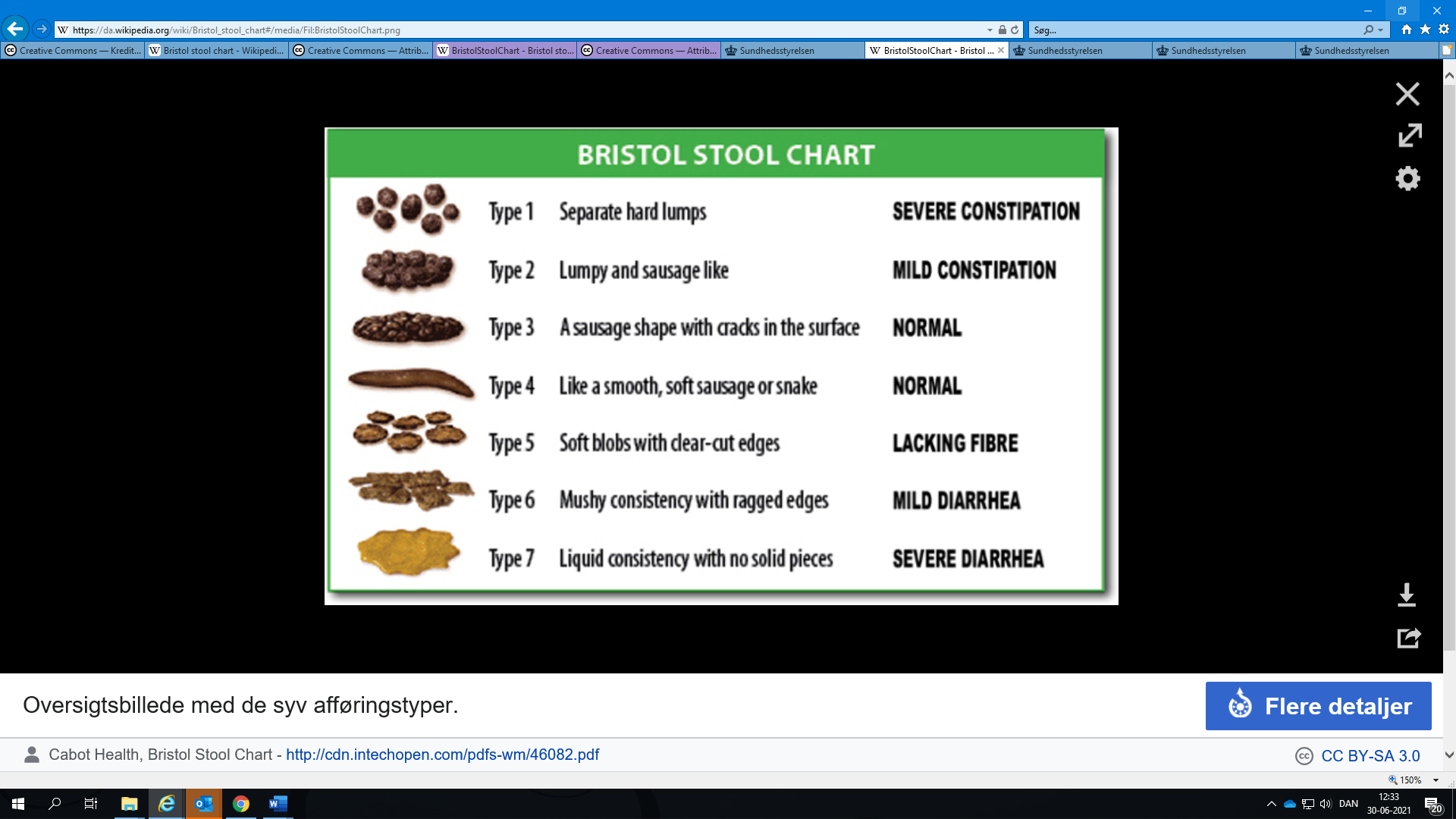  Type 4 Sausage or snake-shaped  (smooth and soft) |  |  |  |  |  |  |  |  |
| 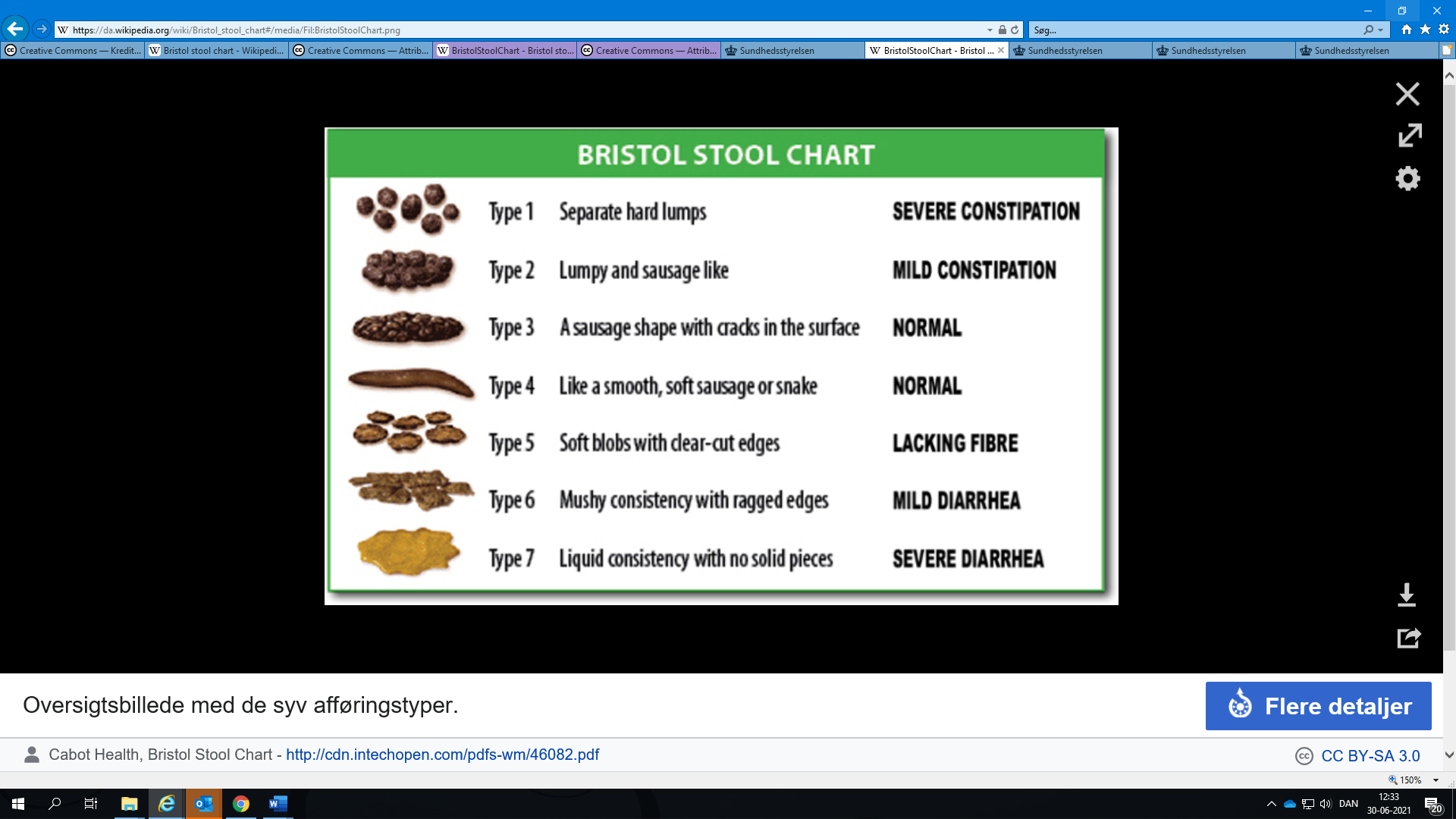  Type 5 Soft blobs  (clear cut edges) |  |  |  |  |  |  |  |  |
| 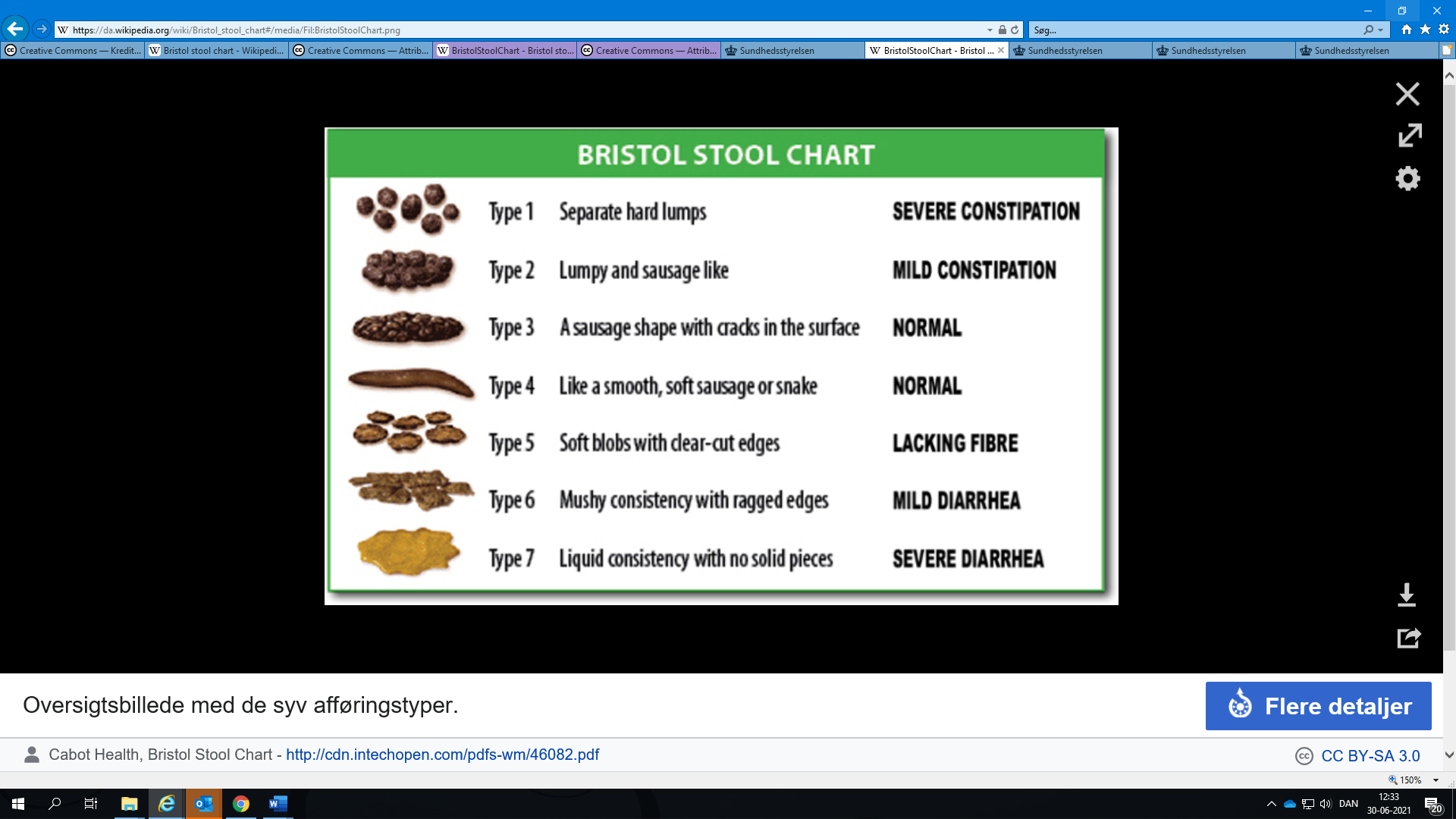 Mushy stool  Type 6 (fluffy small pieces,  ragged edged) |  |  |  |  |  |  |  |  |
| 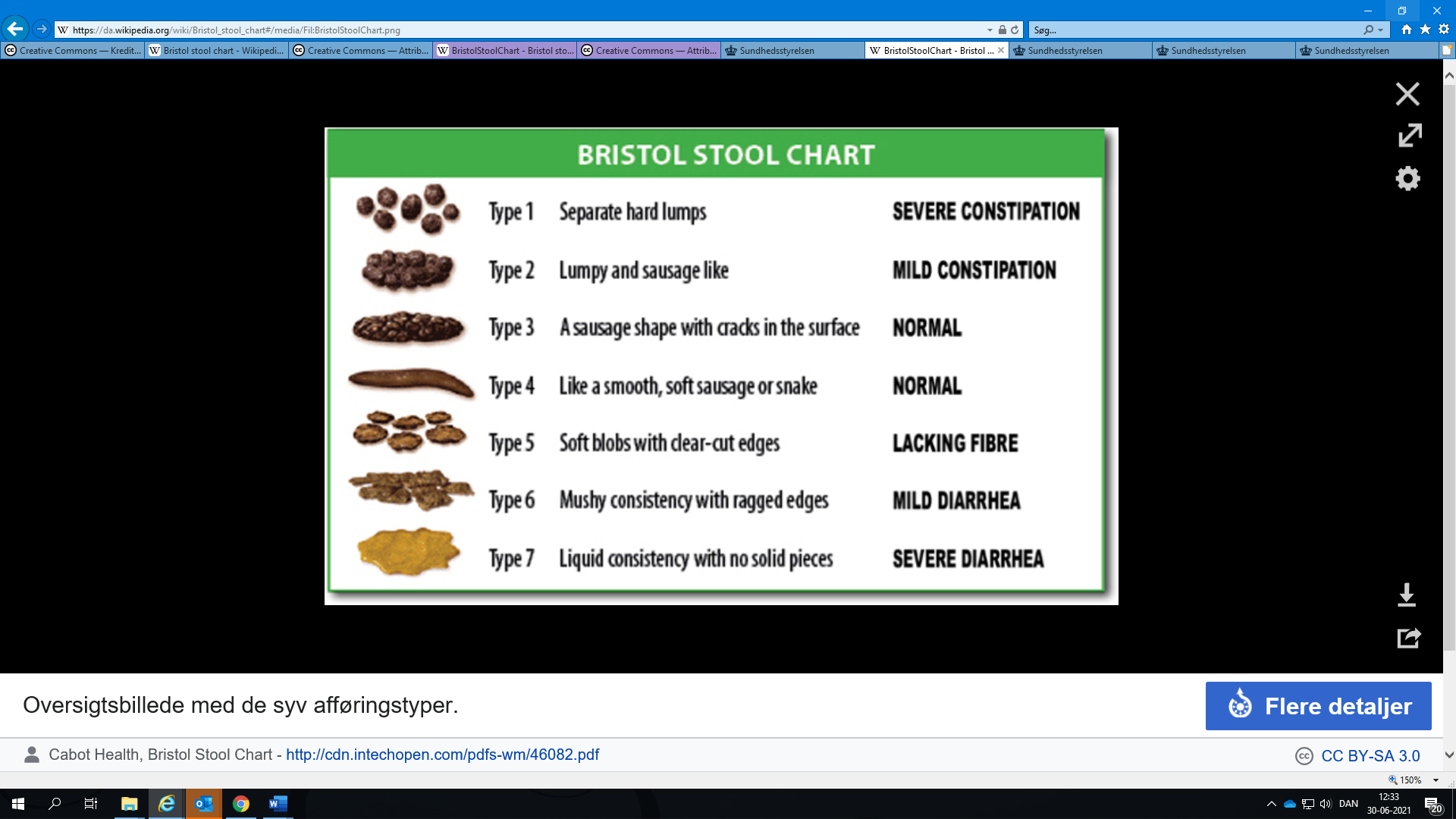  Type 7 Watery  (no solid pieces) |  |  |  |  |  |  |  |  |

**Week 5**

| **Diary Bristol scale for stool types** | | | | | | | | **Comments** |
| --- | --- | --- | --- | --- | --- | --- | --- | --- |
| **Date** |  |  |  |  |  |  |  |  |
| **Body weight** | **kg** |  |  |  |  |  |  |  |
| **Day of the week** | **day** | **day** | **day** | **day** | **day** | **day** | **day** |  |
| 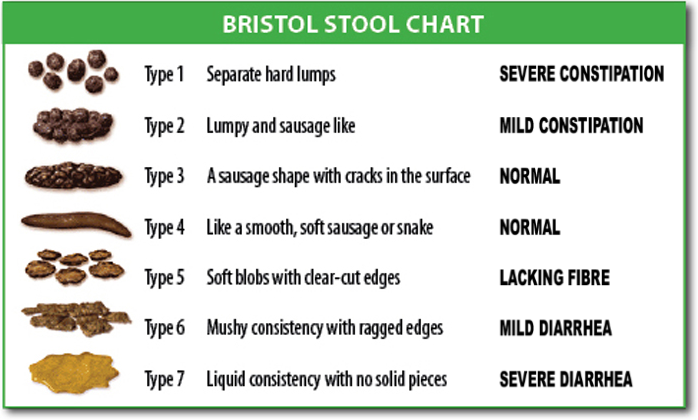  Type 1 Hard lumps |  |  |  |  |  |  |  |  |
| 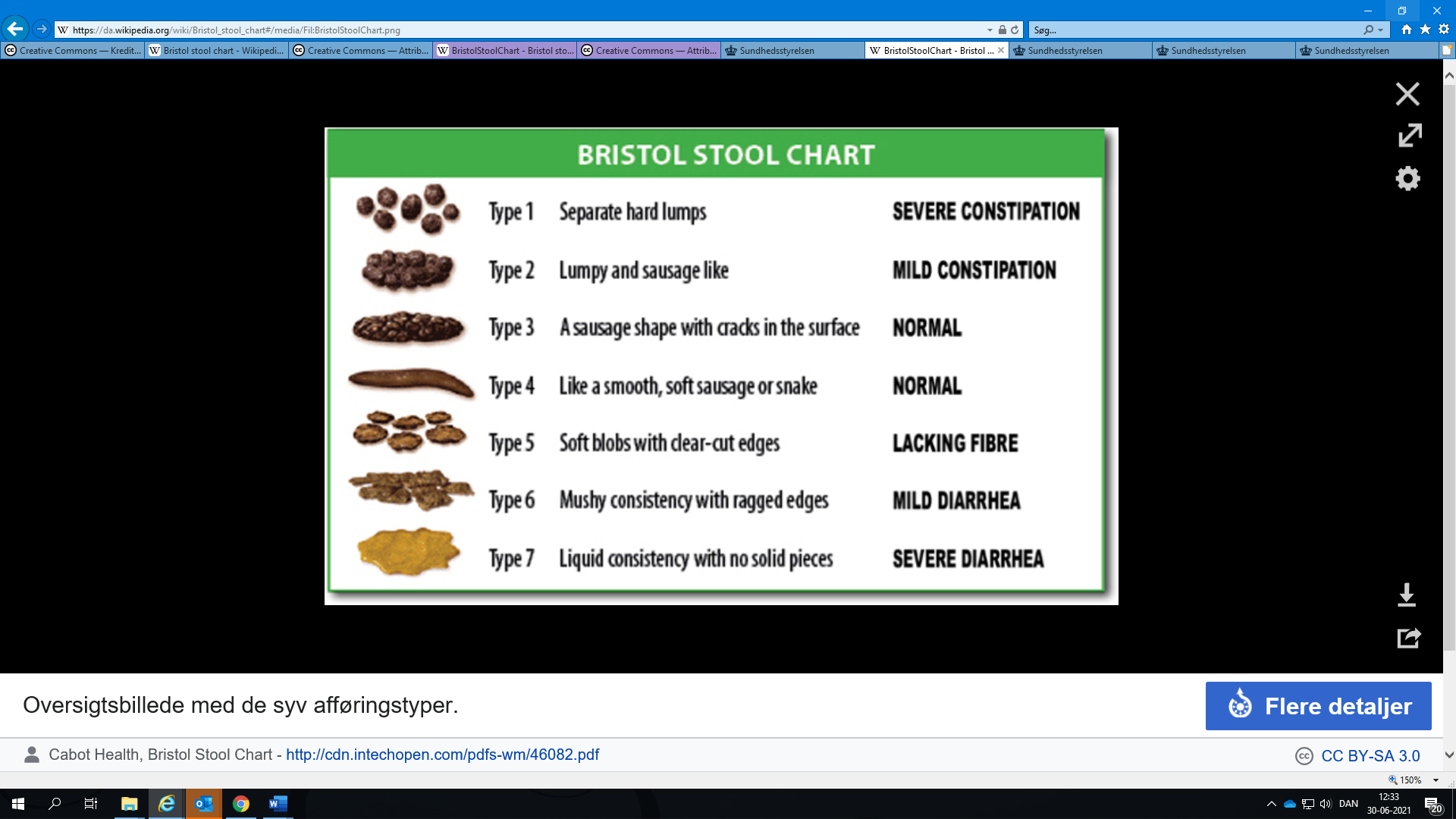  Type 2 Sausage-shaped  (lumpy surface) |  |  |  |  |  |  |  |  |
| 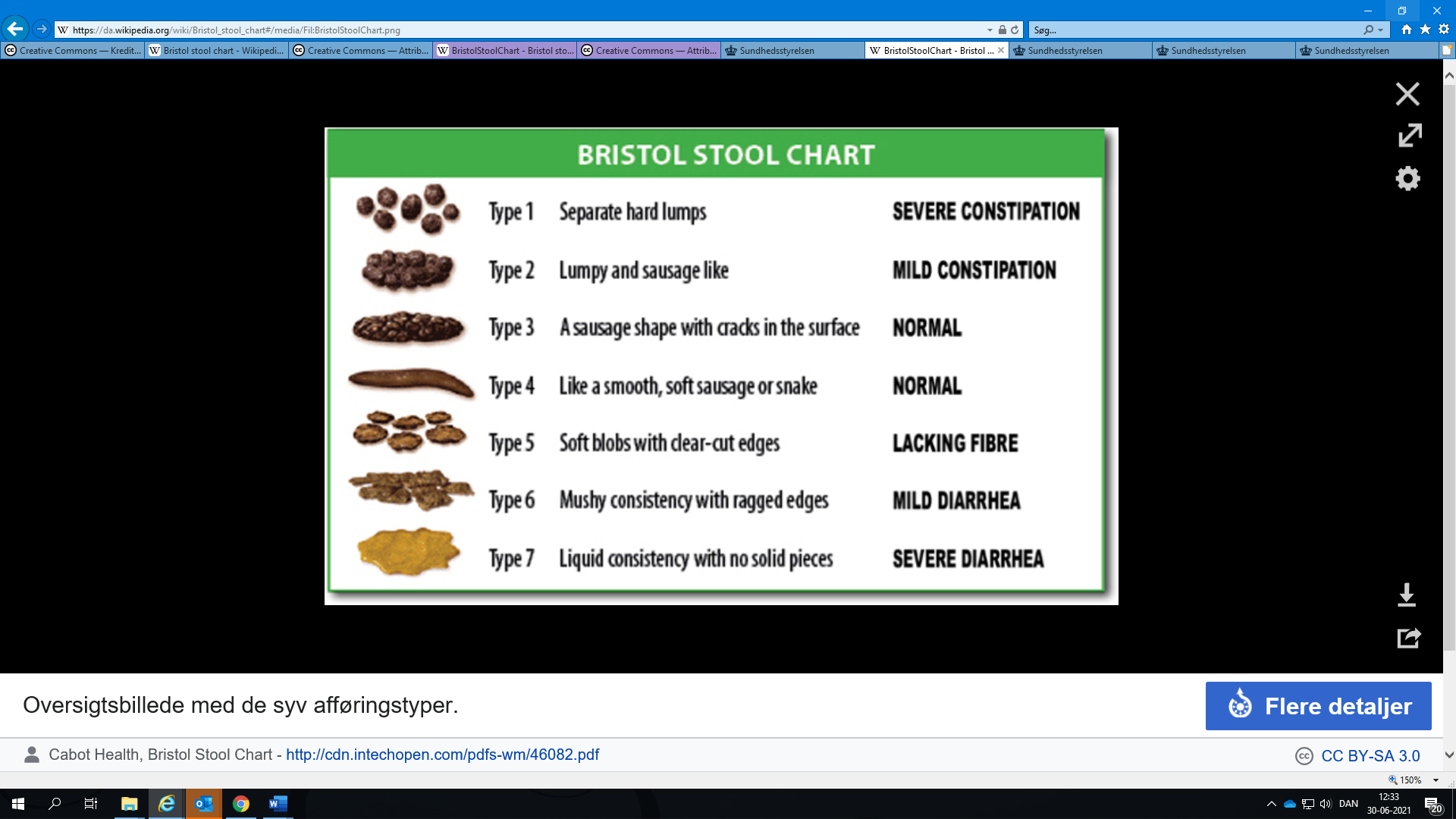  Type 3 Sausage-shaped  (cracked) |  |  |  |  |  |  |  |  |
| 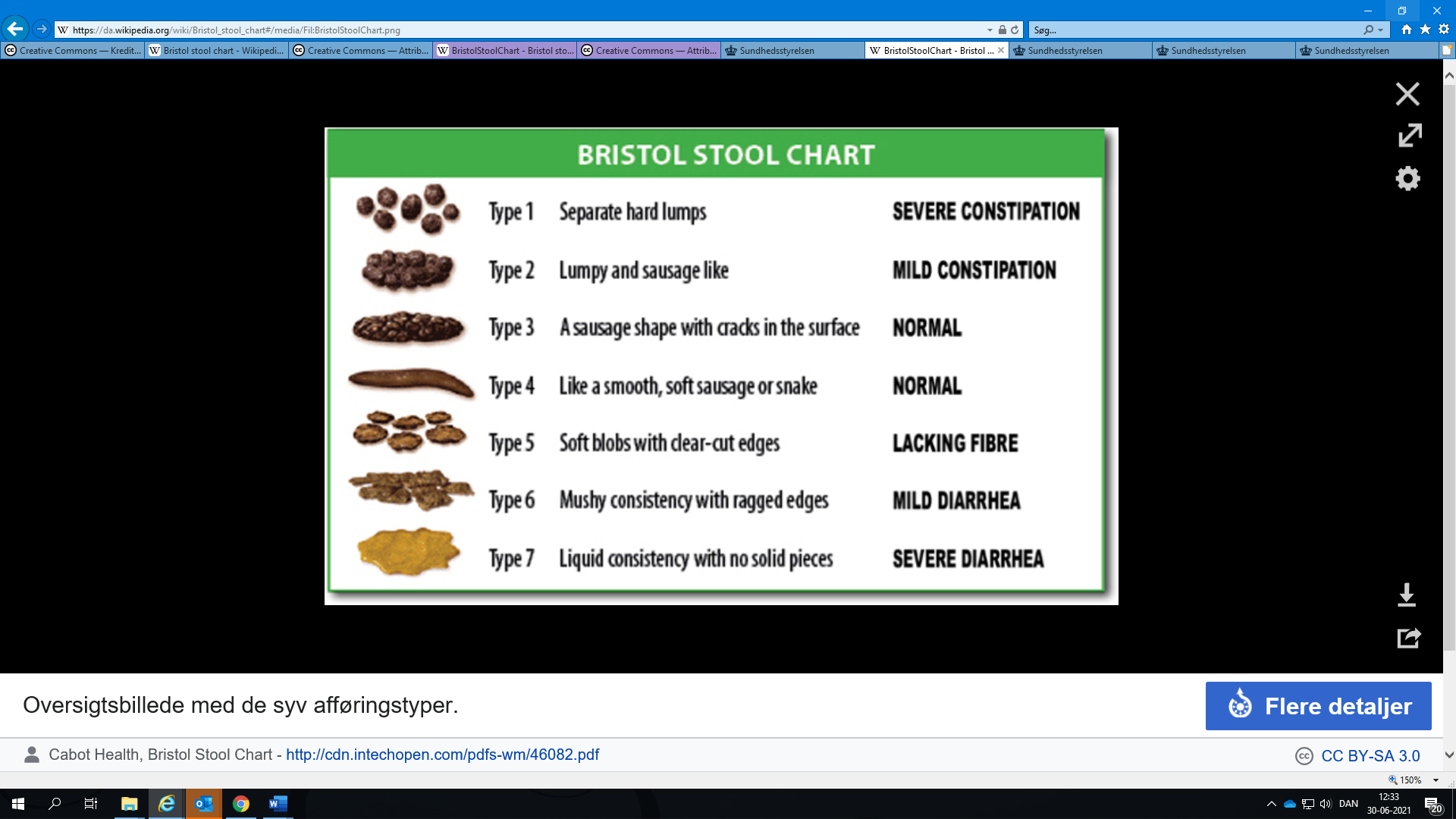  Type 4 Sausage or snake-shaped  (smooth and soft) |  |  |  |  |  |  |  |  |
| 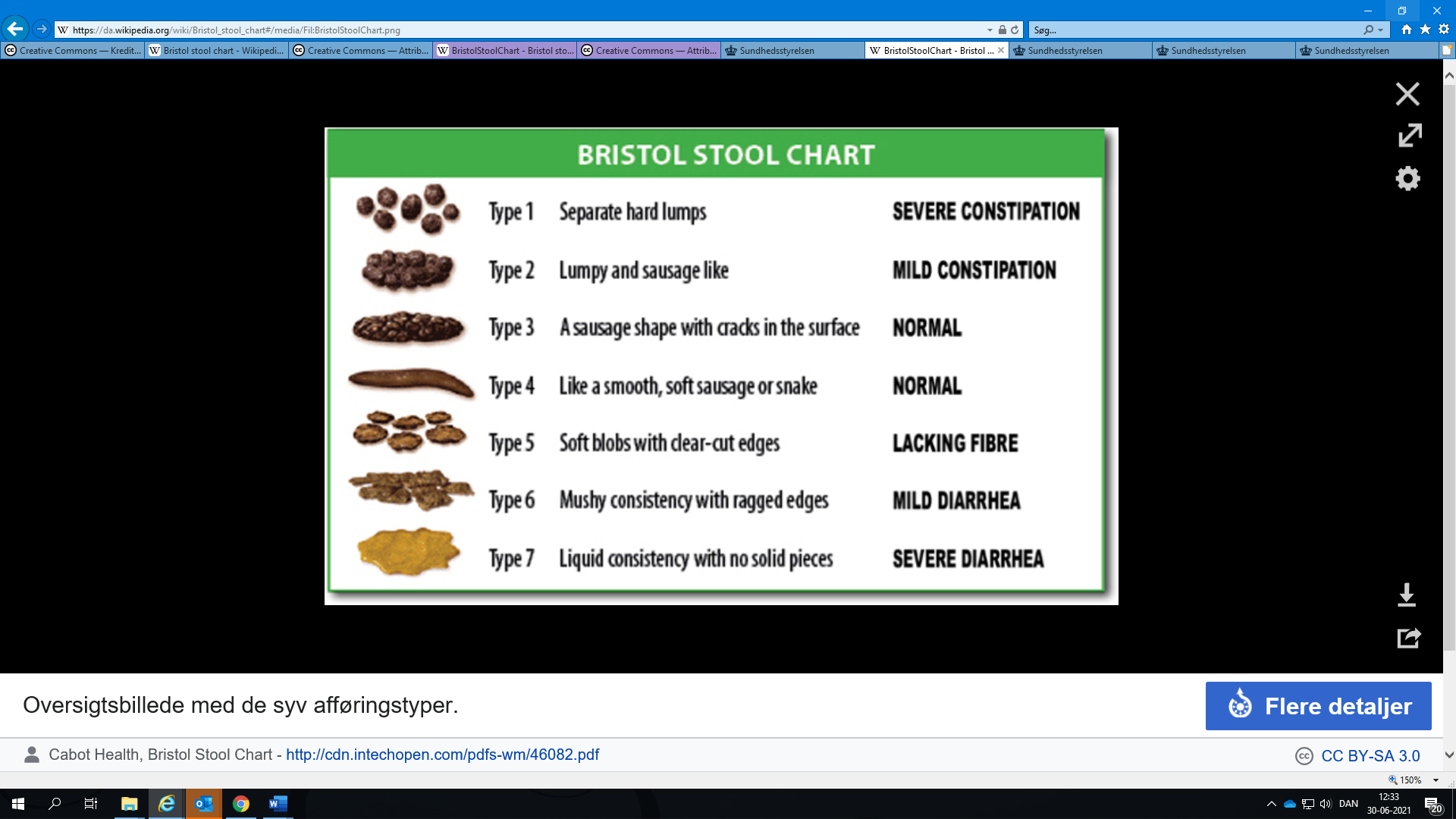  Type 5 Soft blobs  (clear cut edges) |  |  |  |  |  |  |  |  |
| 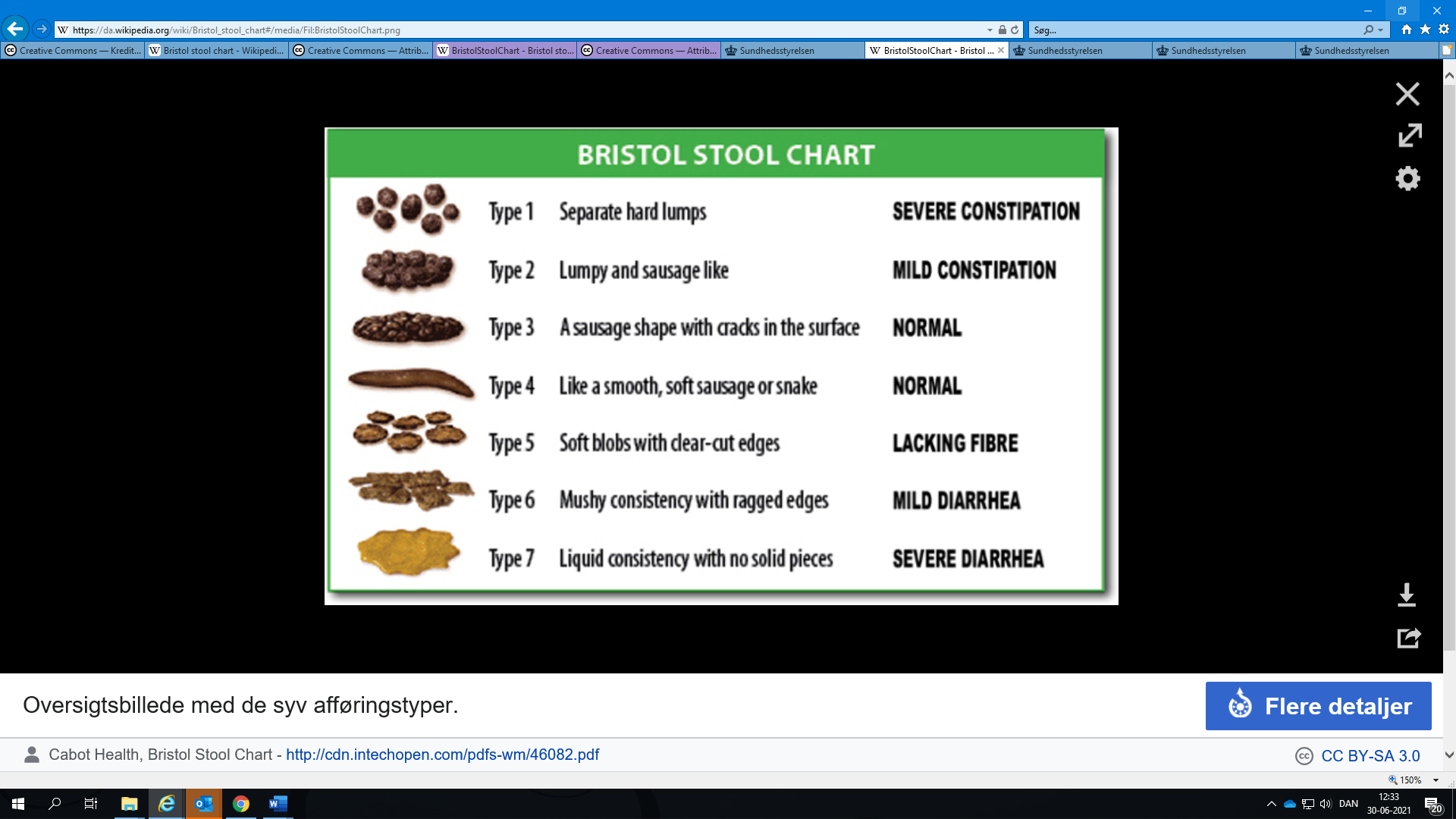 Mushy stool  Type 6 (fluffy small pieces,  ragged edged) |  |  |  |  |  |  |  |  |
| 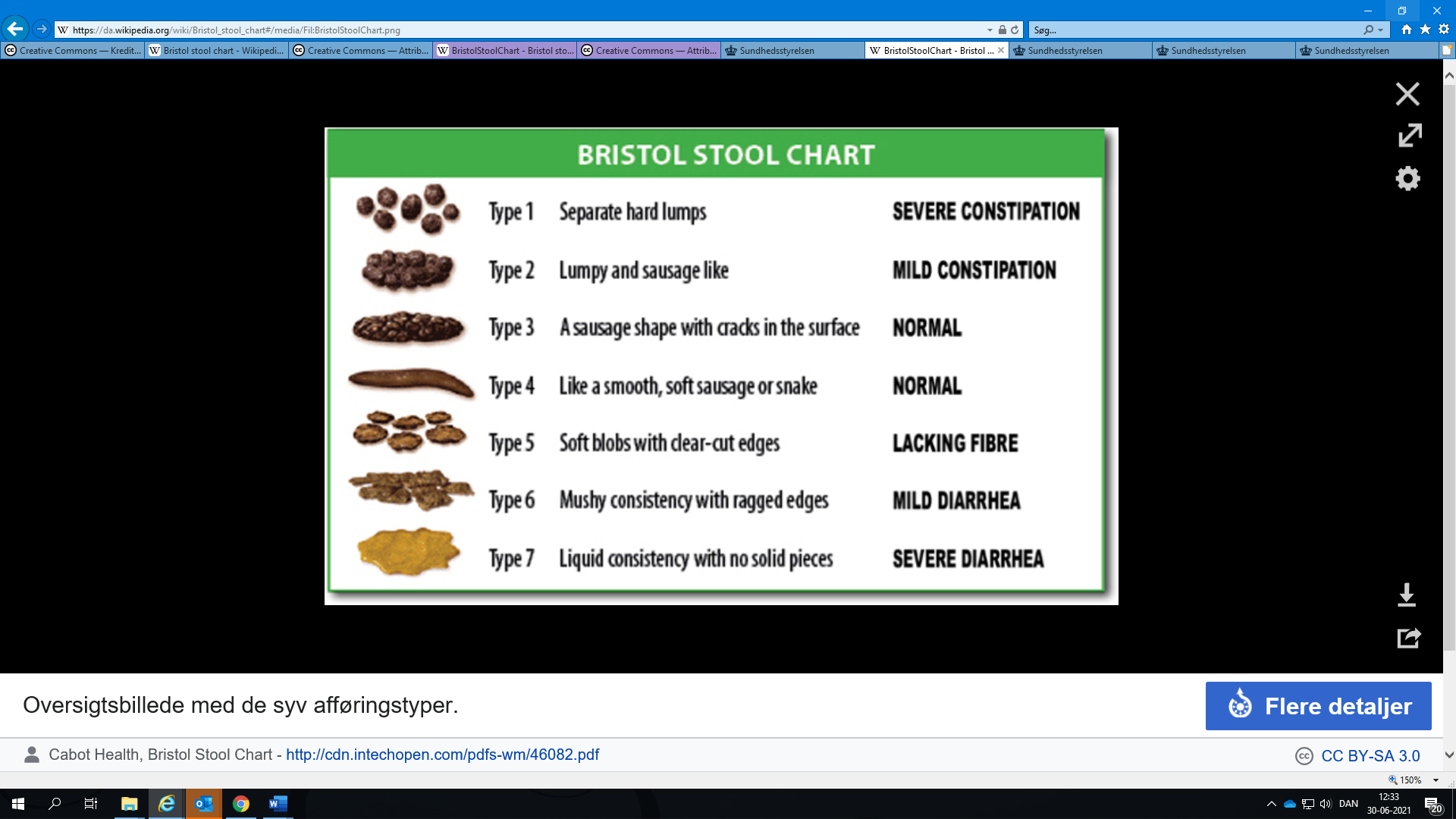  Type 7 Watery  (no solid pieces) |  |  |  |  |  |  |  |  |

**Week 6**

| **Diary Bristol scale for stool types** | | | | | | | | **Comments** |
| --- | --- | --- | --- | --- | --- | --- | --- | --- |
| **Date** |  |  |  |  |  |  |  |  |
| **Body weight** | **kg** |  |  |  |  |  |  |  |
| **Day of the week** | **day** | **day** | **day** | **day** | **day** | **day** | **day** |  |
| 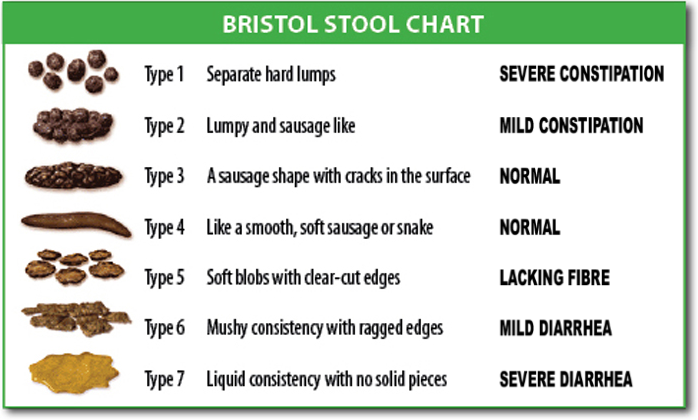  Type 1 Hard lumps |  |  |  |  |  |  |  |  |
| 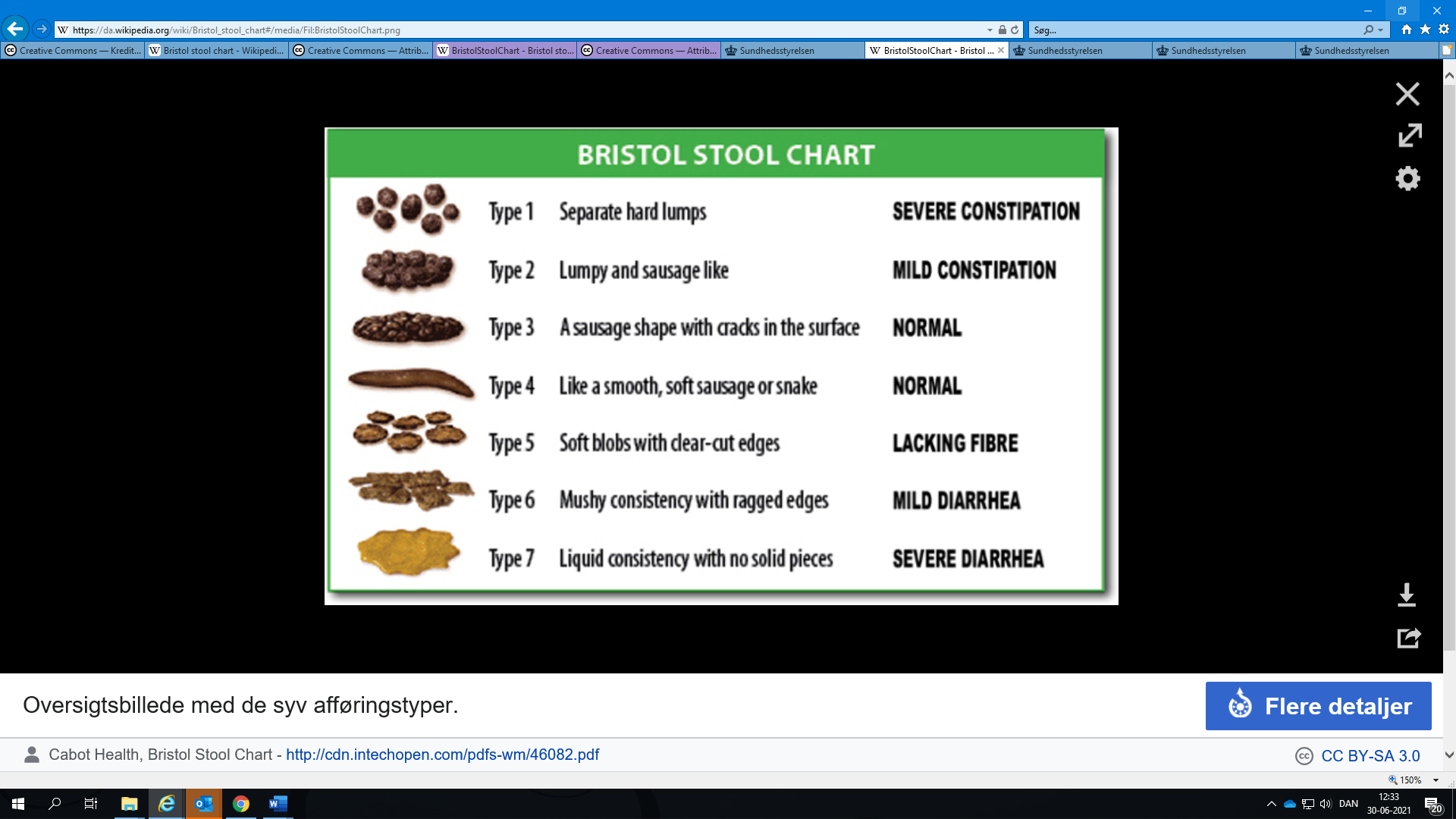  Type 2 Sausage-shaped  (lumpy surface) |  |  |  |  |  |  |  |  |
| 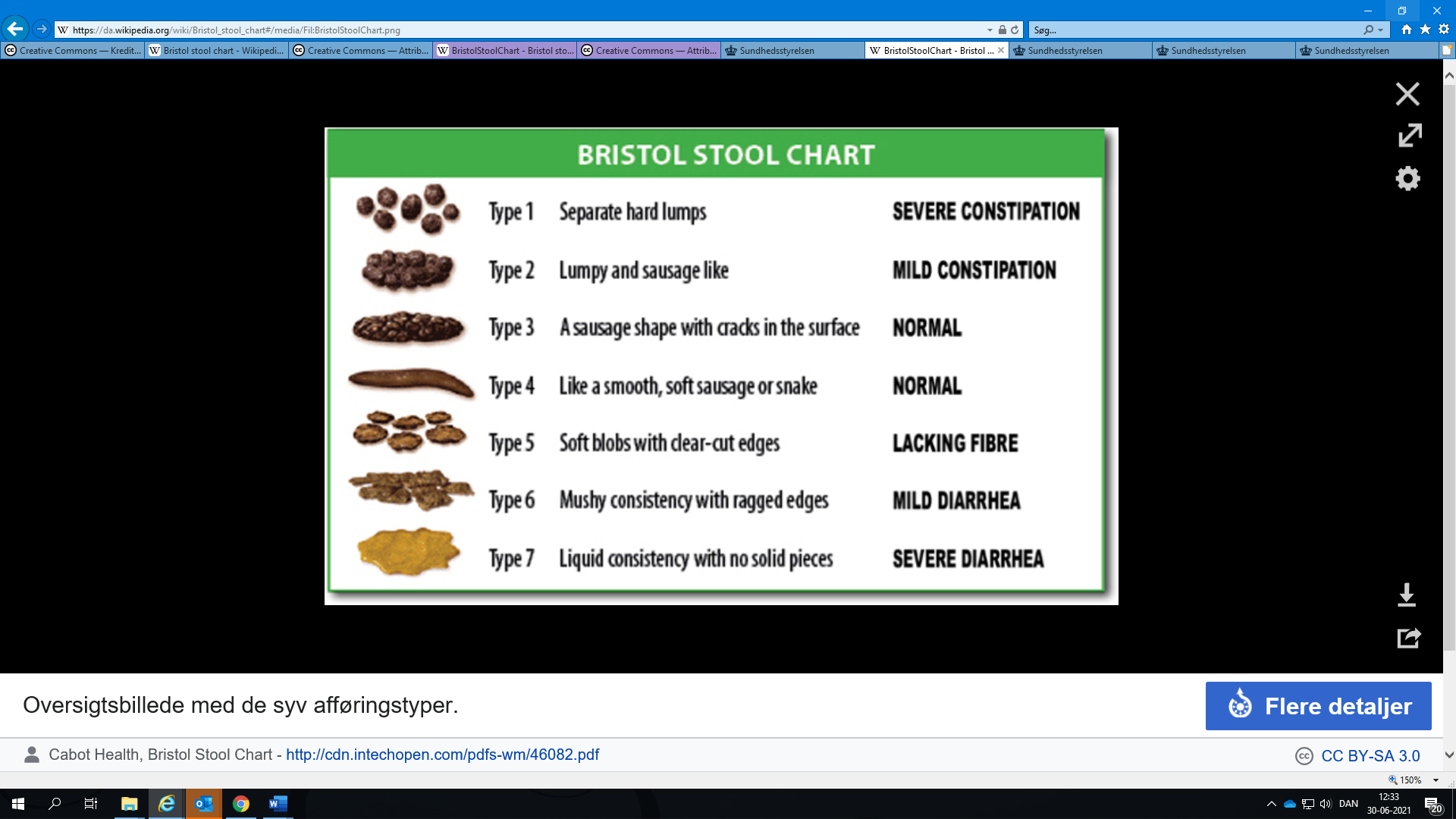  Type 3 Sausage-shaped  (cracked) |  |  |  |  |  |  |  |  |
| 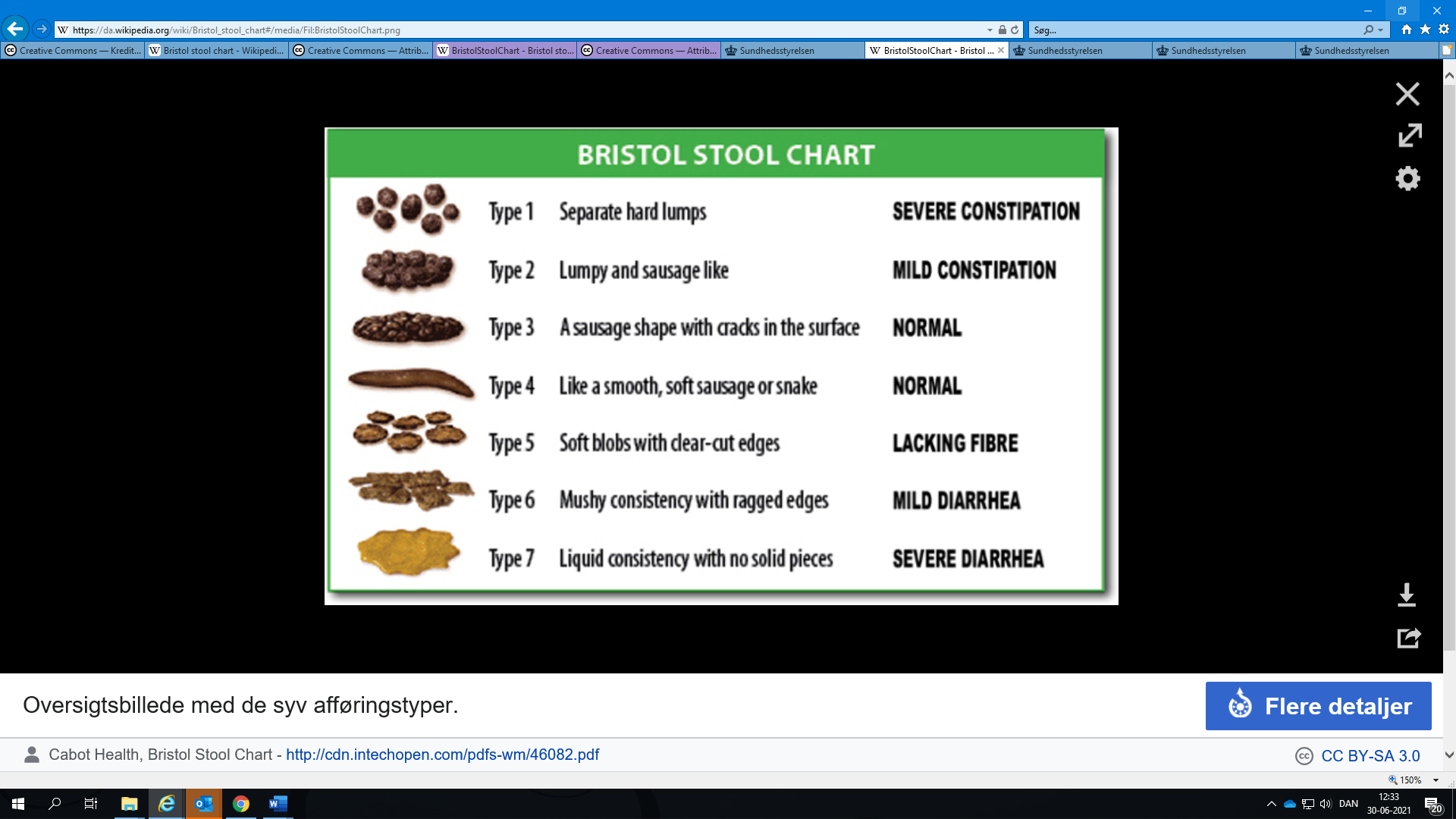  Type 4 Sausage or snake-shaped  (smooth and soft) |  |  |  |  |  |  |  |  |
| 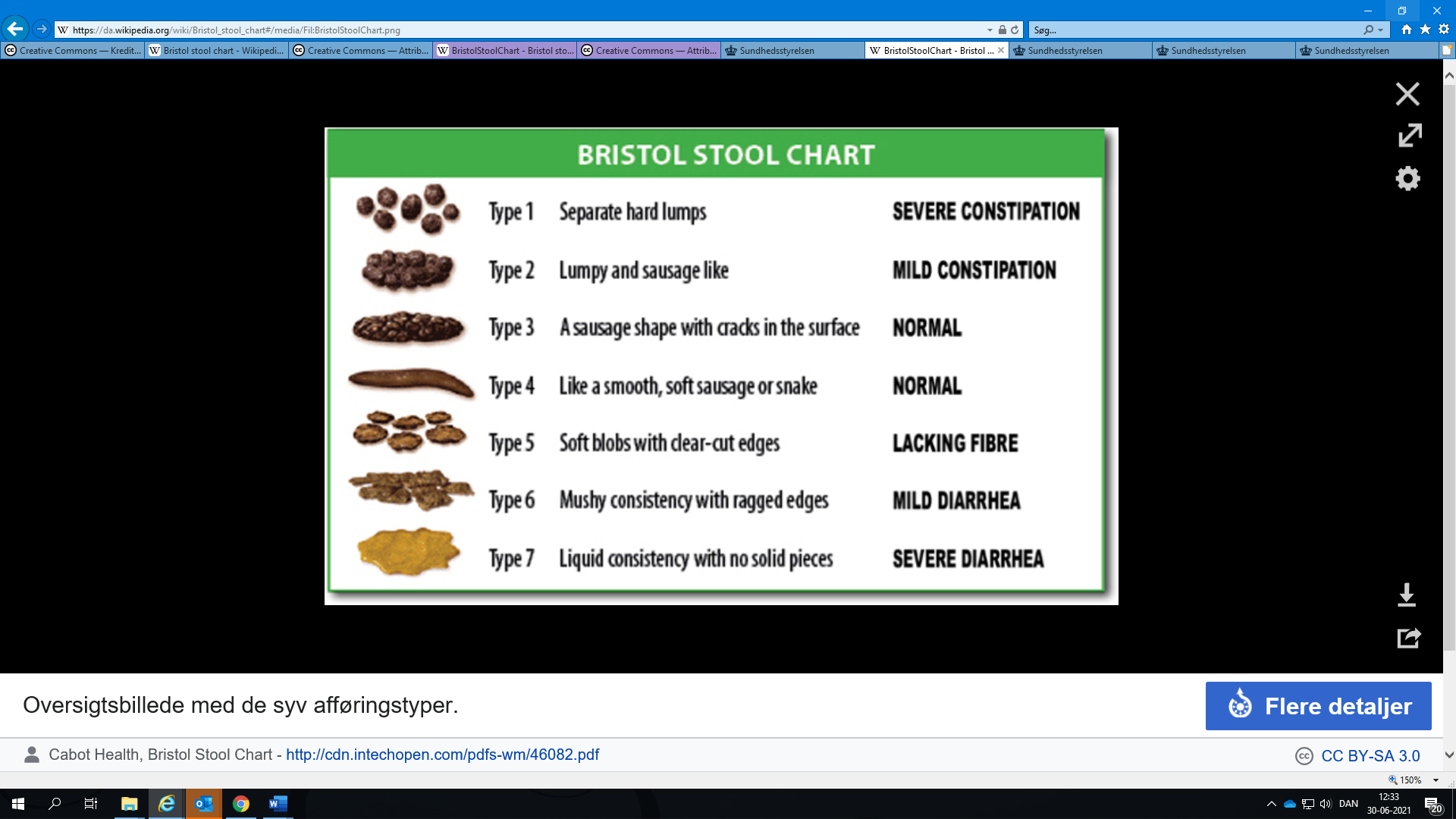  Type 5 Soft blobs  (clear cut edges) |  |  |  |  |  |  |  |  |
| 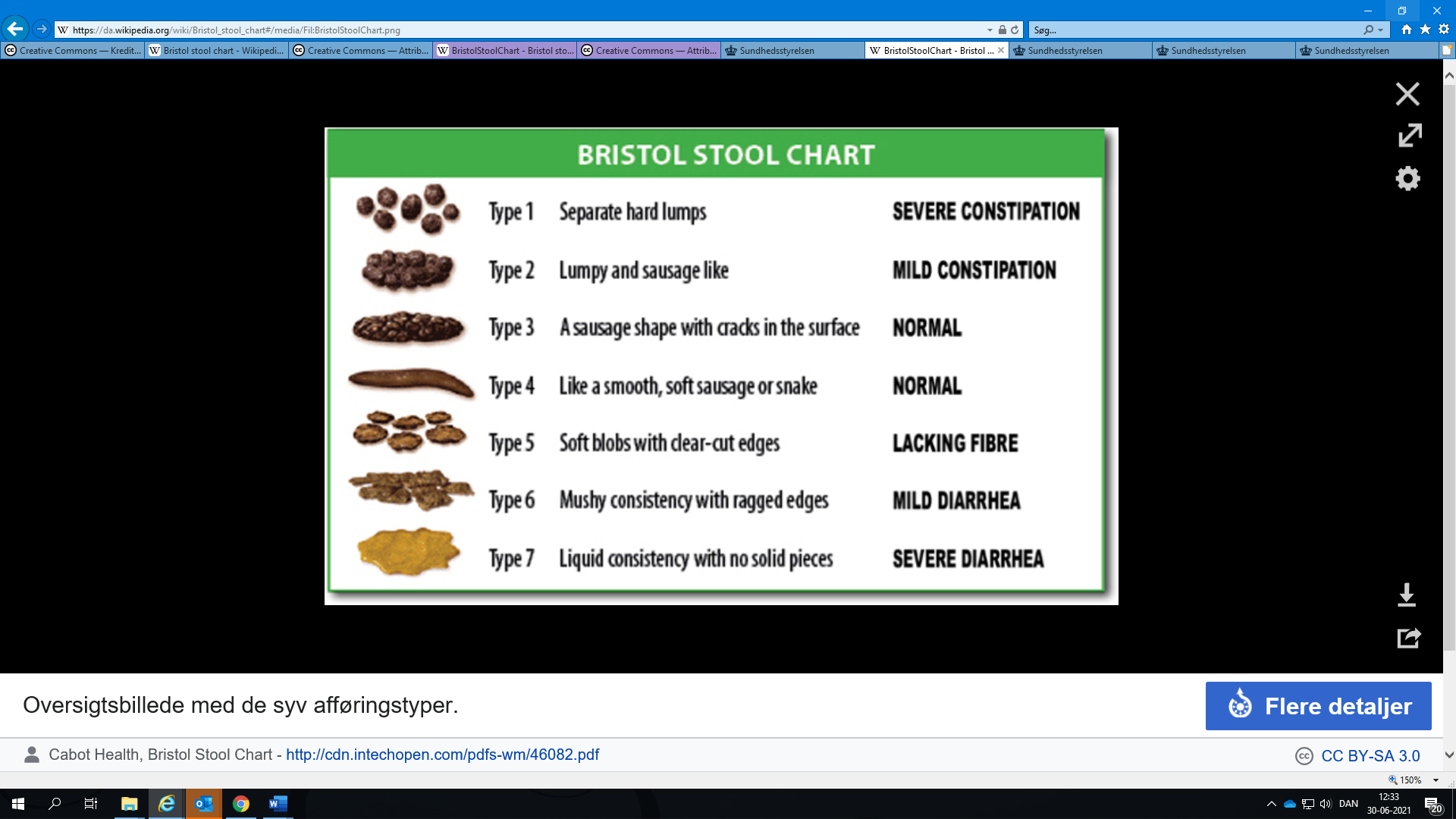 Mushy stool  Type 6 (fluffy small pieces,  ragged edged) |  |  |  |  |  |  |  |  |
| 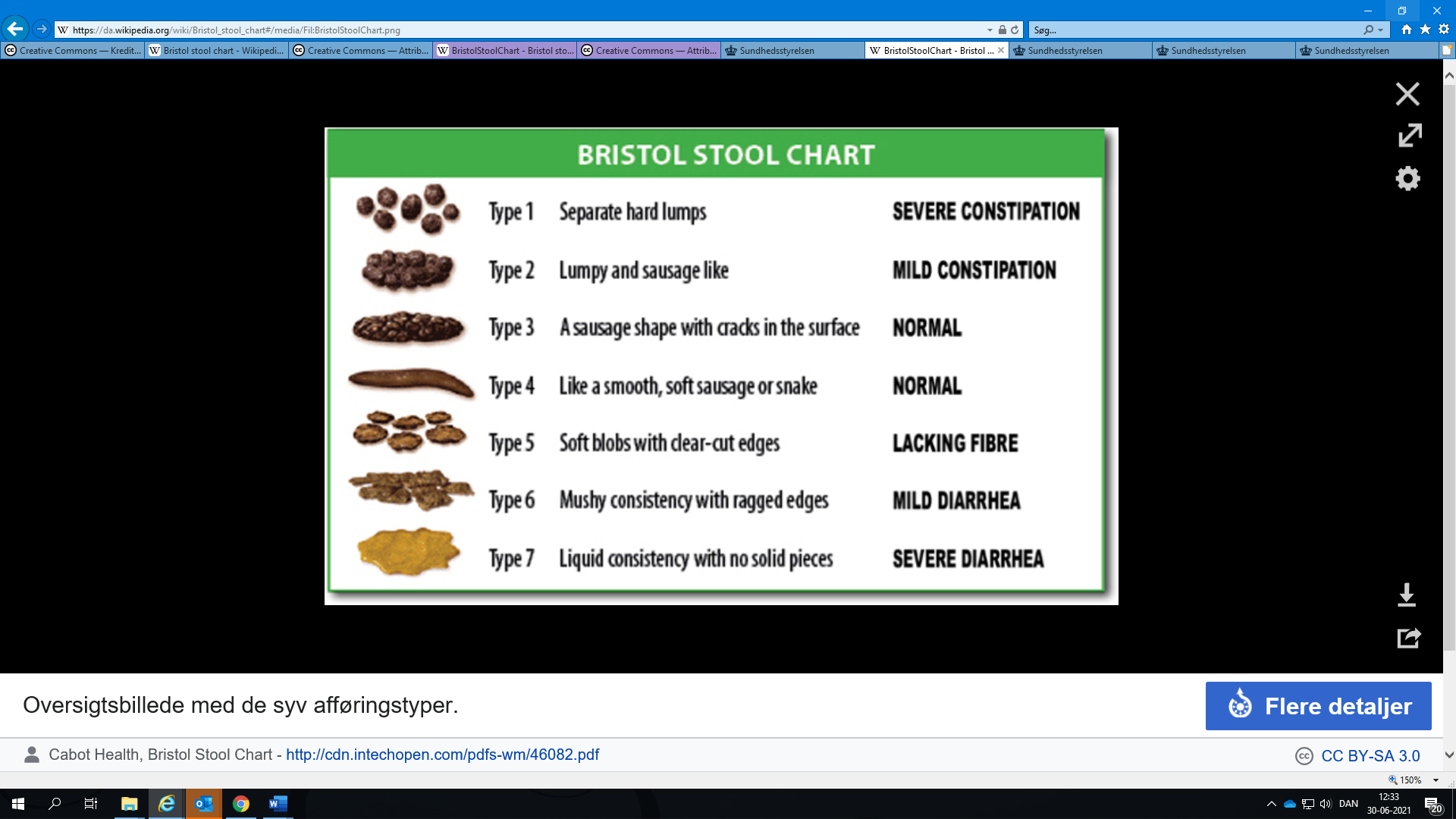  Type 7 Watery  (no solid pieces) |  |  |  |  |  |  |  |  |

**Week 7**

| **Diary Bristol scale for stool types** | | | | | | | | **Comments** |
| --- | --- | --- | --- | --- | --- | --- | --- | --- |
| **Date** |  |  |  |  |  |  |  |  |
| **Body weight** | **kg** |  |  |  |  |  |  |  |
| **Day of the week** | **day** | **day** | **day** | **day** | **day** | **day** | **day** |  |
| 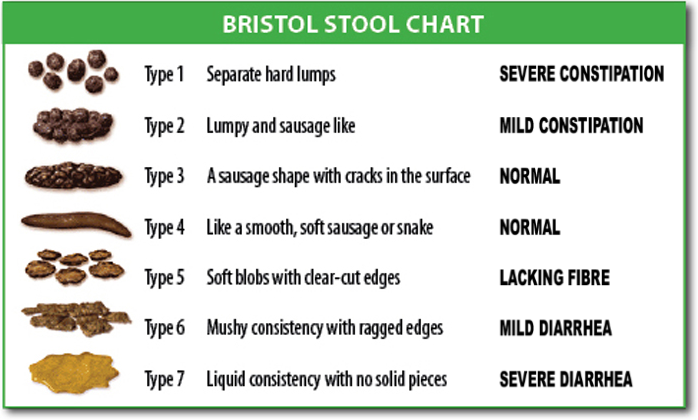  Type 1 Hard lumps |  |  |  |  |  |  |  |  |
| 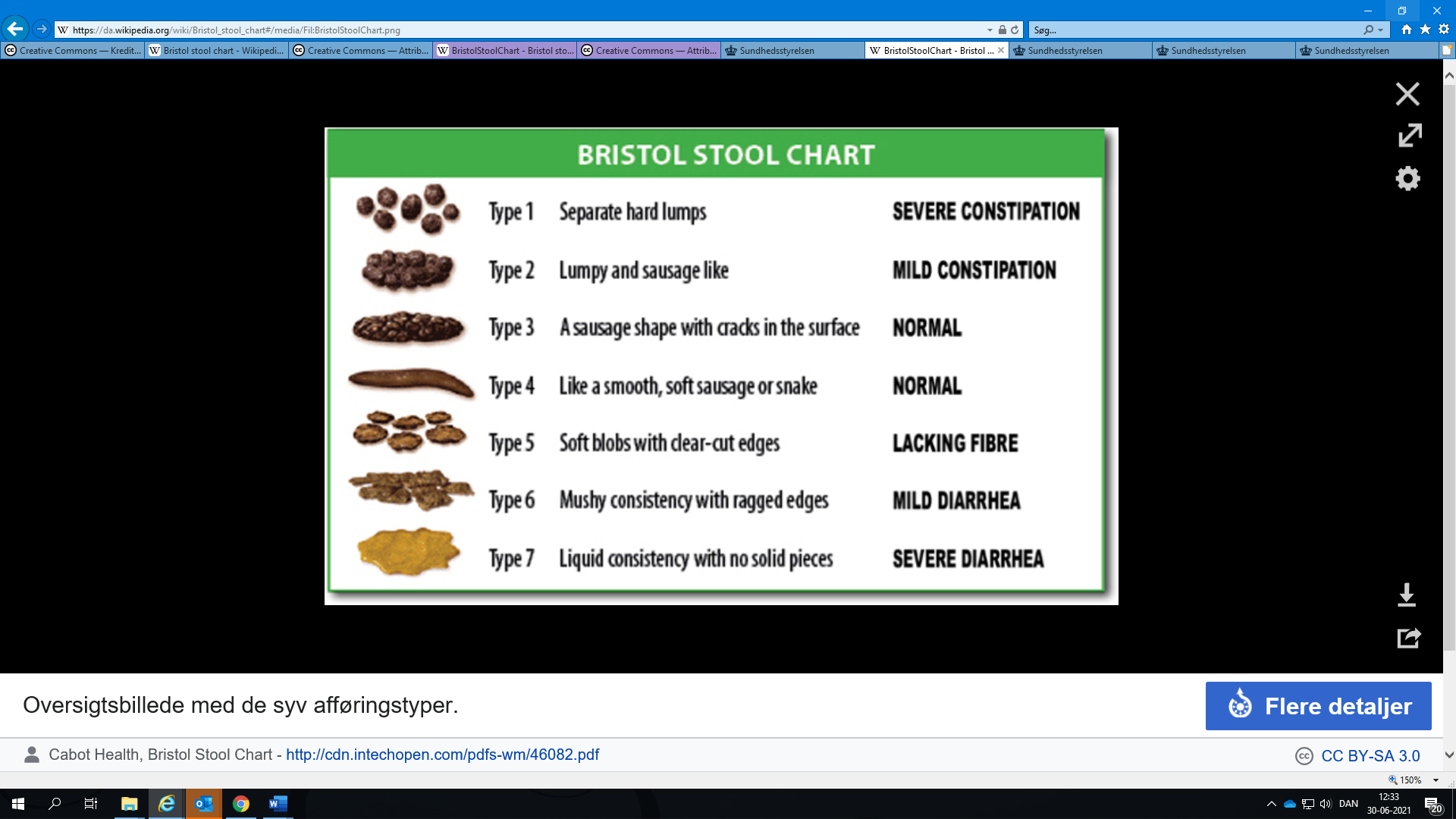  Type 2 Sausage-shaped  (lumpy surface) |  |  |  |  |  |  |  |  |
| 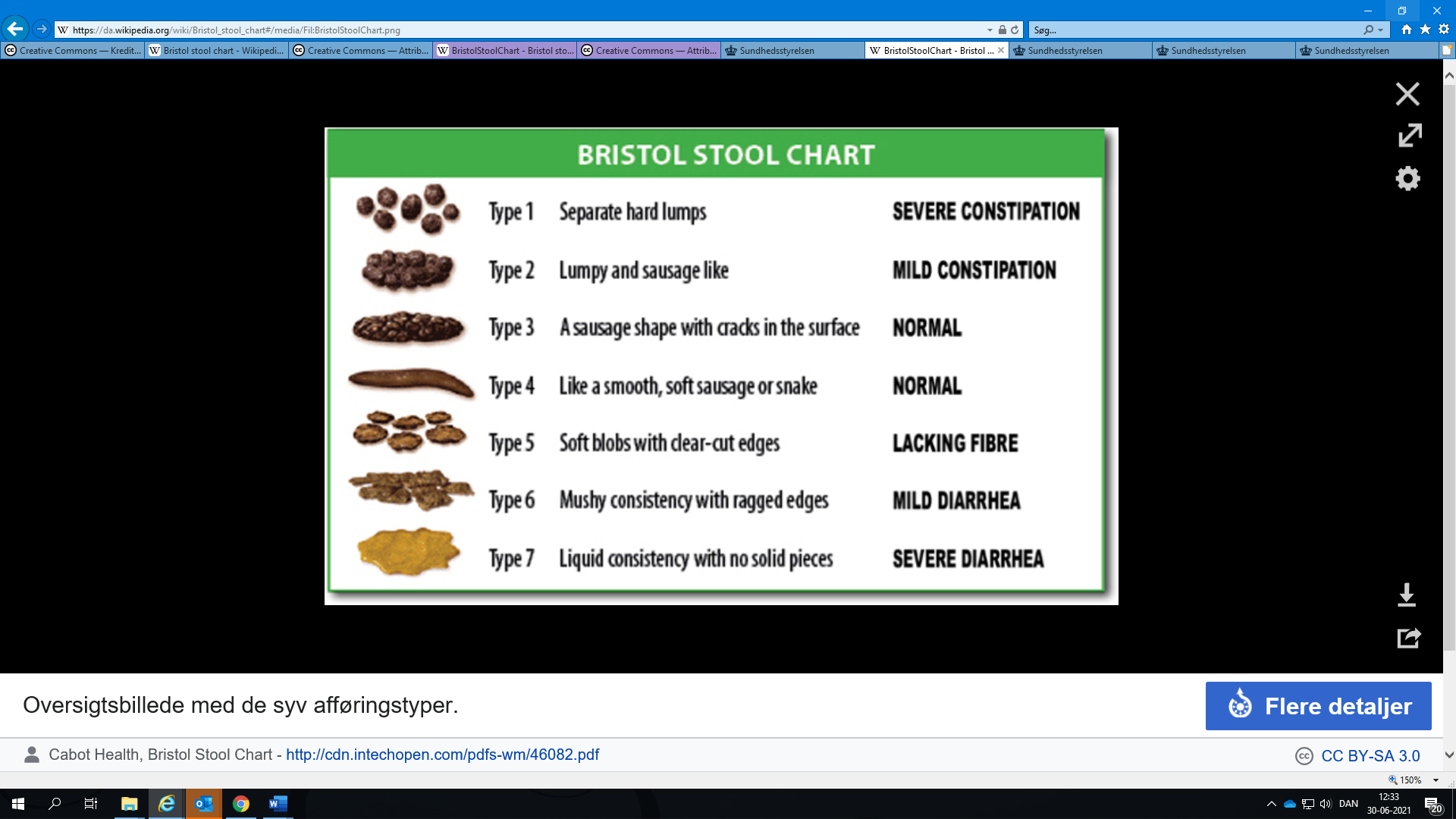  Type 3 Sausage-shaped  (cracked) |  |  |  |  |  |  |  |  |
| 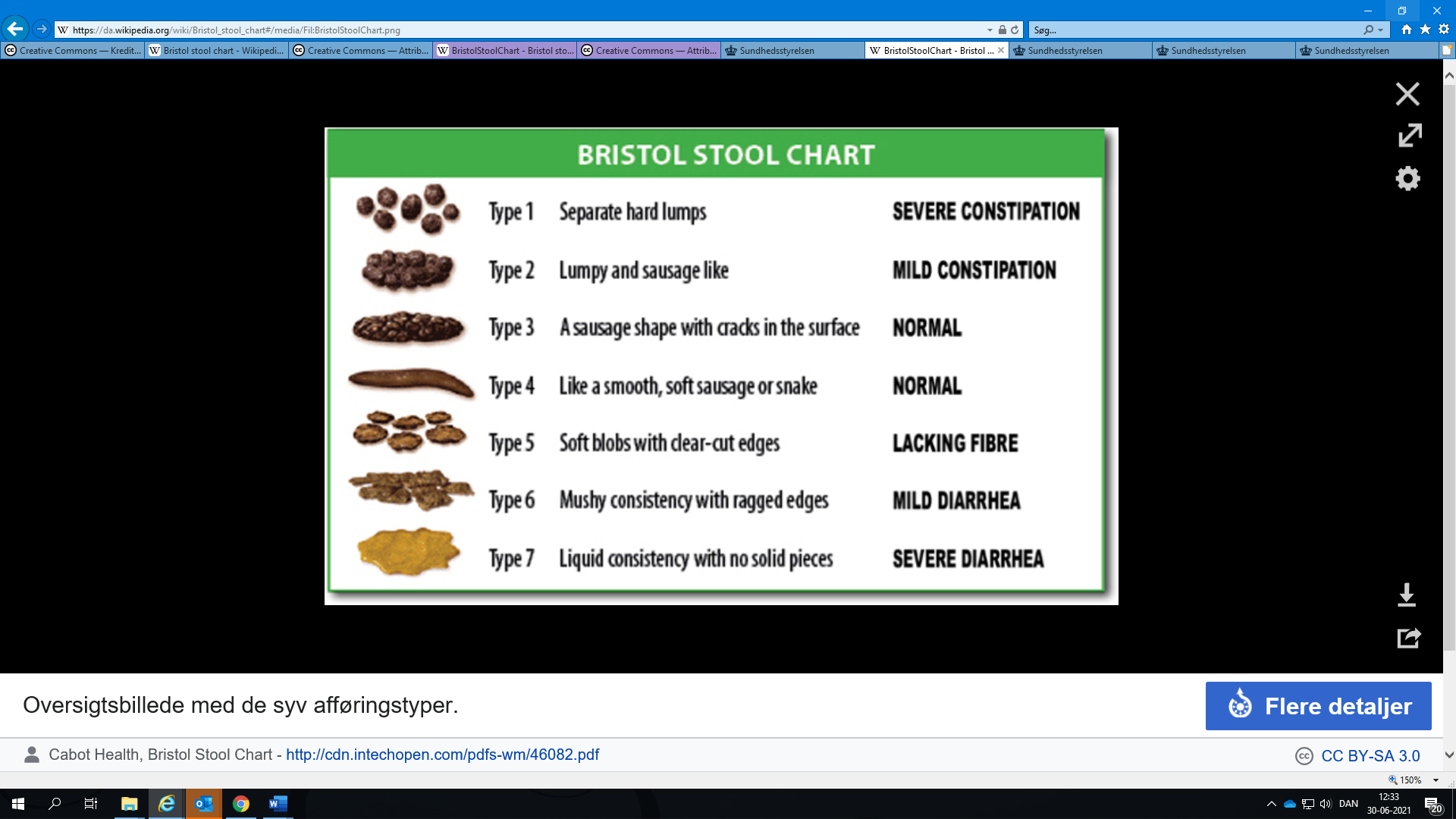  Type 4 Sausage or snake-shaped  (smooth and soft) |  |  |  |  |  |  |  |  |
| 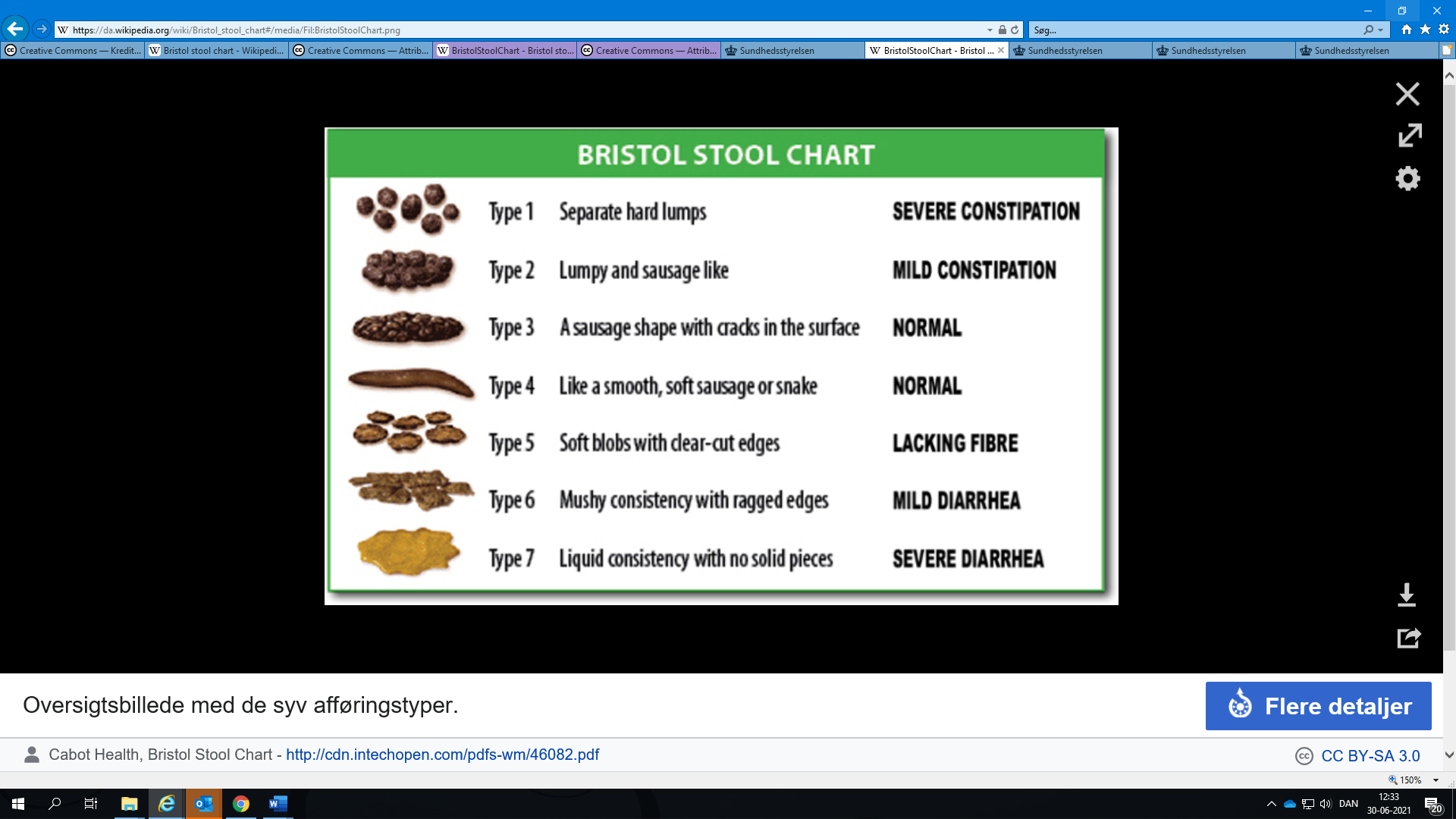  Type 5 Soft blobs  (clear cut edges) |  |  |  |  |  |  |  |  |
| 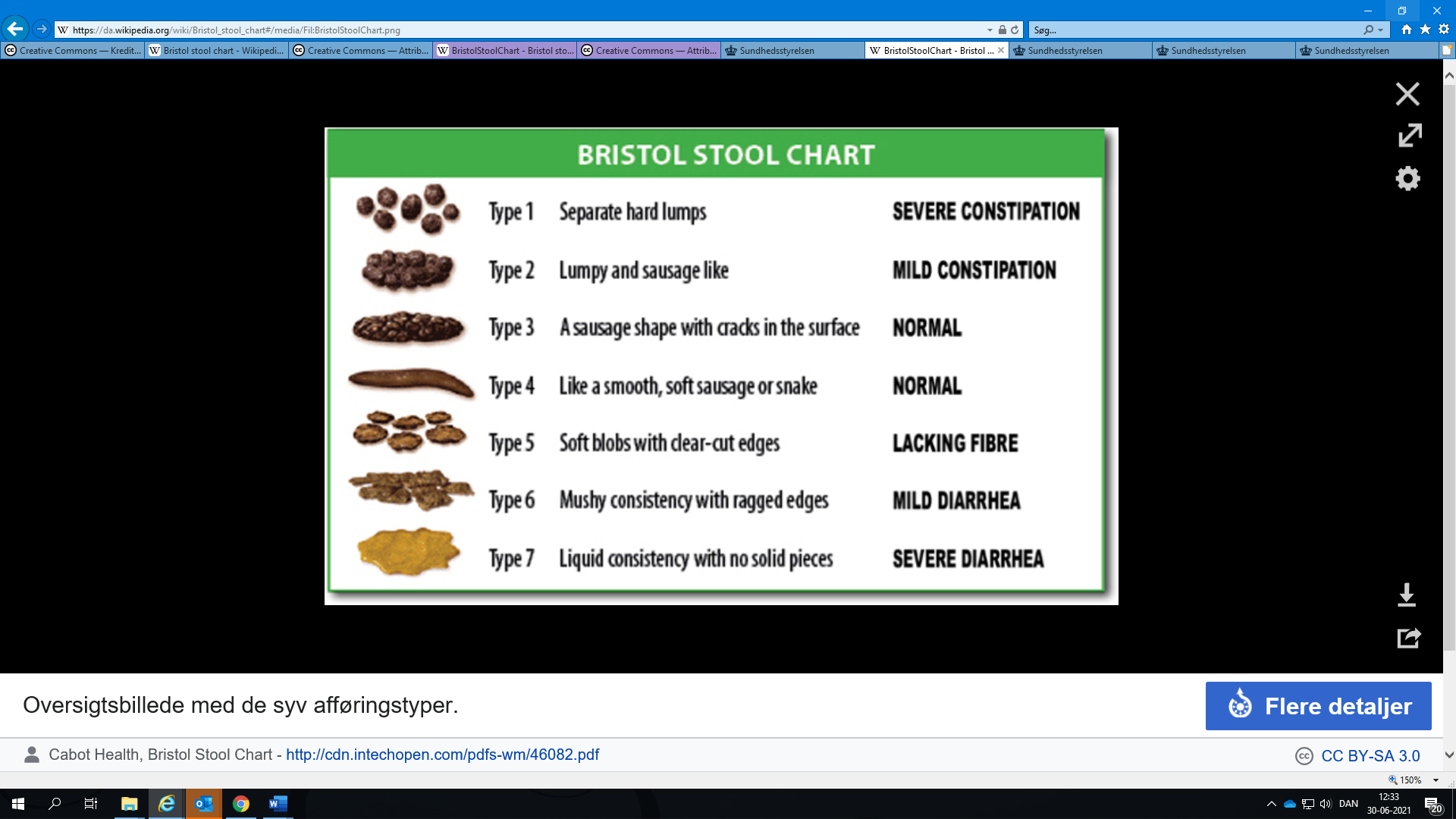 Mushy stool  Type 6 (fluffy small pieces,  ragged edged) |  |  |  |  |  |  |  |  |
| 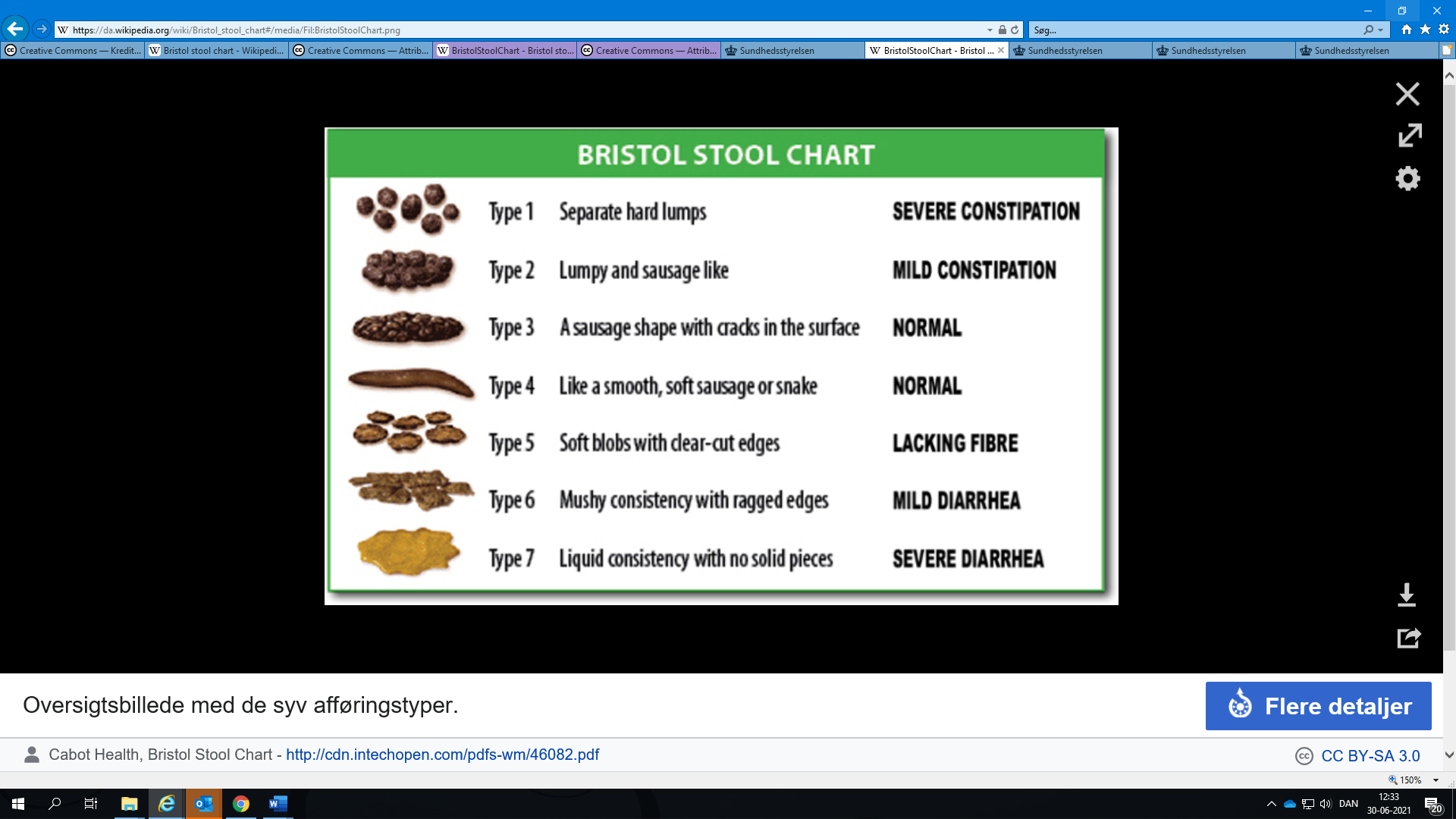  Type 7 Watery  (no solid pieces) |  |  |  |  |  |  |  |  |

**Week 8**

| **Diary Bristol scale for stool types** | | | | | | | | **Comments** |
| --- | --- | --- | --- | --- | --- | --- | --- | --- |
| **Date** |  |  |  |  |  |  |  |  |
| **Body weight** | **kg** |  |  |  |  |  |  |  |
| **Day of the week** | **day** | **day** | **day** | **day** | **day** | **day** | **day** |  |
| 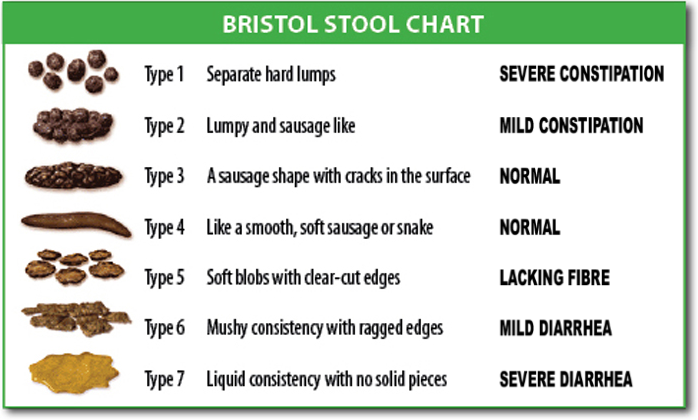  Type 1 Hard lumps |  |  |  |  |  |  |  |  |
| 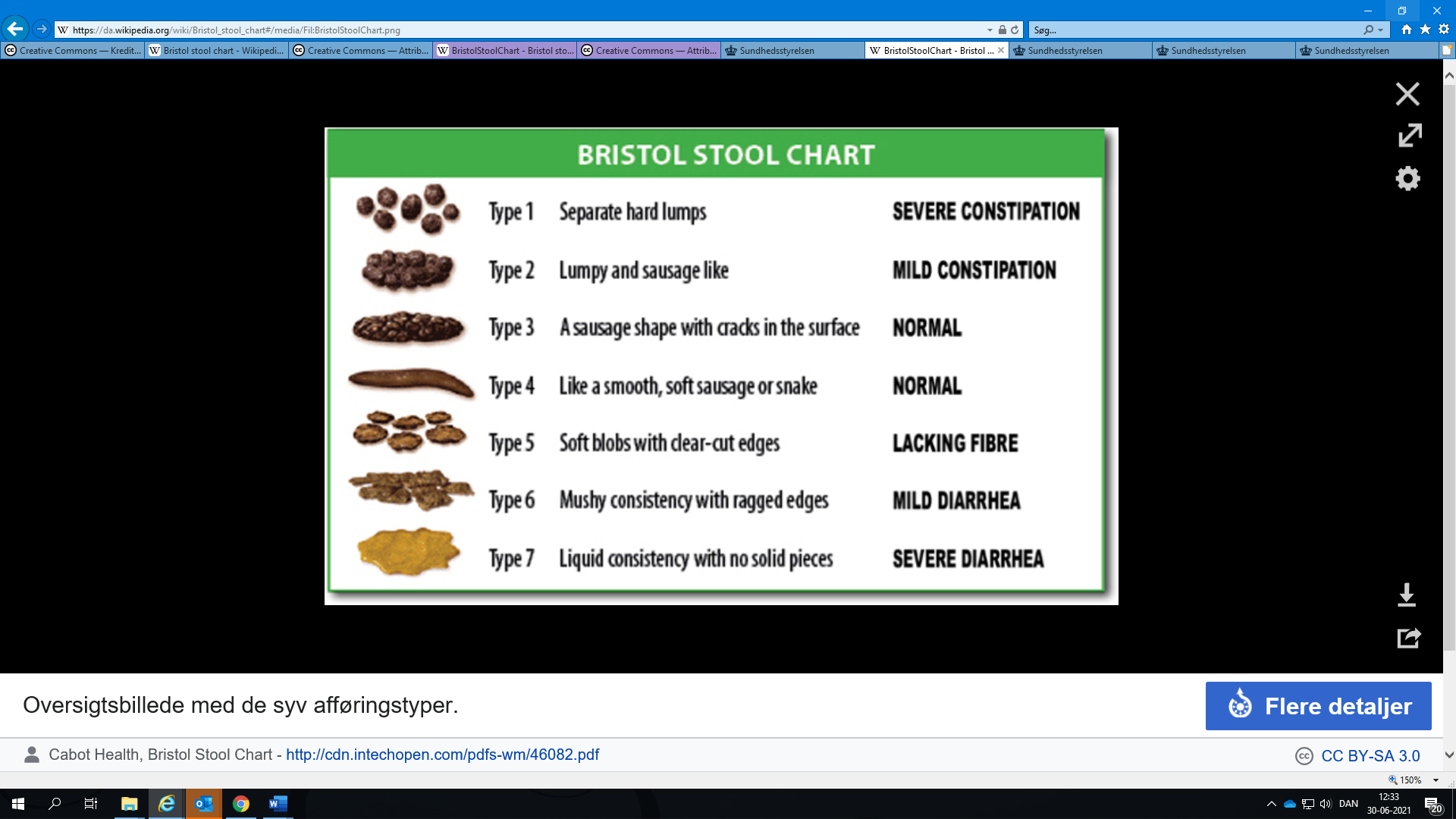  Type 2 Sausage-shaped  (lumpy surface) |  |  |  |  |  |  |  |  |
| 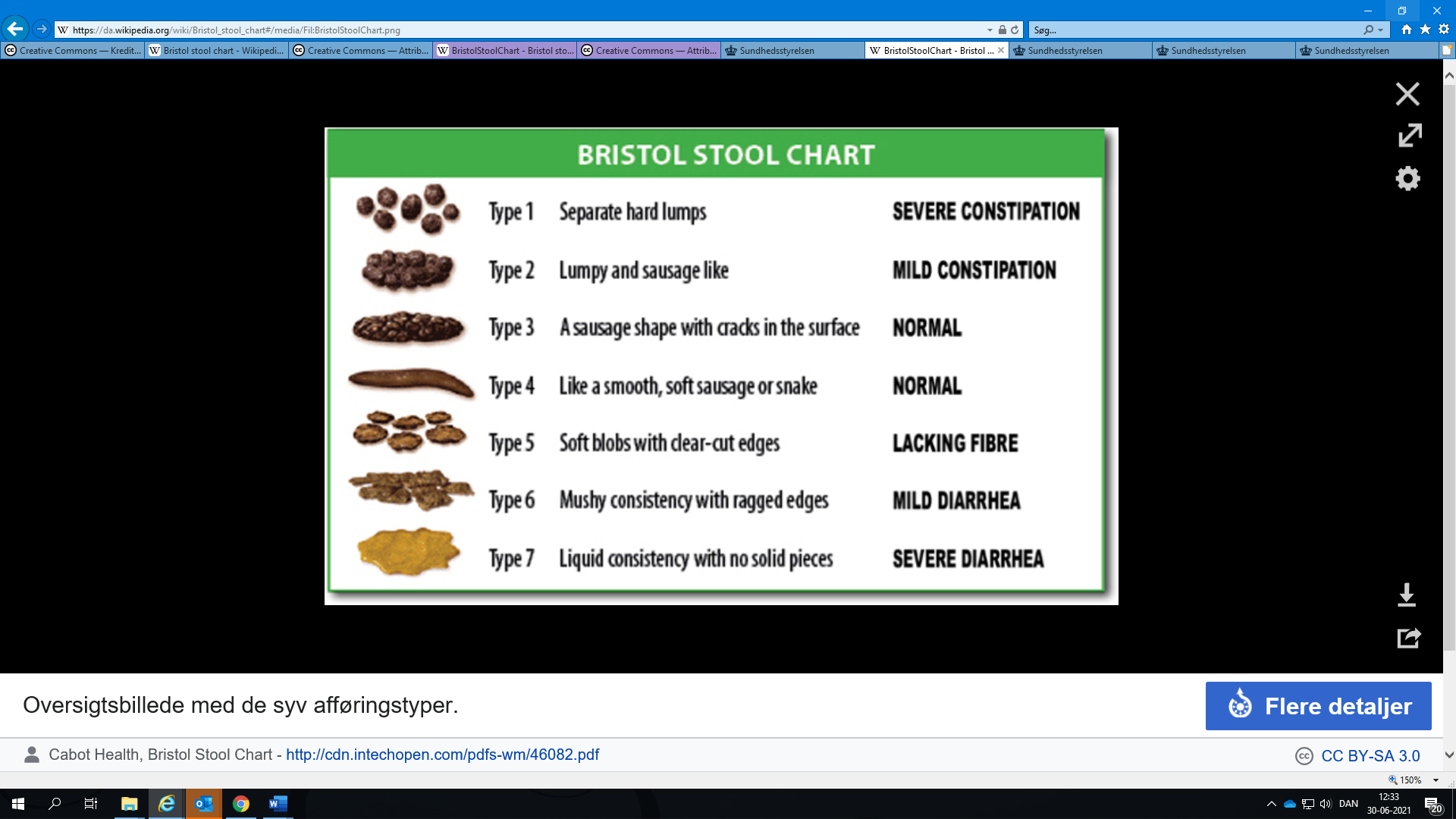  Type 3 Sausage-shaped  (cracked) |  |  |  |  |  |  |  |  |
| 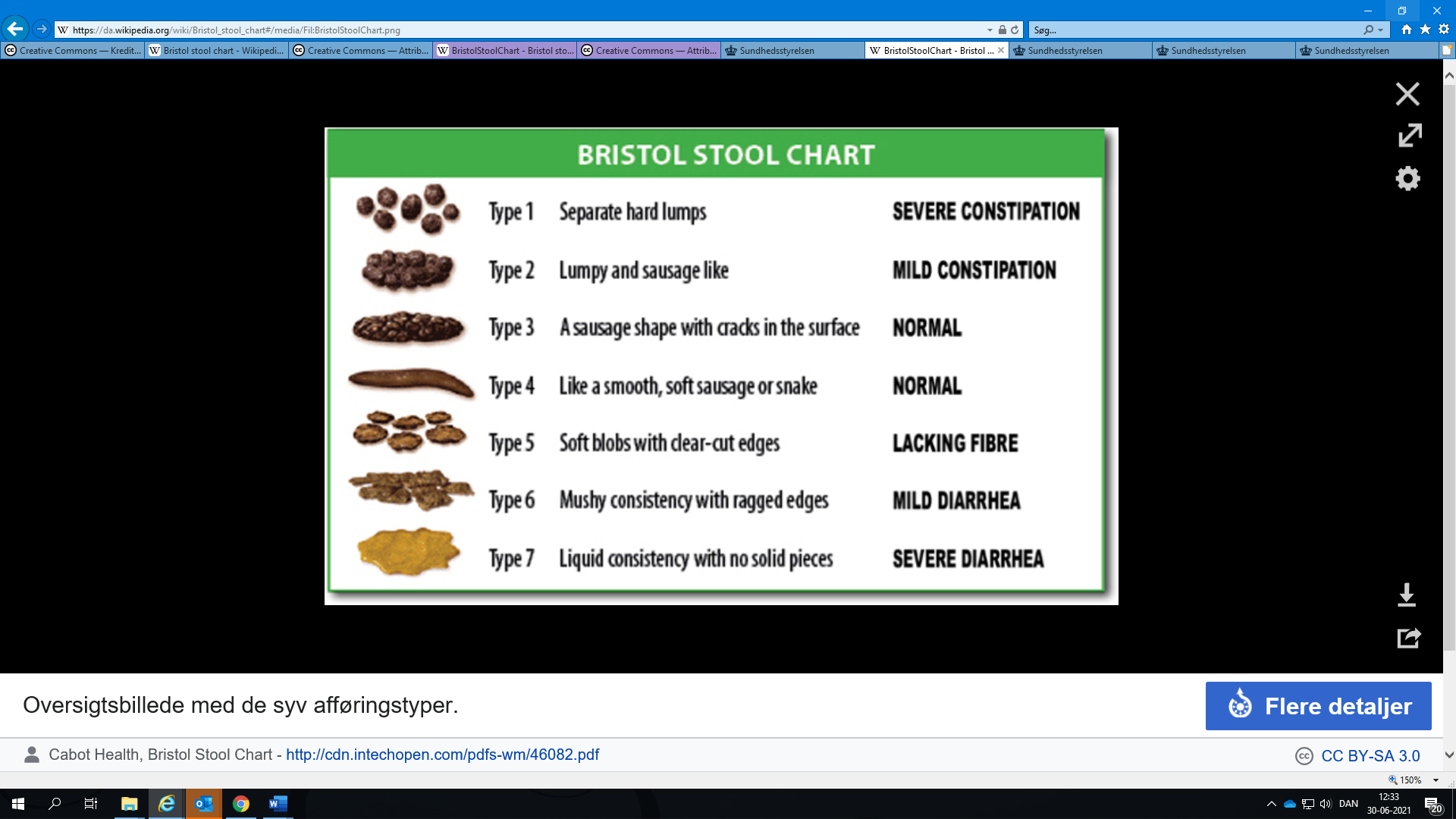  Type 4 Sausage or snake-shaped  (smooth and soft) |  |  |  |  |  |  |  |  |
| 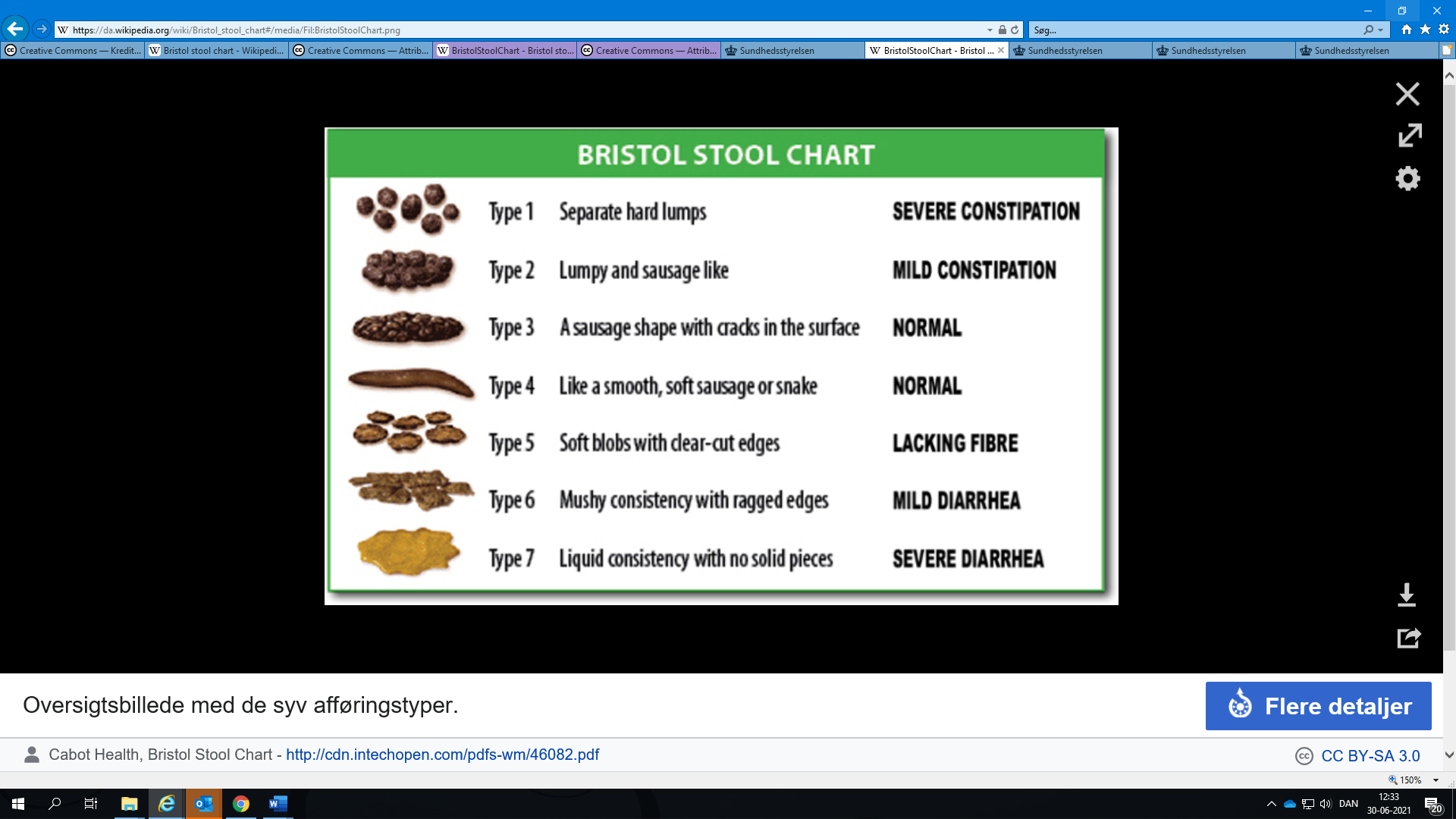  Type 5 Soft blobs  (clear cut edges) |  |  |  |  |  |  |  |  |
| 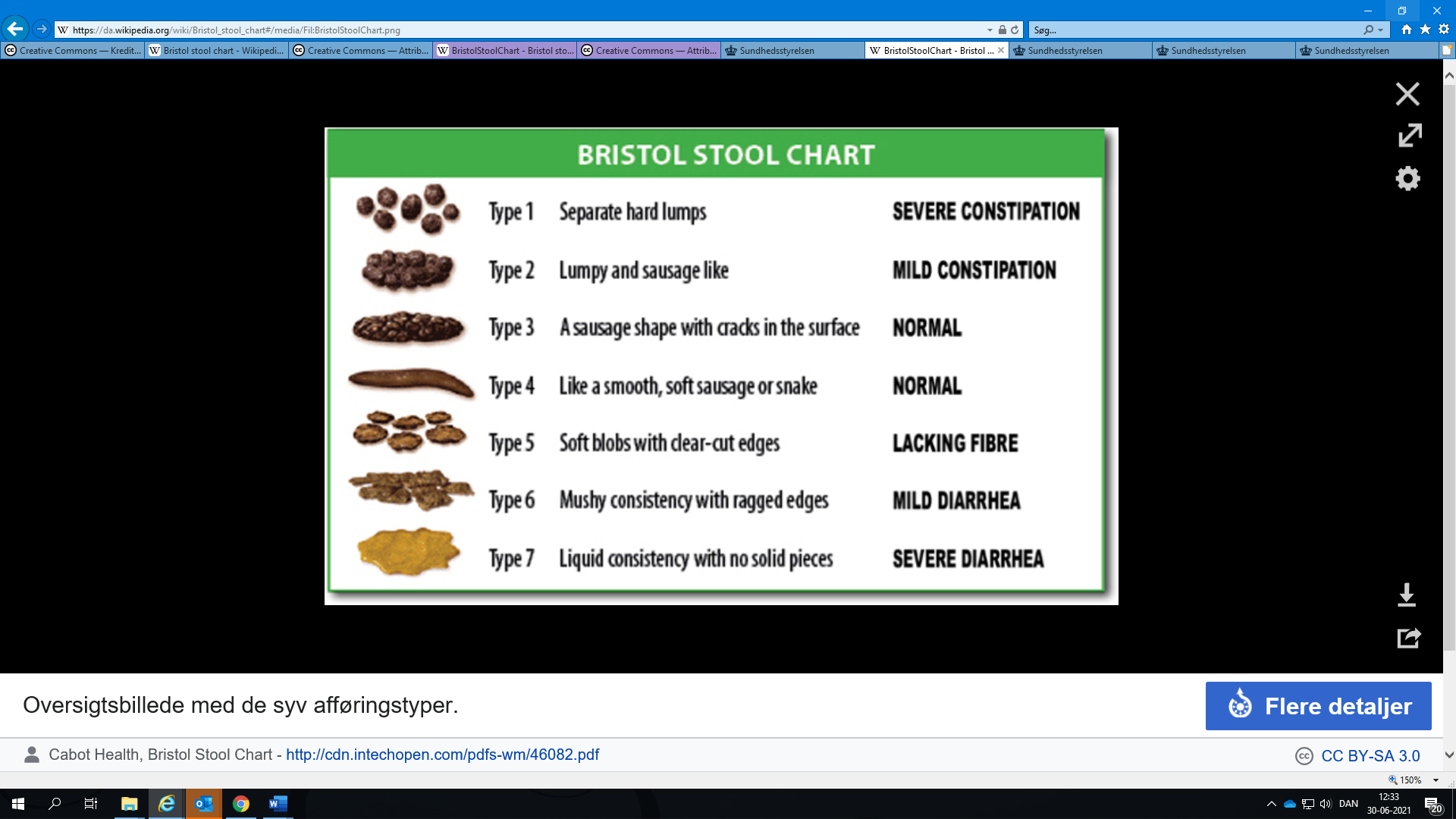 Mushy stool  Type 6 (fluffy small pieces,  ragged edged) |  |  |  |  |  |  |  |  |
| 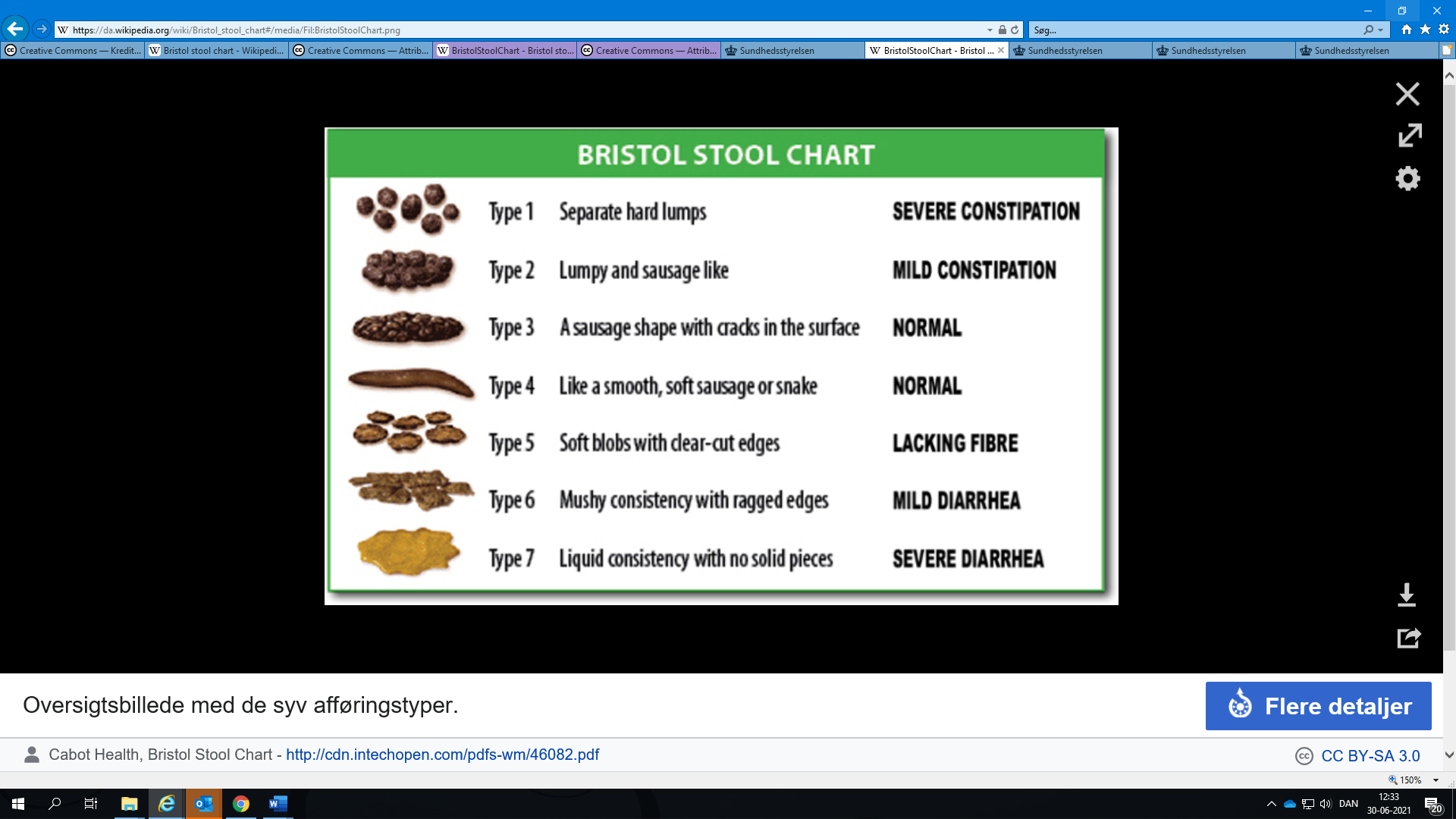  Type 7 Watery  (no solid pieces) |  |  |  |  |  |  |  |  |

**Week 1 after end of radiotherapy**

| **Diary Bristol scale for stool types** | | | | | | | | **Comments** |
| --- | --- | --- | --- | --- | --- | --- | --- | --- |
| **Date** |  |  |  |  |  |  |  |  |
| **Body weight** | **kg** |  |  |  |  |  |  |  |
| **Day of the week** | **day** | **day** | **day** | **day** | **day** | **day** | **day** |  |
| 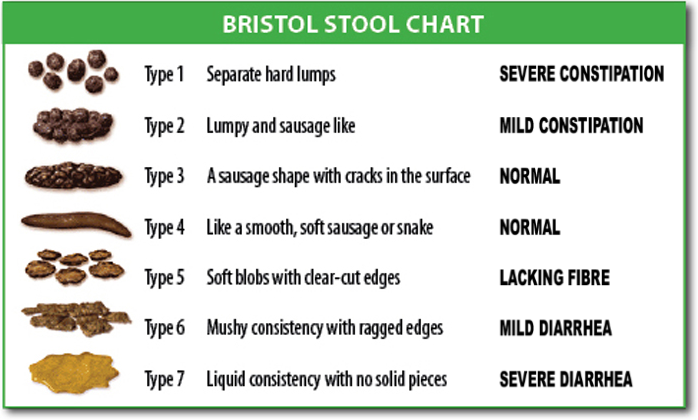  Type 1 Hard lumps |  |  |  |  |  |  |  |  |
| 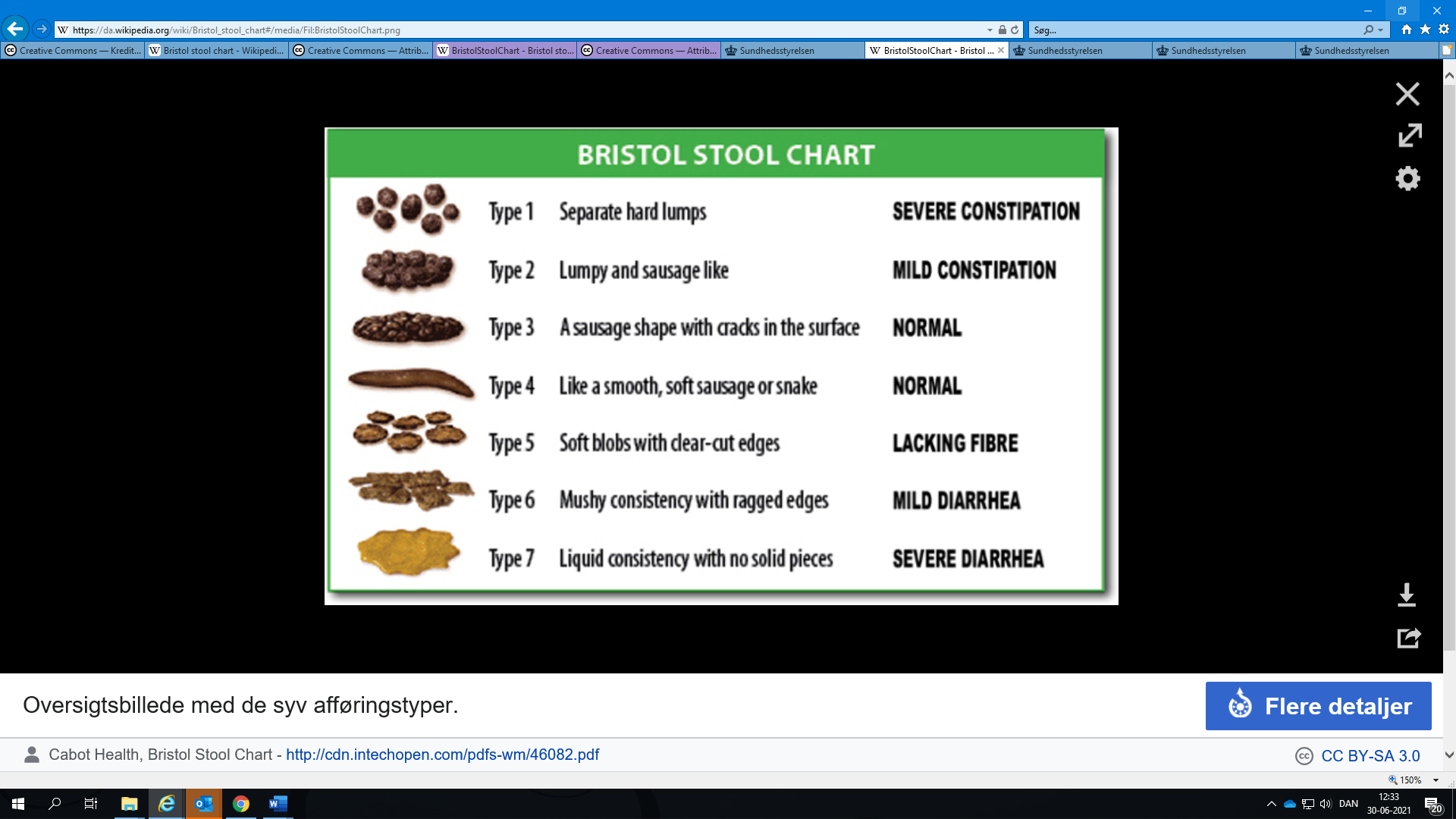  Type 2 Sausage-shaped  (lumpy surface) |  |  |  |  |  |  |  |  |
| 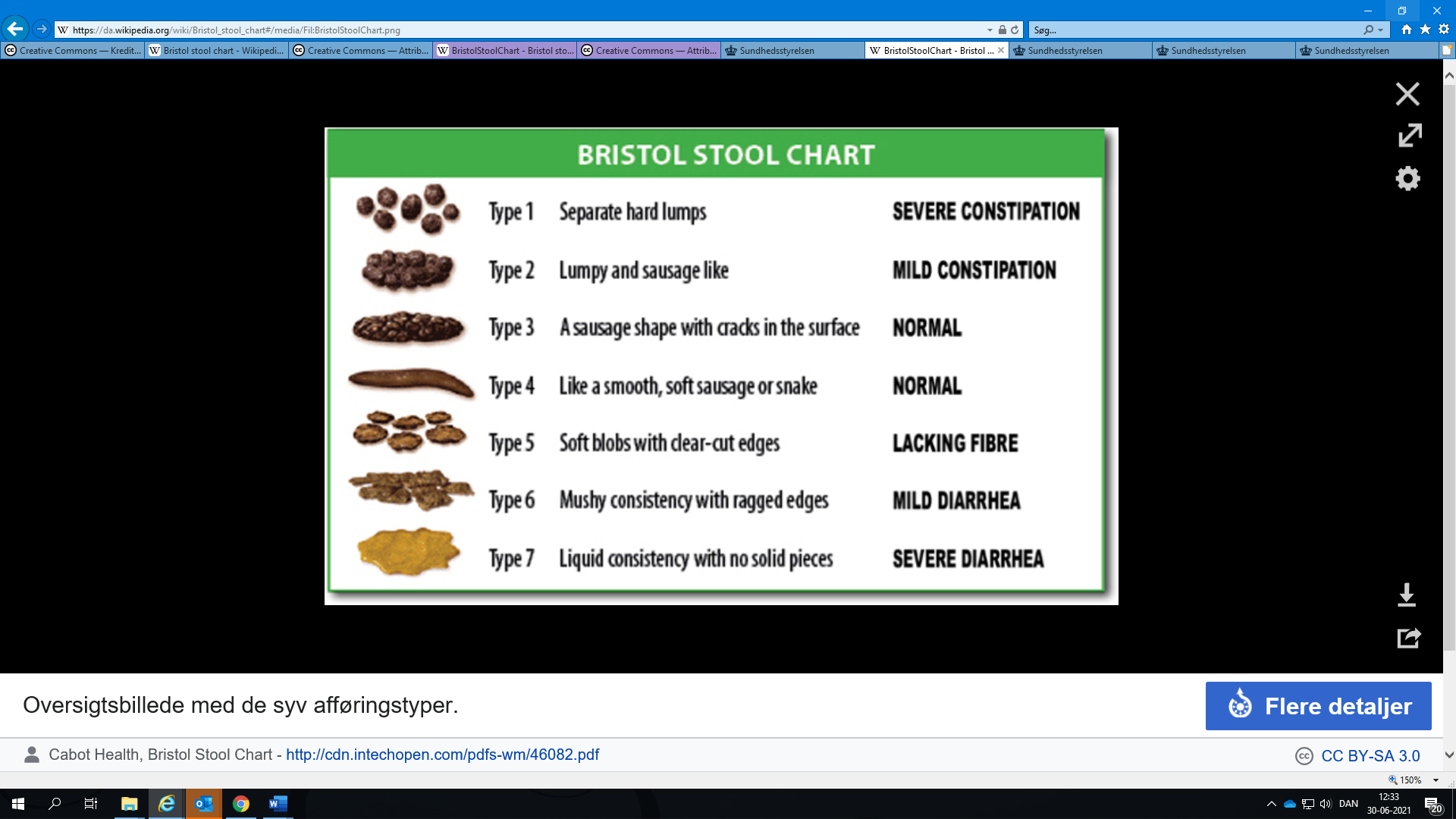  Type 3 Sausage-shaped  (cracked) |  |  |  |  |  |  |  |  |
| 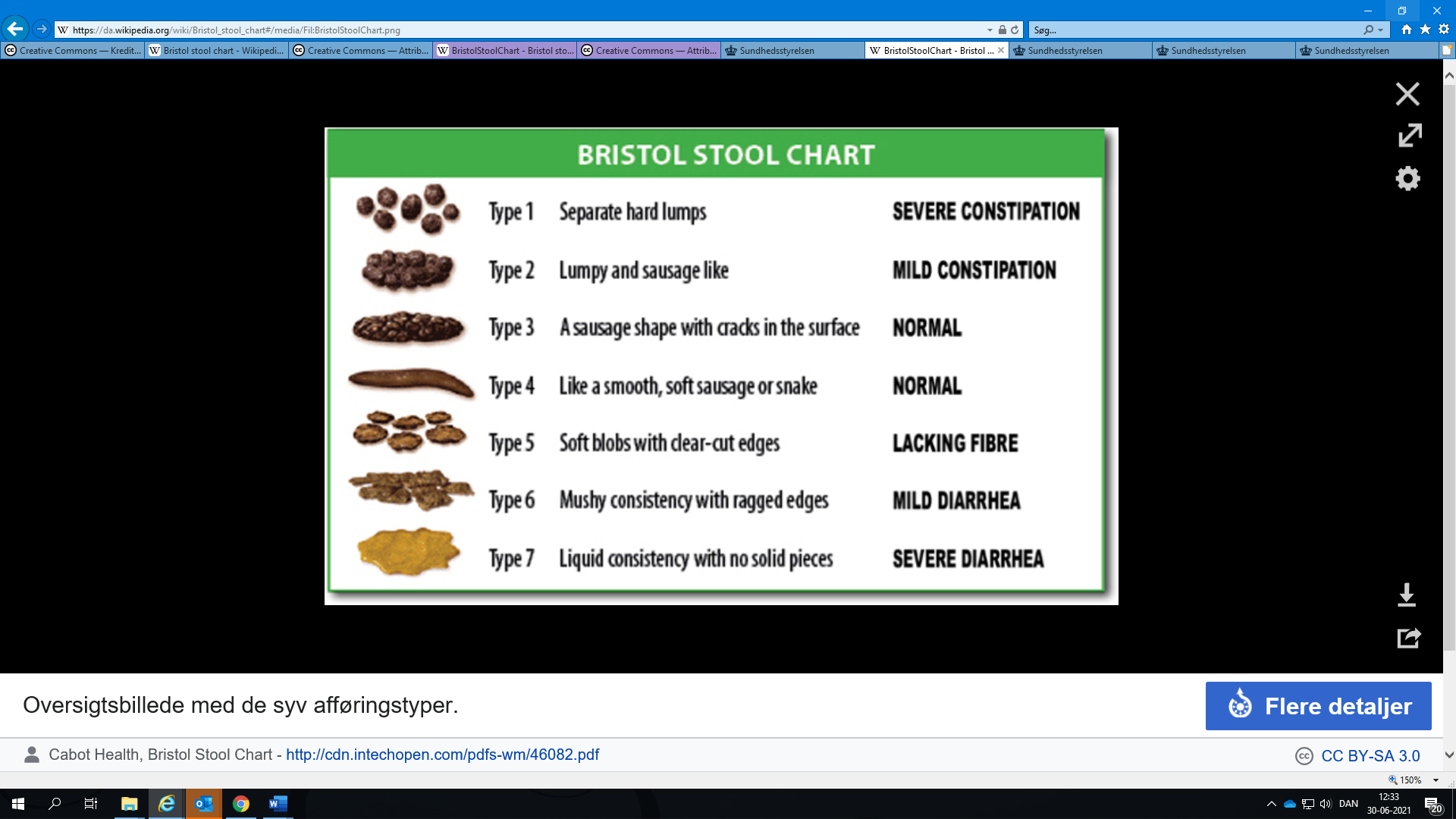  Type 4 Sausage or snake-shaped  (smooth and soft) |  |  |  |  |  |  |  |  |
| 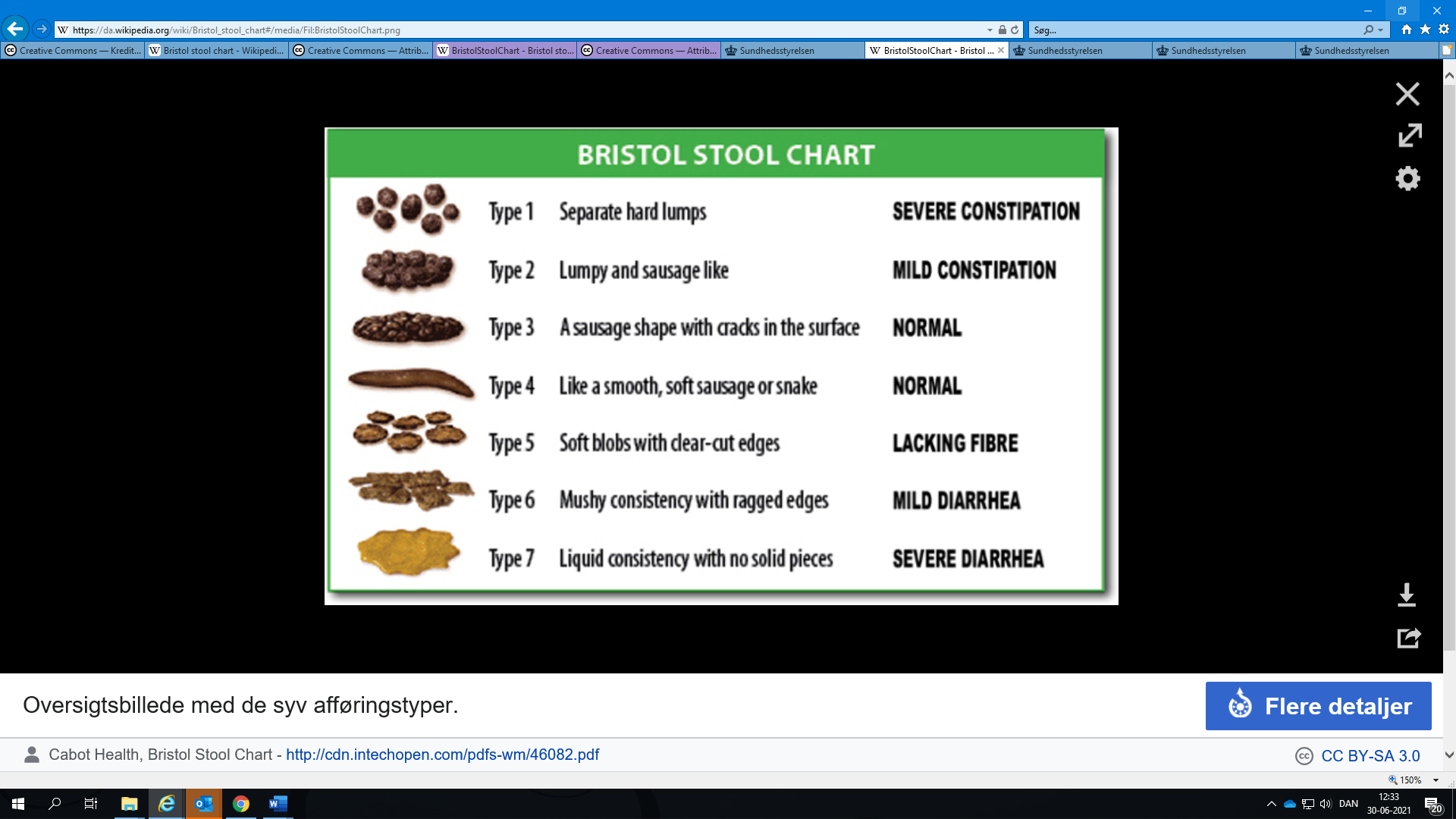  Type 5 Soft blobs  (clear cut edges) |  |  |  |  |  |  |  |  |
| 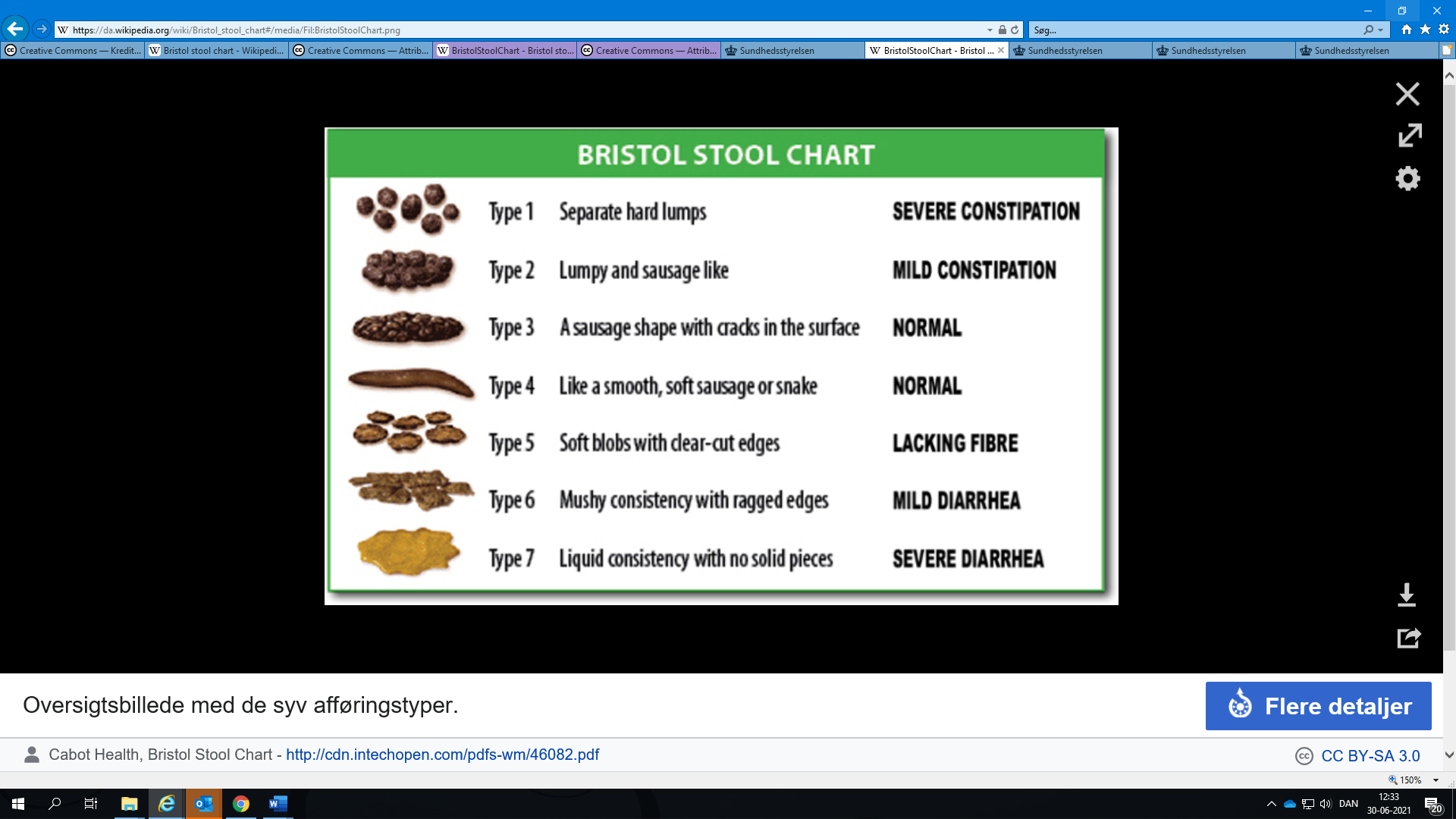 Mushy stool  Type 6 (fluffy small pieces,  ragged edged) |  |  |  |  |  |  |  |  |
| 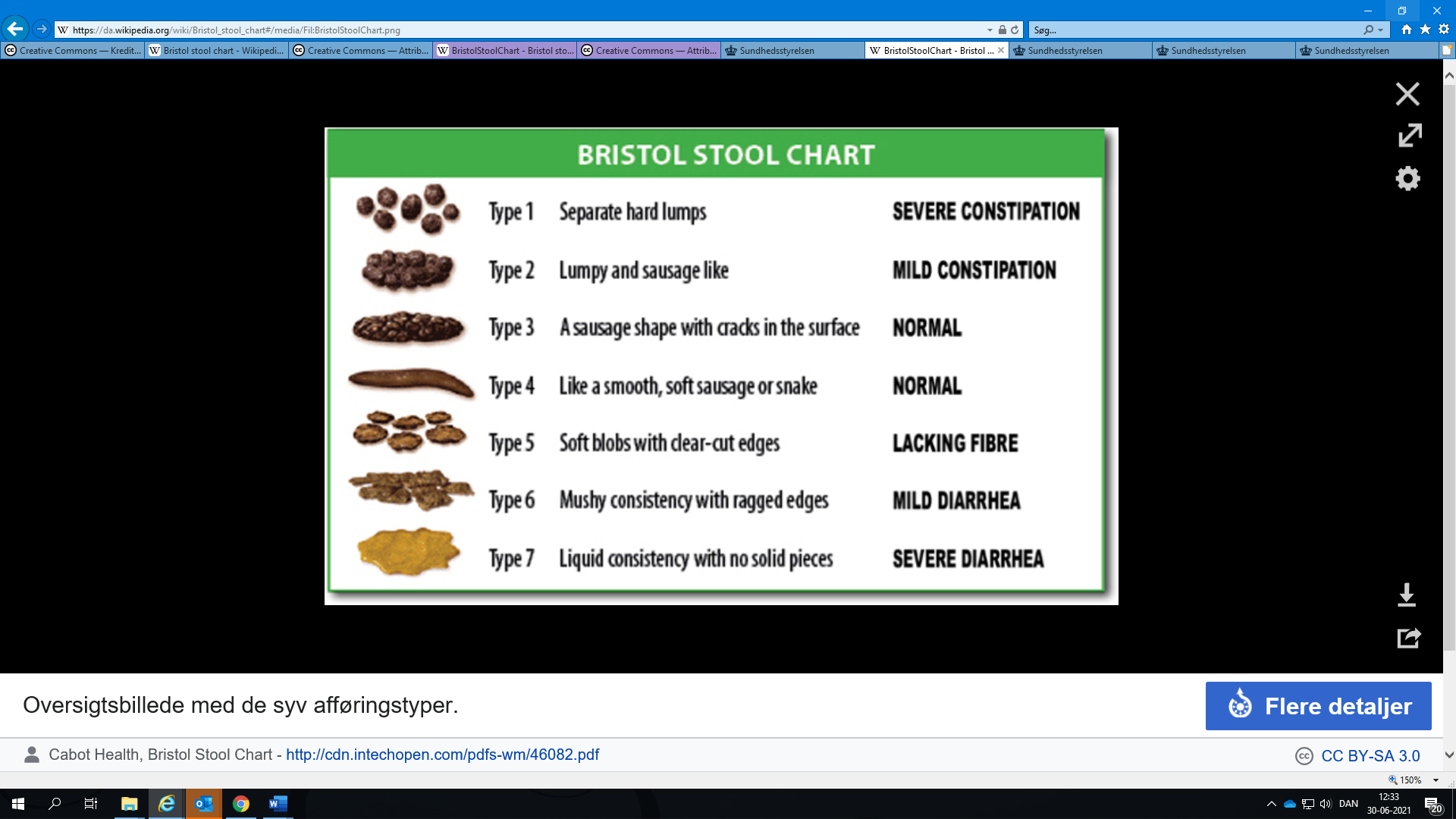  Type 7 Watery  (no solid pieces) |  |  |  |  |  |  |  |  |

**Week 2 after end of radiotherapy**

| **Diary Bristol scale for stool types** | | | | | | | | **Comments** |
| --- | --- | --- | --- | --- | --- | --- | --- | --- |
| **Date** |  |  |  |  |  |  |  |  |
| **Body weight** | **kg** |  |  |  |  |  |  |  |
| **Day of the week** | **day** | **day** | **day** | **day** | **day** | **day** | **day** |  |
| 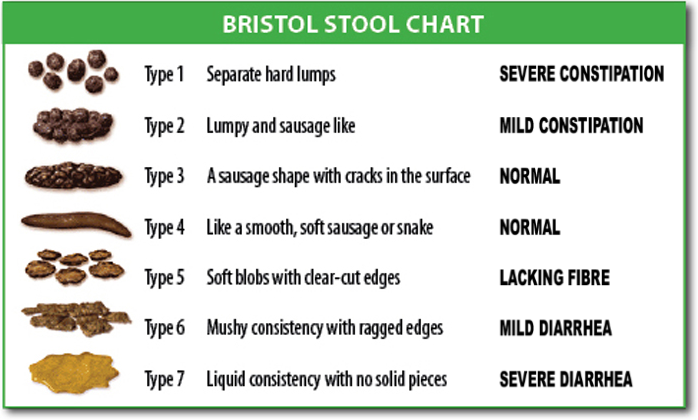  Type 1 Hard lumps |  |  |  |  |  |  |  |  |
| 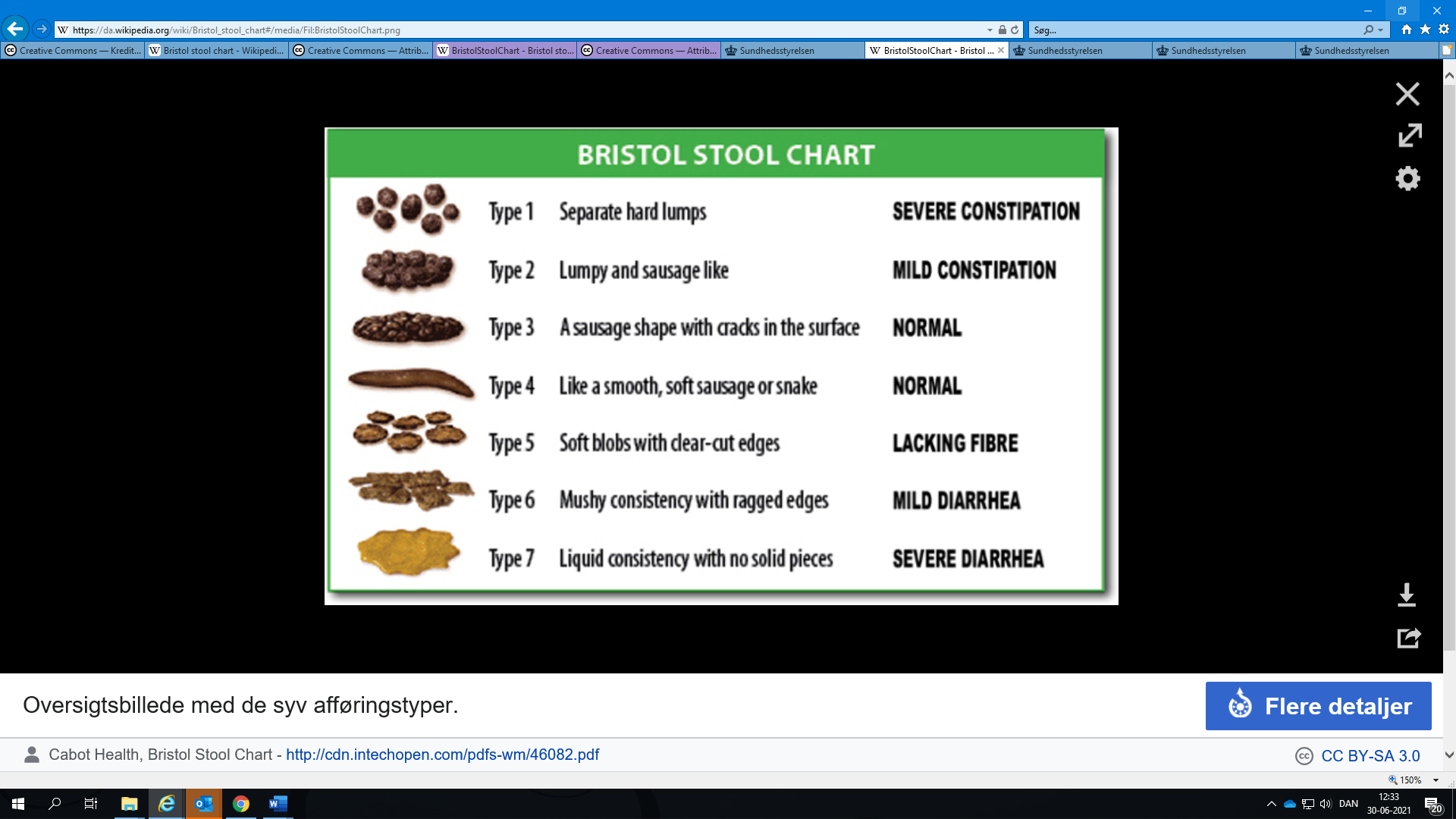  Type 2 Sausage-shaped  (lumpy surface) |  |  |  |  |  |  |  |  |
| 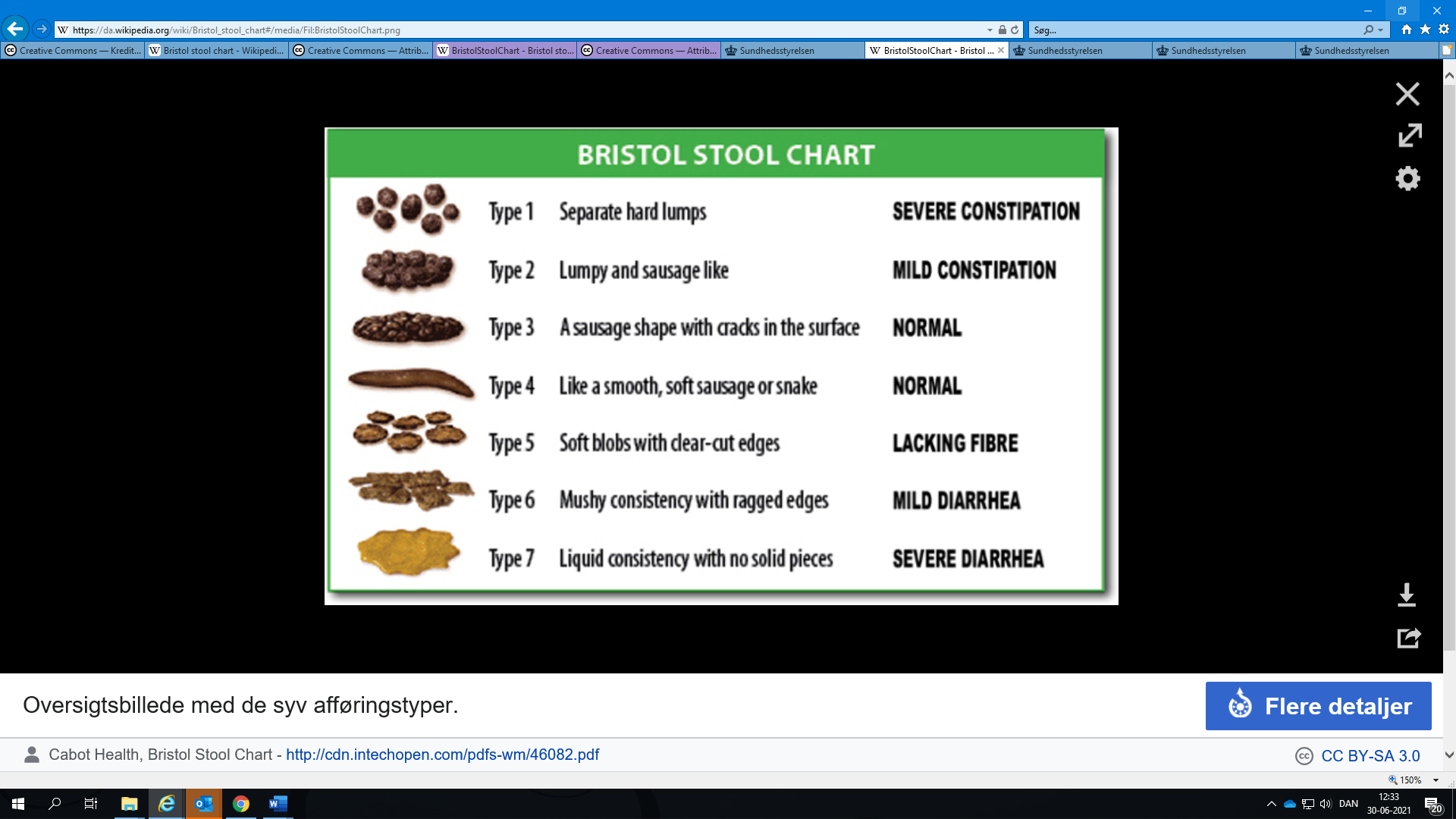  Type 3 Sausage-shaped  (cracked) |  |  |  |  |  |  |  |  |
| 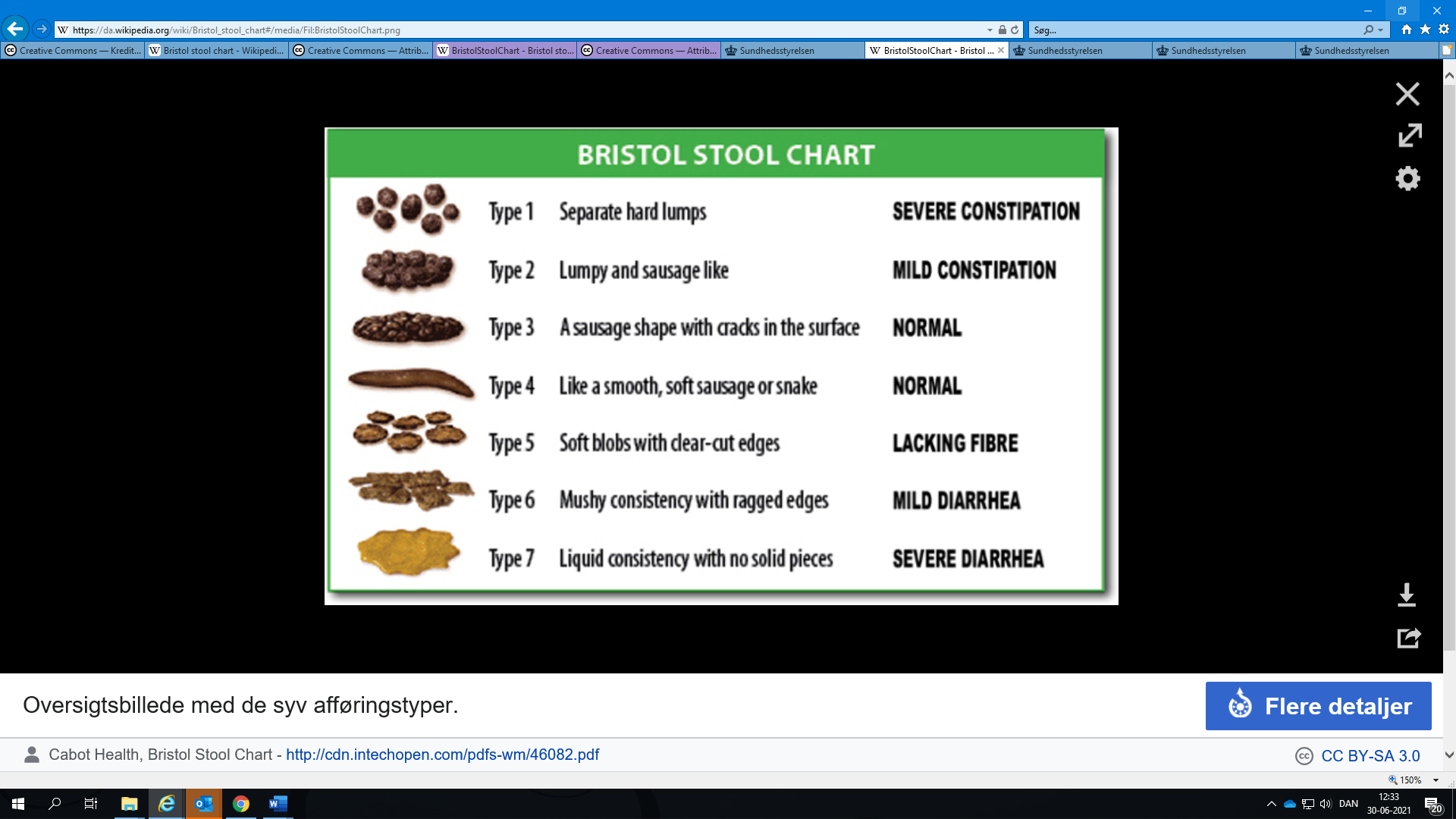  Type 4 Sausage or snake-shaped  (smooth and soft) |  |  |  |  |  |  |  |  |
| 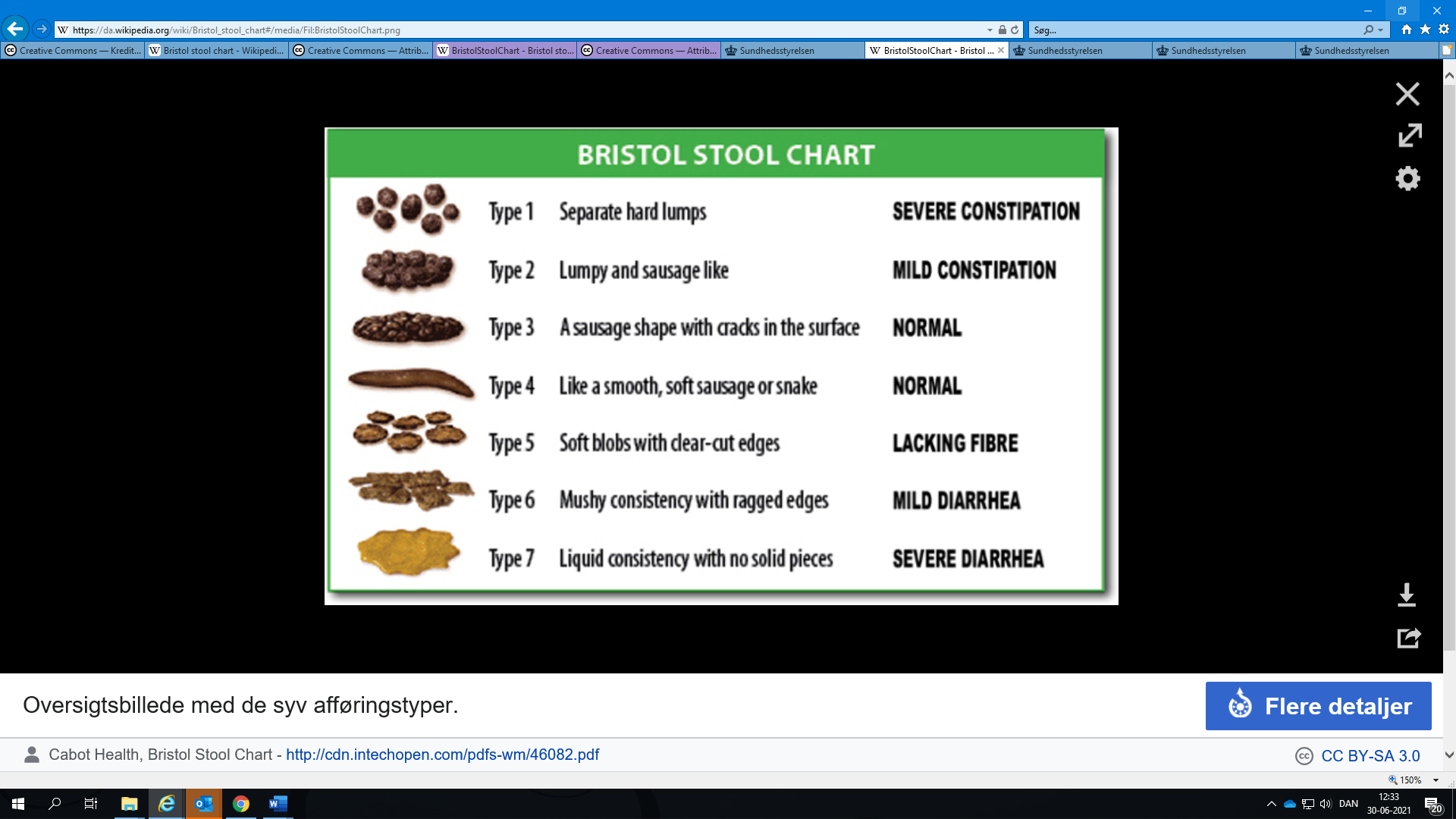  Type 5 Soft blobs  (clear cut edges) |  |  |  |  |  |  |  |  |
| 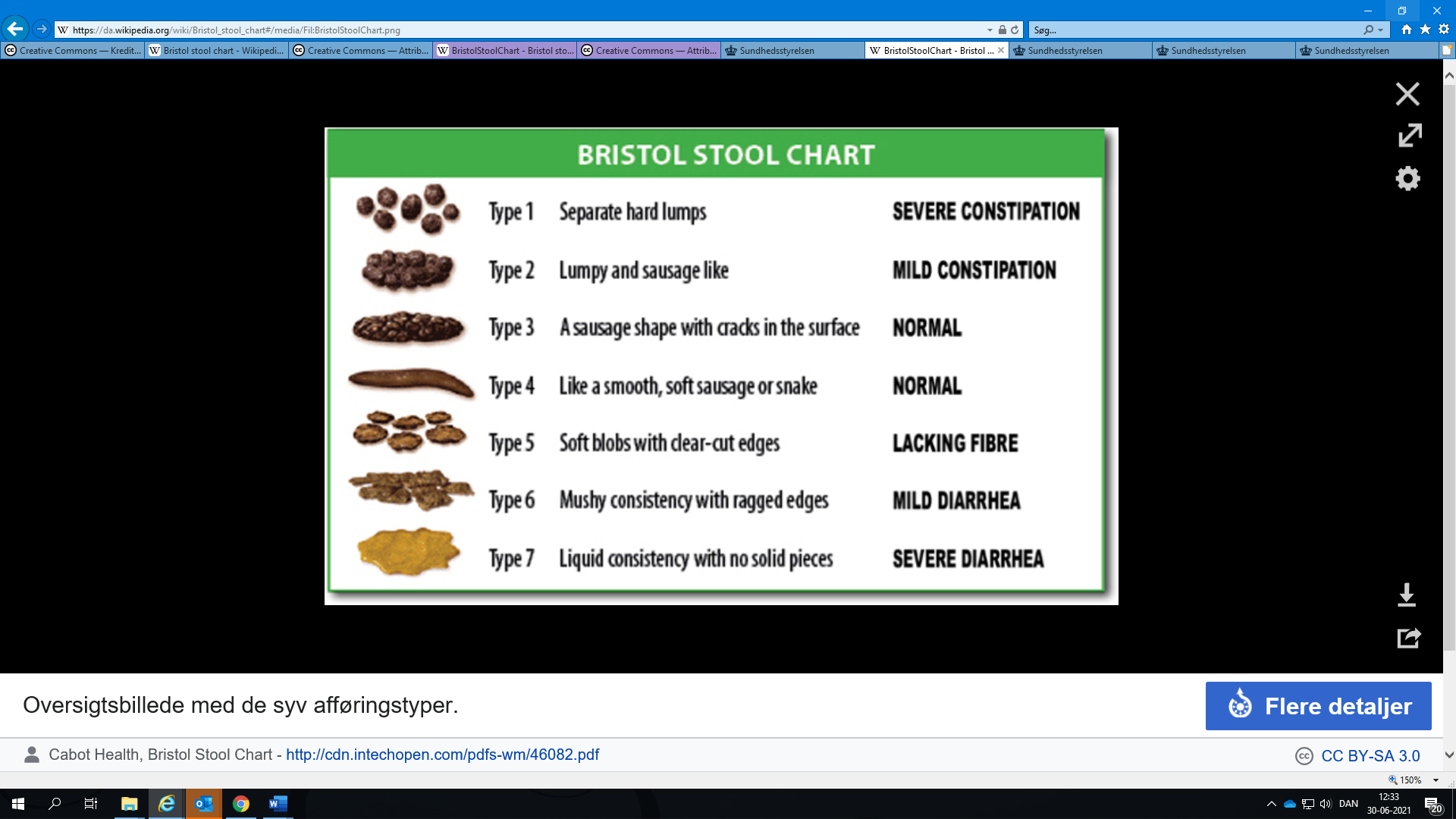 Mushy stool  Type 6 (fluffy small pieces,  ragged edged) |  |  |  |  |  |  |  |  |
| 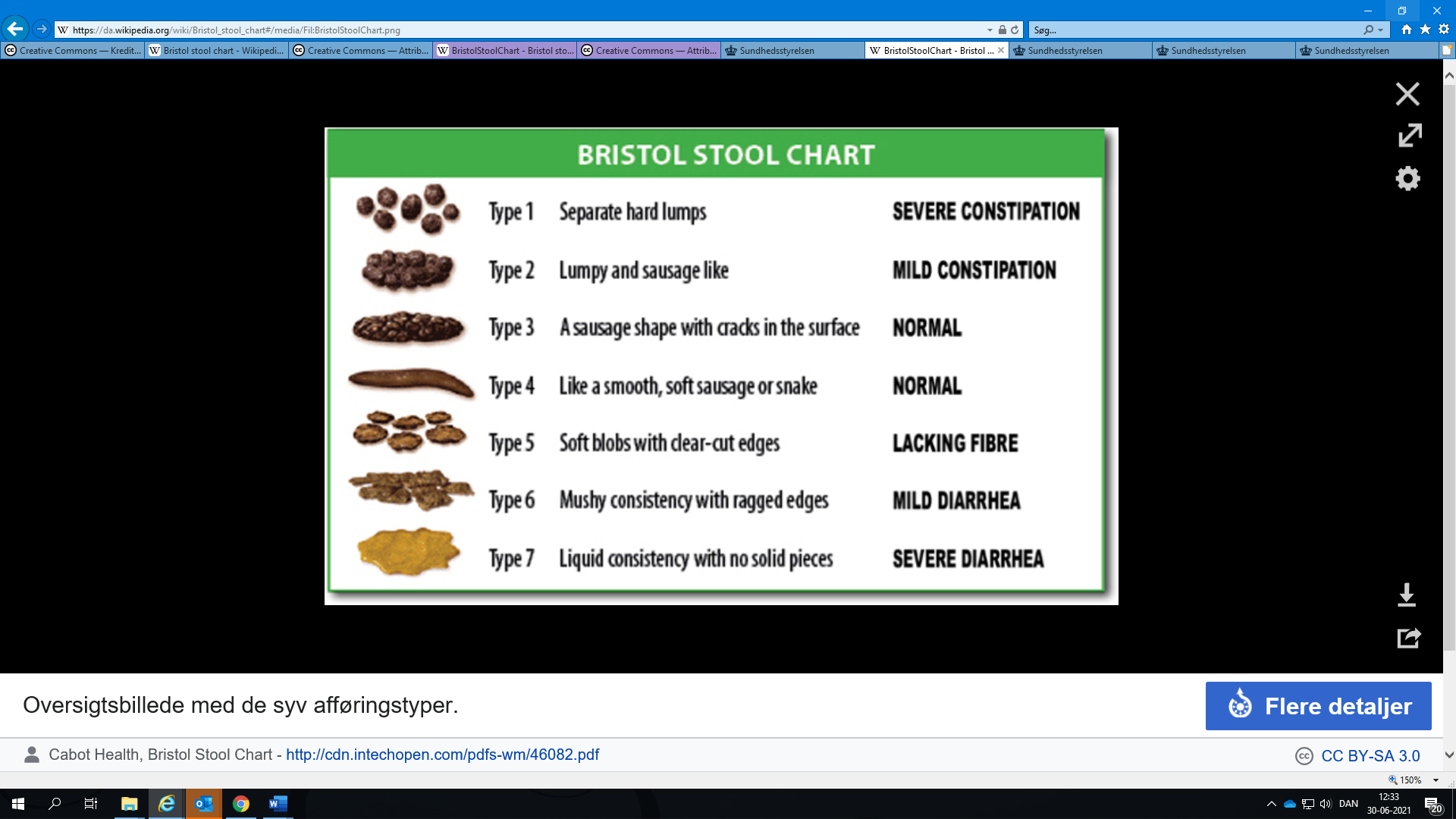  Type 7 Watery  (no solid pieces) |  |  |  |  |  |  |  |  |

**Week 8 after end of radiotherapy**

| **Diary Bristol scale for stool types** | | | | | | | | **Comments** |
| --- | --- | --- | --- | --- | --- | --- | --- | --- |
| **Date** |  |  |  |  |  |  |  |  |
| **Body weight** | **kg** |  |  |  |  |  |  |  |
| **Day of the week** | **day** | **day** | **day** | **day** | **day** | **day** | **day** |  |
| 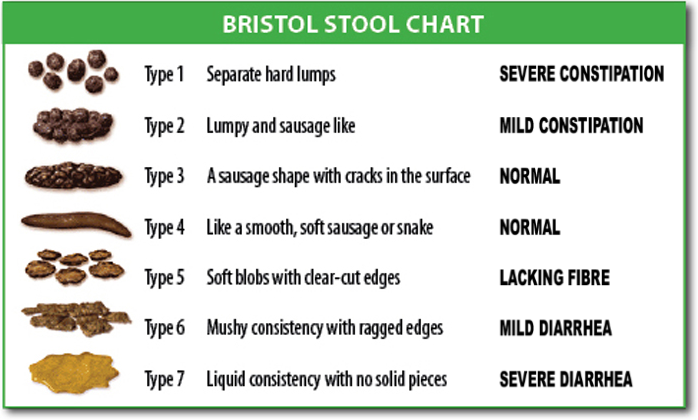  Type 1 Hard lumps |  |  |  |  |  |  |  |  |
| 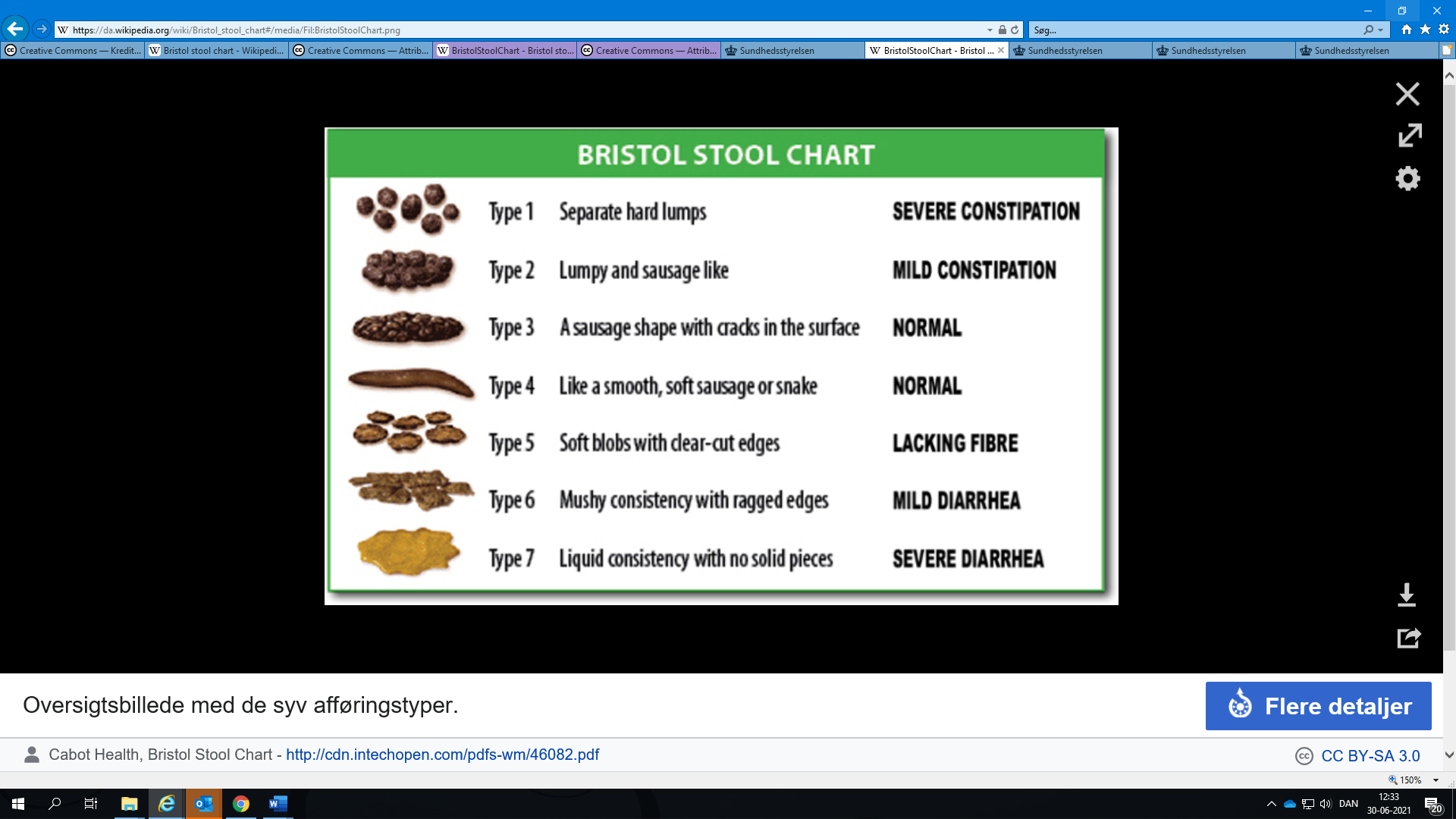  Type 2 Sausage-shaped  (lumpy surface) |  |  |  |  |  |  |  |  |
| 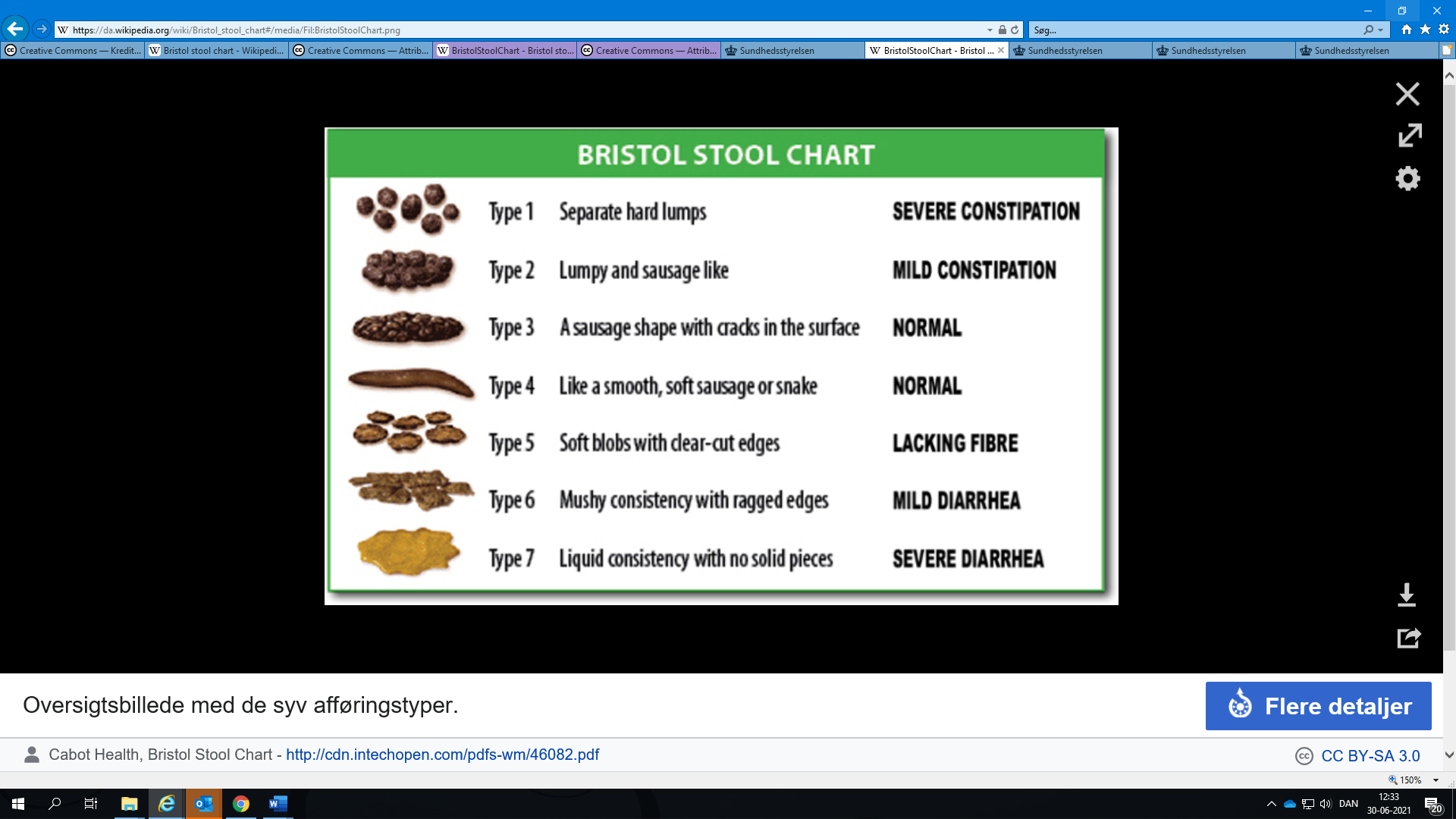  Type 3 Sausage-shaped  (cracked) |  |  |  |  |  |  |  |  |
| 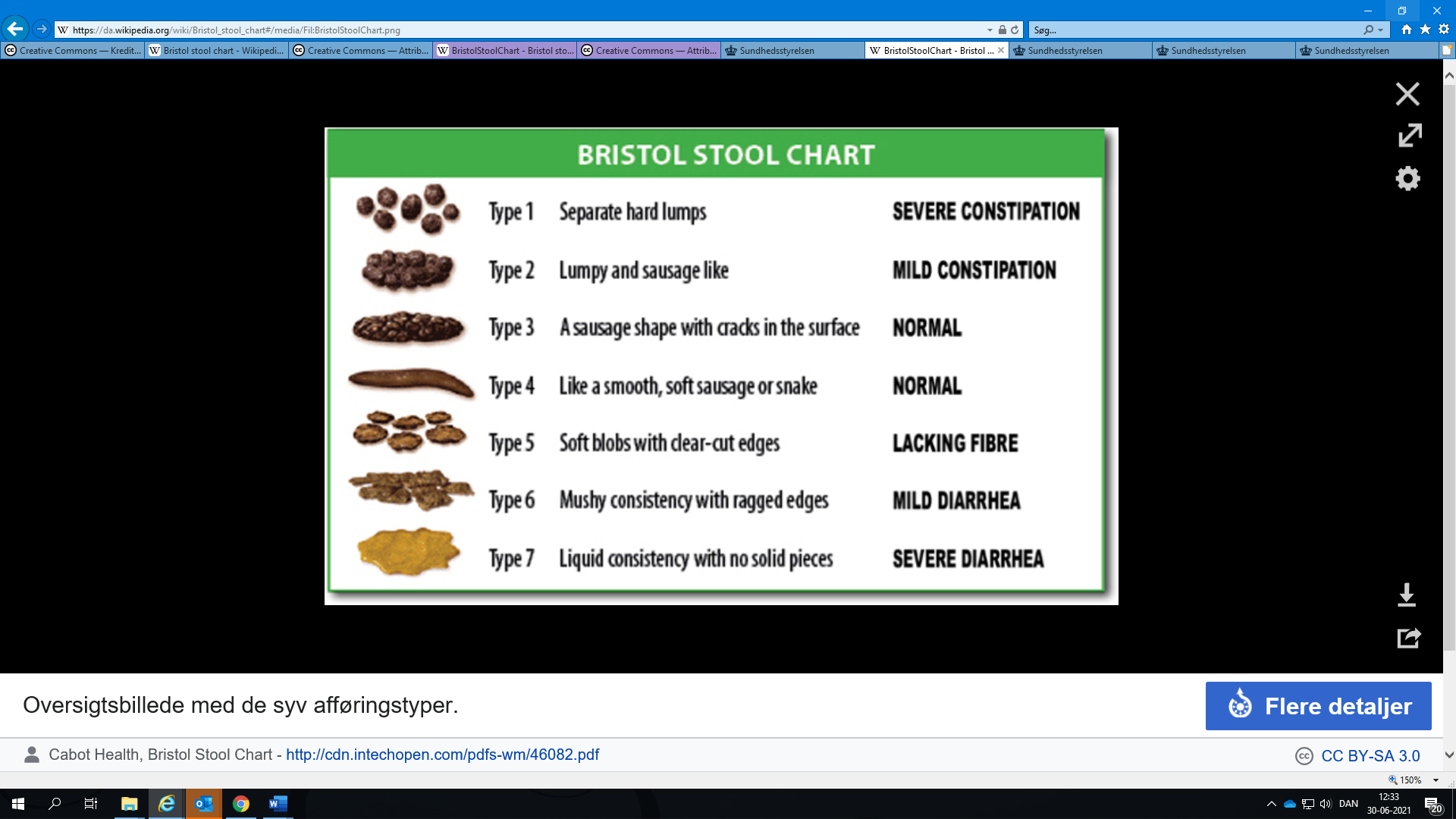  Type 4 Sausage or snake-shaped  (smooth and soft) |  |  |  |  |  |  |  |  |
| 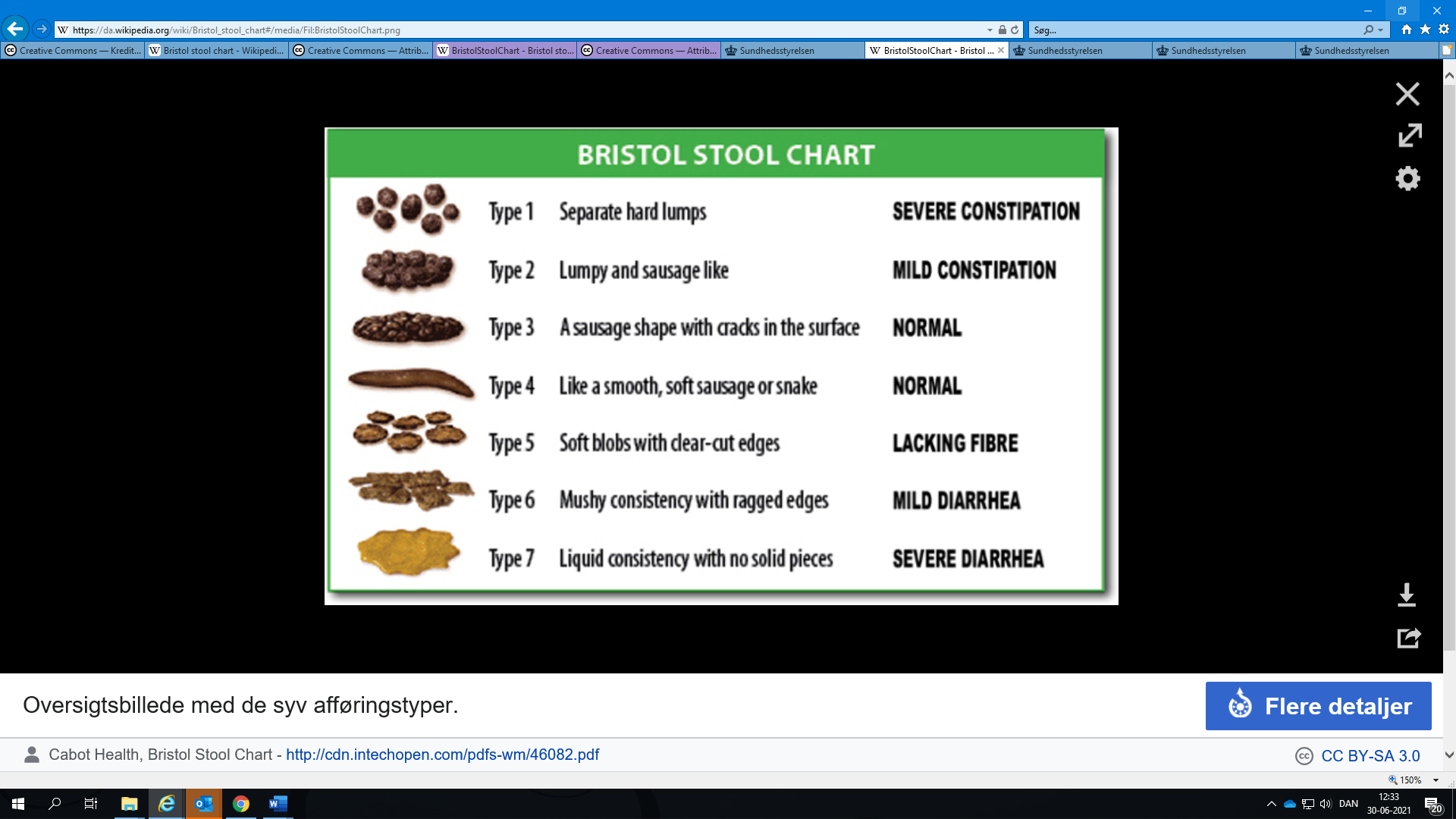  Type 5 Soft blobs  (clear cut edges) |  |  |  |  |  |  |  |  |
| 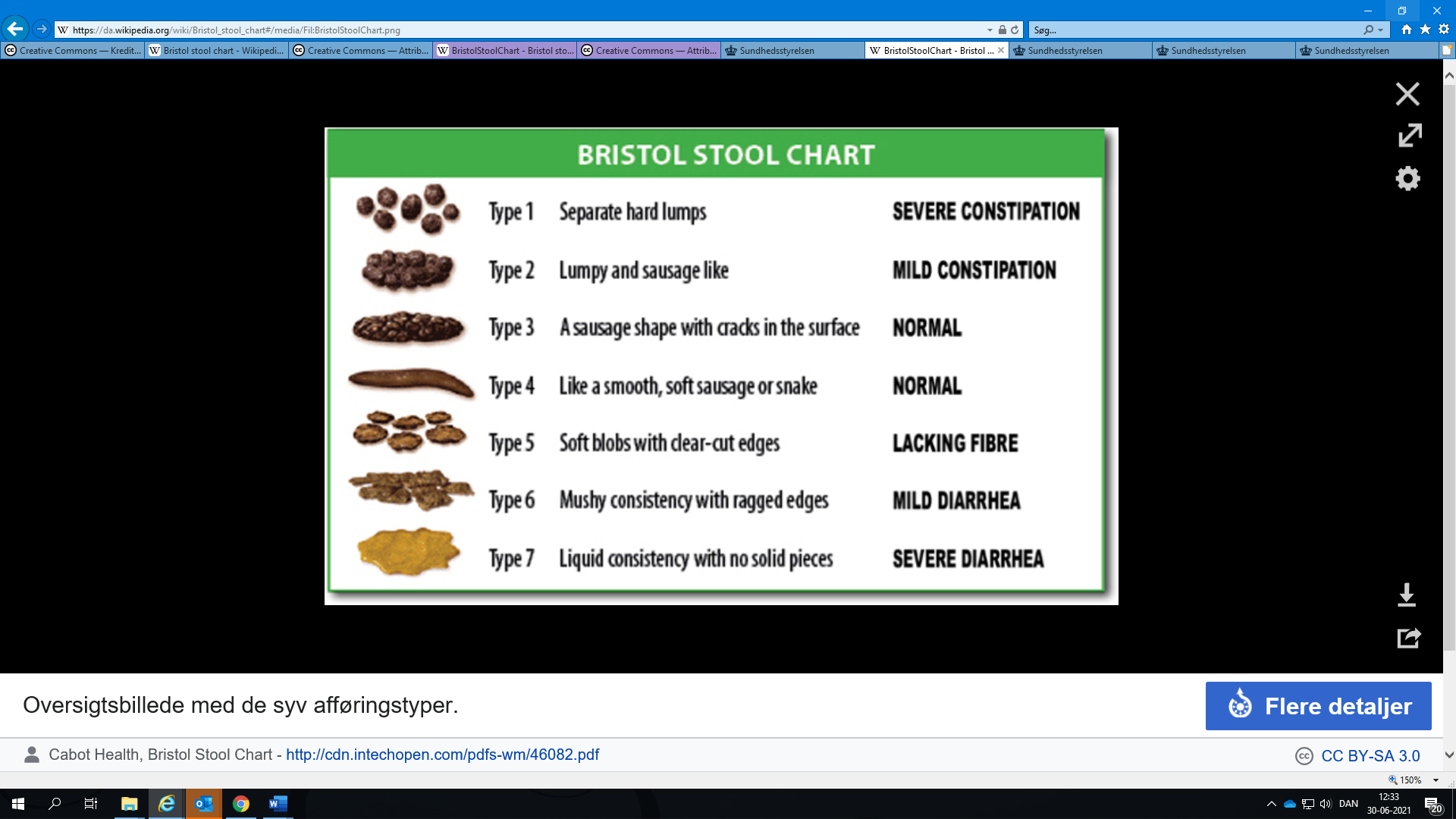 Mushy stool  Type 6 (fluffy small pieces,  ragged edged) |  |  |  |  |  |  |  |  |
| 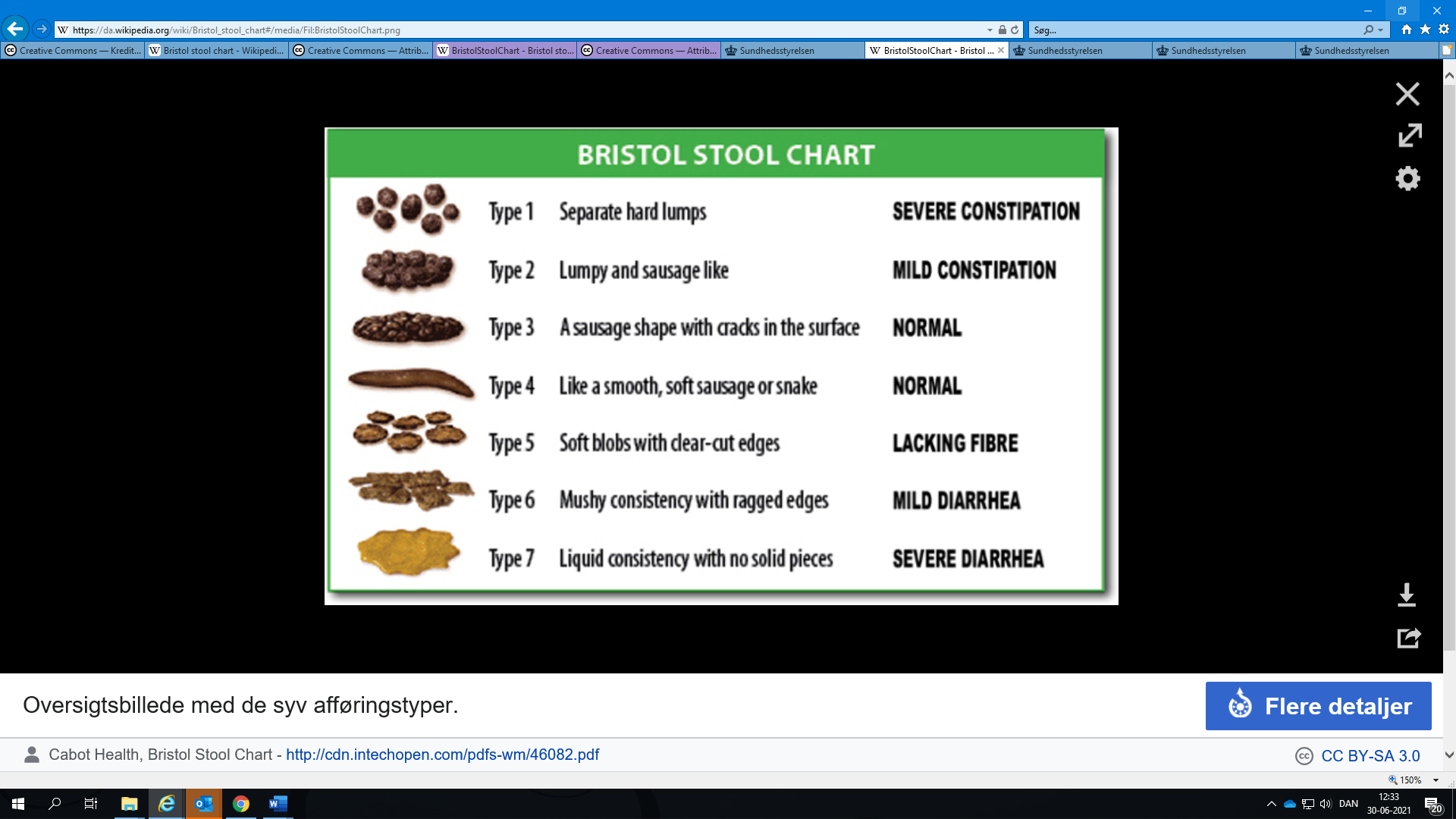  Type 7 Watery  (no solid pieces) |  |  |  |  |  |  |  |  |
