# Supplementary material for: Patient- and clinician-reported acute radiation-induced diarrhoea in patients with prostate cancer during curative external radiation therapy: A prospective observational cohort study
Source: J Patient Rep Outcomes. 2025 Dec 24;10:15. doi: 10.1186/s41687-025-00957-3 (PMC12847486; doi:10.1186/s41687-025-00957-3)
Supplement: Supplementary file 2 — Supplementary Material 2 [file 41687_2025_957_MOESM2_ESM.docx]

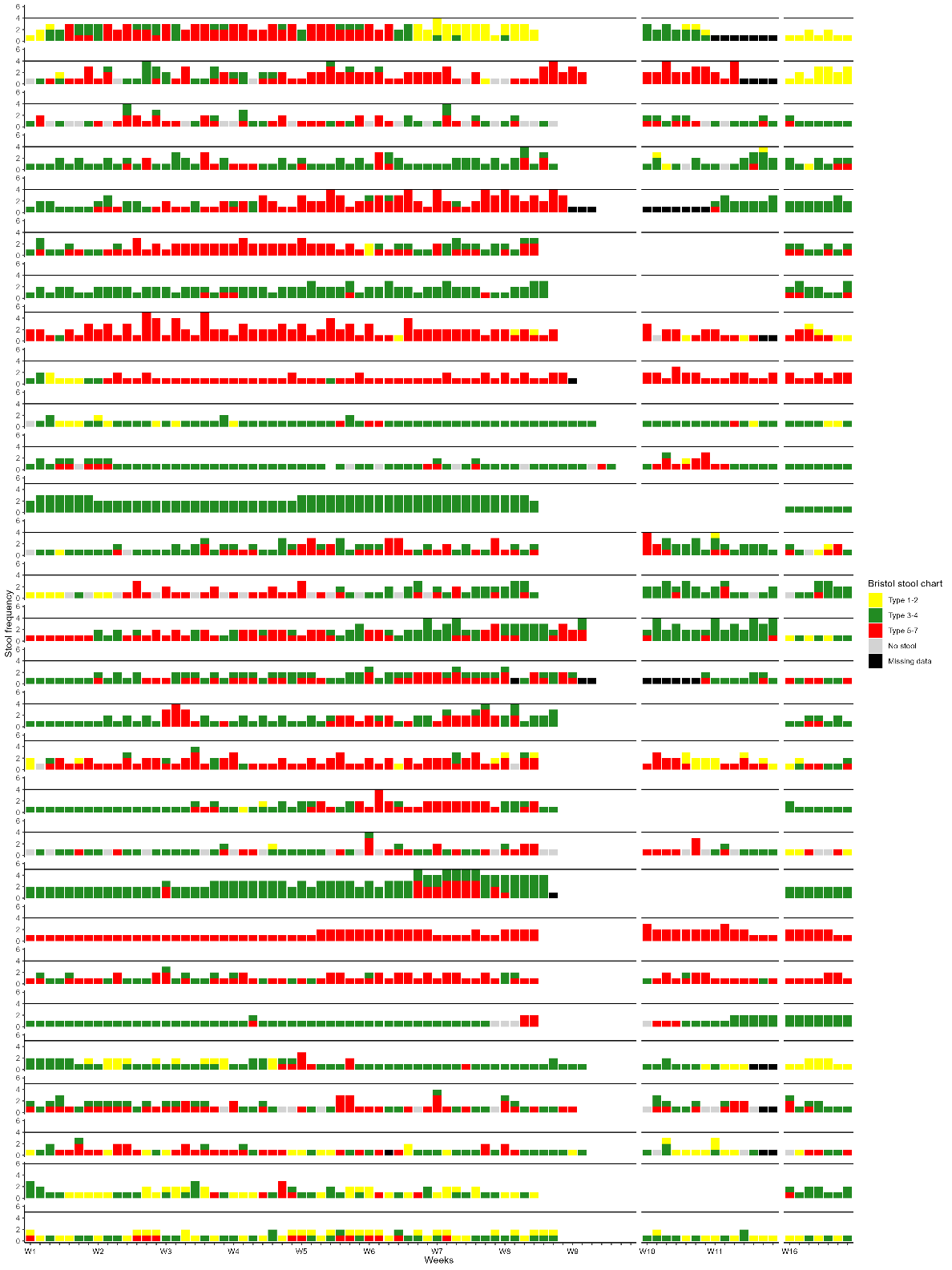


**Supplement 2** Frequency of RID for the 29 patients with Grade 0/1 according to Bristol Stool Chart

Bristol Stool Chart, Type 1-2: Yellow; Type 3-4: Green; Type 5-7: Red; No stool: Grey, Missing data: Black

Time points for PROM data were at baseline (week 1), at end of EBRT (week 8 or 9), 2 weeks after end of EBRT (week 10 or 11), and 8 weeks after end of EBRT (week 16 or 17)

The horizontal line symbolizes a frequency of stools ≥ 4 over baseline. The patients are sorted top-down: Patients with acute RID Grade 1 according to time of onset and duration, followed by patients with Grade 0

Abbreviation: W, Week
