# Supplementary material for: Patient- and clinician-reported acute radiation-induced diarrhoea in patients with prostate cancer during curative external radiation therapy: A prospective observational cohort study
Source: J Patient Rep Outcomes. 2025 Dec 24;10:15. doi: 10.1186/s41687-025-00957-3 (PMC12847486; doi:10.1186/s41687-025-00957-3)
Supplement: Supplementary file 3 — Supplementary Material 3 [file 41687_2025_957_MOESM3_ESM.docx]

**
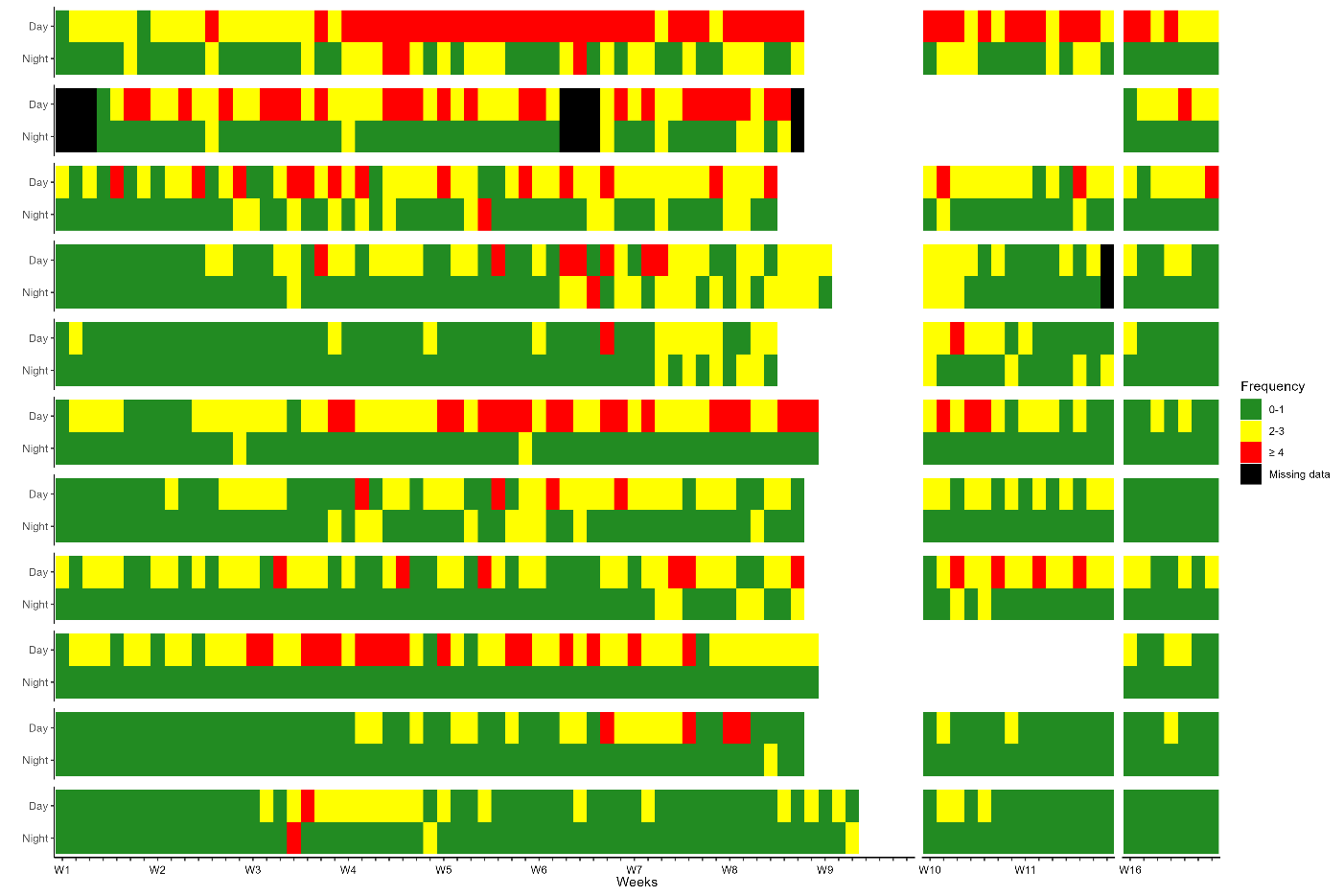
**

**Supplement 3** 24-hour registration of frequency of RID for the 11 patients with Grade 2/3

Measure points were daily from baseline (week 1) to end of EBRT (week 8 or 9), 2 weeks after end of EBRT (week 10 or 11), and 8 weeks after end of EBRT (week 16 or 17)

Daytime (06.00-21.59 hour) and nighttime (22.00-05.59 hour)

Stool frequency 0-1: Green; 2-3: Yellow, ≥ 4: Red; Missing data: Black

W: Week; FU: Follow up
