# Supplementary material for: Patient- and clinician-reported acute radiation-induced diarrhoea in patients with prostate cancer during curative external radiation therapy: A prospective observational cohort study
Source: J Patient Rep Outcomes. 2025 Dec 24;10:15. doi: 10.1186/s41687-025-00957-3 (PMC12847486; doi:10.1186/s41687-025-00957-3)
Supplement: Supplementary file 5 — Supplementary Material 5 [file 41687_2025_957_MOESM5_ESM.docx]

|  | **Baseline (week 1)** | | | **End of EBRT (week 8 or 9)** | | | **8 weeks after end of EBRT (week 16 or 17)** | | |
| --- | --- | --- | --- | --- | --- | --- | --- | --- | --- |
| RID Grade | **0/1 (n=29)** | **2/3 (n=11)** | **P-value** | **0/1 (n=29)** | **2/3 (n=11)** | **P-value** | **0/1 (n=29)** | **2/3 (n=11)** | **P-value** |
| **EORTC QLQ-PRT20** |  |  |  |  |  |  |  |  |  |
| **Question 35**  (category 1-4) |  |  |  |  |  |  |  |  |  |
| 1 | 28 (96.6%) | 11 (100%) | 1 | 24 (82.8%) | 8 (72.7%) | 0.379 | 24 (82.8%) | 10 (90.9%) | 1 |
| 2 | 1 (3.4%) | 0 (0%) |  | 4 (13.8%) | 3 (27.3%) |  | 3 (10.3%) | 1 (9.1%) |  |
| 3 | 0 (0%) | 0 (0%) |  | 0 (0%) | 0 (0%) |  | 0 (0%) | 0 (0%) |  |
| 4 | 0 (0%) | 0 (0%) |  | 0 (0%) | 0 (0%) |  | 0 (0%) | 0 (0%) |  |
| Missing |  |  |  | 1 (3.4%) | 0 (0%) |  | 2 (6.9%) | 0 (0%) |  |
| Question 41  (category 1-4) |  |  |  |  |  |  |  |  |  |
| 1 | 28 (96.6%) | 11 (100%) | 1 | 21 (72.4%) | 4 (36.4%) | 0.059 | 23 (79.3 %) | 11 (100%) | 0.673 |
| 2 | 1 (3.4%) | 0 (0%) |  | 5 (17.2%) | 5 (45.5%) |  | 3 (10.3%) | 0 (0%) |  |
| 3 | 0 (0%) | 0 (0%) |  | 1 (3.4%) | 2 (18.2%) |  | 1 (3.4%) | 0 (0%) |  |
| 4 | 0 (0%) | 0 (0%) |  | 1 (3.4%) | 0 (0%) |  | 0 (0%) | 0 (0%) |  |
| Missing |  |  |  | 1 (3.4%) | 0 (0%) |  | 2 (6.9%) | 0 (0%) |  |
| **Question 44**  (category 1-4) |  |  |  |  |  |  |  |  |  |
| 1 | 29 (100%) | 11 (100%) | 1 | 21 (75.9%) | 1 (9.1%) | <0.001 | 23 (79.3%) | 11 (100%) | 0.303 |
| 2 | 0 (0%) | 0 (0%) |  | 6 (20.7%) | 8 (72.7%) |  | 4 (13.8%) | 0 (0%) |  |
| 3 | 0 (0%) | 0 (0%) |  | 0 (0%) | 2 (18.7%) |  | 0 (0%) | 0 (0%) |  |
| 4 | 0 (0%) | 0 (0%) |  | 1 (3.4%) | 0 (0%) |  | 0 (0%) | 0 (0%) |  |
| Missing | 0 (0%) | 0 (0%) |  | 1 (3.4%) | 0 (0%) |  | 2 (6.9%) | 0 (0%) |  |

**Supplement 5** Urgency and faecal incontinence in the 29 patients with RID Grade 0/1 and the 11 patients with RID Grade 2/3

Question 35 is asking: Have you had any unintentional release (leakage) of liquid stools?, 41: Have you been unable to wait 15 minutes to open your bowels?, and 44: Have you had difficulty going out of the house, because you needed to be close to a toilet, because of bowel problems?

The symptoms were categorised: 1. not at all, 2. a little, 3. quite a bit, and 4. very much

Fisher’s exact test was used to analyse the association between PRO RID grade and urgency and faecal incontinence
