# Supplementary material for: Patient- and clinician-reported acute radiation-induced diarrhoea in patients with prostate cancer during curative external radiation therapy: A prospective observational cohort study
Source: J Patient Rep Outcomes. 2025 Dec 24;10:15. doi: 10.1186/s41687-025-00957-3 (PMC12847486; doi:10.1186/s41687-025-00957-3)
Supplement: Supplementary file 6 — Supplementary Material 6 [file 41687_2025_957_MOESM6_ESM.docx]

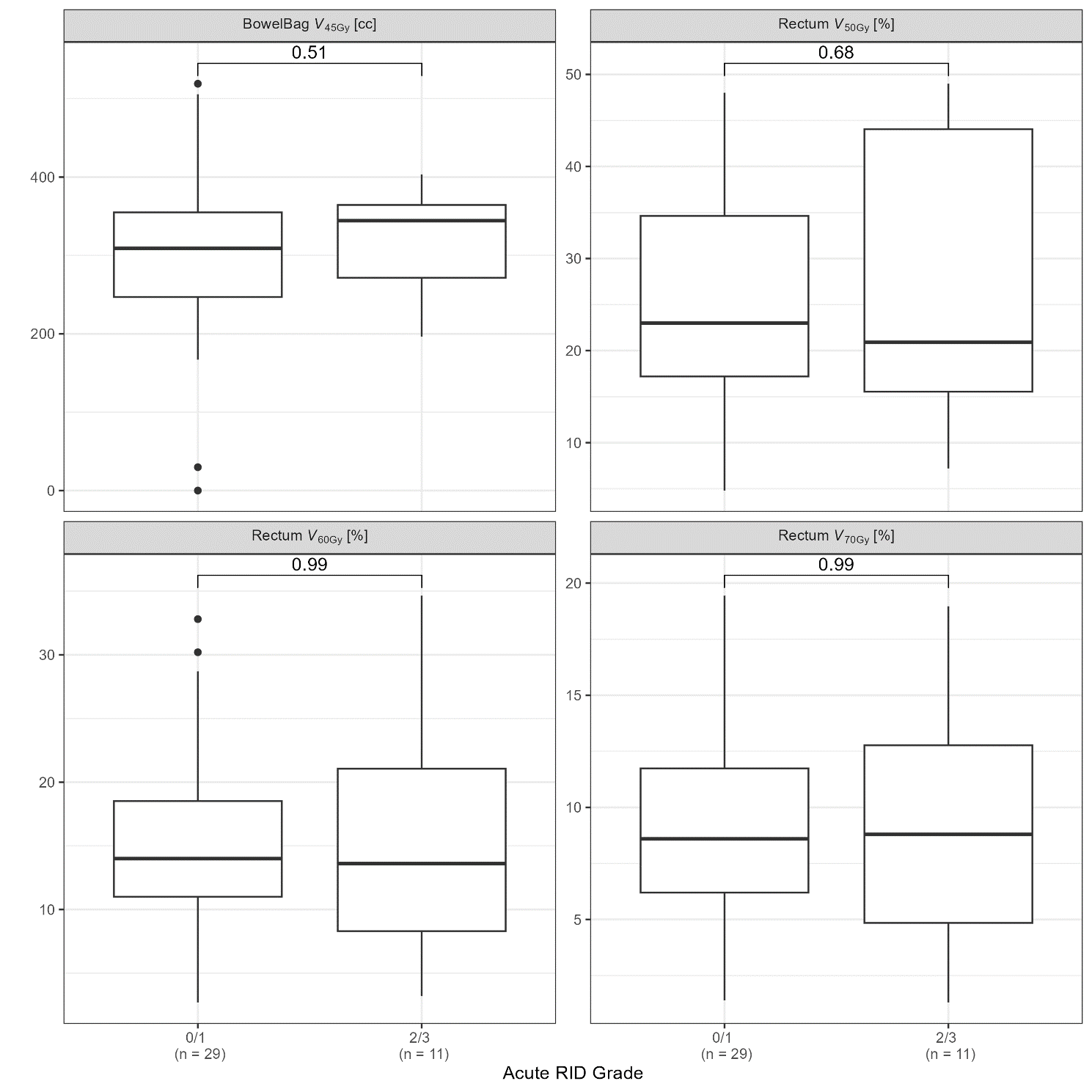


**Supplement 5**

Irradiation data for patients with RID Grade 2/3 vs 29 patients with Grade 0/1

There was no statistical significance between the EBRT data (BowelBag *V*_45Gy_, rectum *V*_50Gy_, *V*_60Gy_, *V*_70Gy_) and the 11 patients with acute RID Grade 2 or 3 vs the 29 patients with Grade 0 or 1

Abbreviations: V, volume; cc, cubic centimeter
